# Spindle Architectural Features Must Be Considered Along With Cell Size to Explain the Timing of Mitotic Checkpoint Silencing

**DOI:** 10.3389/fphys.2020.596263

**Published:** 2021-01-28

**Authors:** Mathew Bloomfield, Jing Chen, Daniela Cimini

**Affiliations:** Department of Biological Sciences and Fralin Life Sciences Institute, Virginia Tech, Blacksburg, VA, United States

**Keywords:** mitosis, cell size, nuclear size, mitotic spindle, mitotic checkpoint, tetraploidy, SAC

## Abstract

Mitosis proceeds through a defined series of events that is largely conserved, but the amount of time needed for their completion can vary in different cells and organisms. In many systems, mitotic duration depends on the time required to satisfy and silence the spindle assembly checkpoint (SAC), also known as the mitotic checkpoint. Because SAC silencing involves trafficking SAC molecules among kinetochores, spindle, and cytoplasm, the size and geometry of the spindle relative to cell volume are expected to affect mitotic duration by influencing the timing of SAC silencing. However, the relationship between SAC silencing, cell size, and spindle dimensions is unclear. To investigate this issue, we used four DLD-1 tetraploid (4N) clones characterized by small or large nuclear and cell size. We found that the small 4N clones had longer mitotic durations than the parental DLD-1 cells and that this delay was due to differences in their metaphase duration. Leveraging a previous mathematical model for spatiotemporal regulation of SAC silencing, we show that the difference in metaphase duration, i.e., SAC silencing time, can be explained by the distinct spindle microtubule densities and sizes of the cell, spindle, and spindle poles in the 4N clones. Lastly, we demonstrate that manipulating spindle geometry can alter mitotic and metaphase duration, consistent with a model prediction. Our results suggest that spindle size does not always scale with cell size in mammalian cells and cell size is not sufficient to explain the differences in metaphase duration. Only when a number of spindle architectural features are considered along with cell size can the kinetics of SAC silencing, and hence mitotic duration, in the different clones be explained.

## Introduction

Mitosis requires the completion of specific events in a timely manner; the precise sequence of events governs progression through defined mitotic stages (Baudoin and Cimini, 2018). Although these mitotic stages are conserved in different cell types and organisms, the time scale for their completion varies, with mitosis typically lasting 10–20 min in *Drosophila* embryos (McCleland et al., 2009), *S. pombe* (Krüger et al., 2019), and *S. cerevisiae* (Brewer et al., 1984; Leitao and Kellogg, 2017), 20–60 min in many mammalian cell lines (Rieder et al., 1994; Meraldi et al., 2004; Arnaoutov et al., 2005; Kuznetsova et al., 2015; Viganó et al., 2018), and 1–2 h in mouse embryos (Sikora-Polaczek et al., 2006). In many systems, mitotic duration critically depends on the spindle assembly checkpoint (SAC), the surveillance mechanism that monitors kinetochore-microtubule attachments and halts mitotic progression until all kinetochores are bound to spindle microtubules (Musacchio, 2011). There are two key SAC-regulated events whose duration can influence mitotic timing. First, the time it takes to satisfy the SAC by establishing kinetochore-microtubule attachments and chromosome biorientation (Gorbsky et al., 1998; Hauf et al., 2003; Mogilner and Craig, 2010; Foley and Kapoor, 2013; Sacristan and Kops, 2015). This process defines the stage of mitosis known as prometaphase, during which SAC signaling remains active. Second, the time it takes for the SAC to be silenced after complete chromosome alignment at the metaphase plate has been achieved (Rieder et al., 1994, 1995; Howell et al., 2000; Shah et al., 2004; Pereira and Maiato, 2012). This time would define the duration of metaphase. During prometaphase, unattached kinetochores serve as a platform to promote a conformational change in the SAC protein Mad2, which is then able to bind other SAC proteins in the cytoplasm and form the mitotic checkpoint complex (MCC), which in turn inhibits the anaphase promoting complex/cyclosome (APC/C), producing the so-called “wait-anaphase” signal (Taylor et al., 2004; Musacchio, 2015). This signal spreads throughout the spindle and cytoplasm (Heasley et al., 2017) and is capable of inducing a mitotic arrest in response to a single unattached kinetochore (Rieder et al., 1994, 1995). Once a kinetochore achieves stable attachment to microtubules, key SAC proteins are stripped from the attached kinetochore and transported poleward along spindle microtubules by the motor protein dynein (Howell et al., 2001), and SAC-activating phosphorylation events are reversed by phosphatases at the kinetochore (Etemad and Kops, 2016; Moura et al., 2017; Gelens et al., 2018; Saurin, 2018). Dynein-mediated transport is important for timely silencing of the SAC signal after all kinetochores achieve stable attachment to the microtubules (Howell et al., 2001; Griffis et al., 2007; Gassmann et al., 2010). SAC silencing leads to activation of the APC/C, degradation of securin and cyclin B, and consequently chromosome segregation and mitotic exit (Clute and Pines, 1999; Hagting et al., 2002). While stable kinetochore-microtubule attachments are sufficient to satisfy the SAC in mammalian cells (Tauchman et al., 2015), it is still unclear how robust and efficient silencing of the SAC is achieved.

Because SAC silencing involves trafficking SAC molecules among kinetochores, spindle, and cytoplasm, cell and spindle sizes are expected to affect mitotic duration by influencing the time required for SAC silencing. Recent studies have started to provide insight on some facets of this issue, but questions still remain. For instance, mitotic spindle size is thought to adjust to cell size (Heald and Gibeaux, 2018). This is evident in the early stages of embryogenesis, when developing cells rapidly divide without growth, producing cells of progressively smaller size with shorter spindles (Loughlin et al., 2010; Good et al., 2013; Hazel et al., 2013; Reber et al., 2013; Wilbur and Heald, 2013; Lacroix et al., 2018). Differences in genome size or ploidy may also affect spindle length indirectly by altering cell size (Mortimer, 1958; Mayer et al., 1992; Edgar and Orr-Weaver, 2001; Gregory, 2001; Gillooly et al., 2015). This has been observed in *Xenopus*, where cell and spindle sizes are larger in the near-tetraploid *X. laevis* compared to its diploid relative *X. tropicalis* (Brown et al., 2007; Loughlin et al., 2011). Furthermore, spindle scaling has been observed in a variety of metazoan species with different cell sizes (Crowder et al., 2015). However, in experimentally-generated tetraploid budding yeast strains, spindle length failed to scale with cell volume (Storchová et al., 2006), showing that we do not yet fully understand how the mitotic spindle scales in response to changes in genome and cell size. Similarly, the question of whether changes in cell/spindle size influence SAC silencing is still awaiting a definitive answer. A theoretical model predicts that spindle size scaling is important for robust SAC silencing (Chen and Liu, 2016). However, experimental investigation of this question has often been partial or indirect and has yielded contrasting observations. For instance, cell cycle and mitotic progression were not perturbed in tetraploid yeast cells (Storchová et al., 2006), which may be due to the lack of spindle scaling in those strains. In contrast, tetraploid mammalian cells often progress through mitosis more slowly than their diploid counterparts (Kuznetsova et al., 2015; Paim and FitzHarris, 2019; Cohen-Sharir et al., 2020; Quinton et al., 2020), suggesting a possible delay in SAC silencing. Several recent studies, which explored the link between cell and spindle sizes and SAC function more closely (Gerhold et al., 2015, 2018; Galli and Morgan, 2016; Kyogoku and Kitajima, 2017), also produced conflicting results. Gerhold et al. showed that depleting HIM-10, a component of the NDC80 complex, increased spindle length and metaphase duration in *C. elegans*, and the metaphase delay was only observed in cells with functional Mad2 (Gerhold et al., 2015). Conversely, mouse oocytes with excessively large cytoplasmic and spindle volumes progressed into anaphase more rapidly than smaller oocytes (Kyogoku and Kitajima, 2017). To further complicate the issue, many studies that investigate the relationship between cell size and the SAC are performed under conditions of mitotic spindle disruption (Galli and Morgan, 2016; Gerhold et al., 2018; Vázquez-Diez et al., 2019)—a method used to quantify SAC strength by measuring the time required for mitotic exit after depolymerization of spindle microtubules (Rieder and Maiato, 2004; Khodjakov and Rieder, 2009). This is especially common in studies examining SAC silencing, where Mps1 inhibition is combined with spindle disruption to avoid any effects kinetochore-microtubule attachments may have on the signaling events that prompt anaphase onset (Nijenhuis et al., 2014; Sivakumar et al., 2014; Saurin, 2018; Smith et al., 2019). Therefore, a thorough understanding of whether and how the time required for SAC silencing depends on cell and spindle sizes in the presence of an intact mitotic spindle is lacking. Moreover, previous studies have not investigated the contribution of other spindle architectural features, such as its shape and density, to SAC silencing and mitotic duration.

To study the effects of cell and spindle size on SAC silencing, we generated tetraploid (4N) clones from non-transformed, immortalized retinal pigmented epithelial (RPE-1) and DLD-1 colorectal cancer cells. Not all of the 4N DLD-1 clones displayed the same scaling of cell and spindle size compared to the diploid parental cells, which made them an ideal model to study the relationship between cell/spindle size and mitotic duration. We found that the 4N clones had unique spindle geometries and certain clones exhibited altered mitotic timings, which were due to differences in metaphase duration (i.e., time taken to silence the SAC after all kinetochores are attached). Leveraging a previous mathematical model for spatiotemporal regulation of SAC silencing (Chen and Liu, 2014, 2016), we show that the differences in SAC silencing time, and hence metaphase duration, cannot be explained by cell and spindle size alone. Instead, only when other spindle architectural features, including spindle pole size and microtubule abundance, are considered along with spindle and cell size can the timing of SAC silencing and metaphase duration be explained in all clones. Lastly, in line with a model prediction, we demonstrate that experimentally manipulating spindle geometry can reduce metaphase duration in a clone characterized by a particularly long metaphase.

## Materials and Methods

### Cell Lines and Culture Conditions

DLD-1 (ATCC CCL-221) and hTERT-immortalized RPE-1 cells were obtained from the American Type Culture Collection (ATCC, Manassas, VA). The DLD-1 cells were maintained in RPMI 1640 media with ATCC modification (Thermo Fisher Scientific—Gibco, CA, USA) supplemented with 10% fetal bovine serum (FBS; Thermo Fisher Scientific) and 1% antibiotic-antimycotic (Thermo Fisher Scientific), and the RPE-1 cells were maintained in a 1:1 mixture of DMEM/F-12 with HEPES (Thermo Fisher Scientific). All cells were kept in a humidified incubator at 37°C and 5% CO_2_ and monitored for possible mycoplasma infection every 2–3 weeks by DNA staining.

Tetraploid RPE-1 and DLD-1 cells were generated by treating diploid cells with 1.5 μg/mL dihydrocytochalasin B (DCB; Sigma Aldrich, St. Louis, MO) for 20 hrs. After treatment, the cells were washed four times with cell culture media and allowed to grow an additional 1–2 days in supplemented media before the isolation of single cells by limiting dilution in 96-well-plates. Only wells containing a single cell were expanded into clonal cell lines and used for further experimentation. Characterization of 4N clones and subsequent experiments were performed at low passages to limit variability due to evolution of the cell population.

For experiments conducted in the presence of blebbistatin (Tocris Bioscience, Bristol, UK), the drug was added to the media at a final concentration of 10 nM for 4 h before fixation and immunostaining or at the time of media replacement prior to live-cell imaging.

### Preparation of Chromosome Spreads and Chromosome Counting

Cells were grown to 70–80% confluency in T-25 flasks and 50 ng/ml colcemid (Karyomax–Invitrogen, Waltham, MA) was added to the media for 4–6 h to enrich for mitotic cells. The cells were collected by trypsinization and centrifuged at 1,000 rpm for 5 min. The cell pellet was resuspended in 2 mL PBS and centrifuged for 3 min at 1,000 rpm. Five milliliters of hypotonic solution (0.075 M KCl) was added to the cell pellet and incubated for 18 min at 37°C; then 0.5 mL of freshly prepared fixative (3:1 methanol:glacial acetic acid) was added before centrifugation at 1,000 rpm for 5 min. After removal of the supernatant, the cell pellet was resuspended by adding 5 mL of fixative dropwise and then incubated at room temperature for 15 min before centrifugation at 1,000 rpm for 5 min. Depending on the size, resulting cell pellets were suspended in 0.3–6 mL fixative (added dropwise) and 12 μL of the cell suspension were dropped onto microscope slides to check for optimal density and spreading. Chromosome spreads were left at room temperature overnight and stained the next day with 300 nM DAPI (Invitrogen) for 10 min. Antifade solution (90% glycerol and 0.5% N-propyl gallate) was added to the slides and sealed under a 22 × 50 mm coverslip (Corning Incorporated, Corning, NY) with nail polish. Chromosome spreads were imaged using a Nikon Eclipse Ti inverted microscope (Nikon Instruments Inc., NY, USA) equipped with ProScan automated stage (Prior Scientific, Cambridge, UK), CoolSNAP HQ2 CCD camera (Photometrics, AZ, USA), Lumen200PRO light source (Prior Scientific), and a 60X/1.4 NA Plan-Apochromatic objective. Chromosomes were counted in individual images of chromosome spreads using NIS Elements AR version 4.60 software (Nikon Instruments Inc.) to confirm cell ploidy.

### Cell and Nuclear Volume Measurements

For cell and nuclear volume measurement, cells were synchronized in G2 to reduce variation in size due to cell cycle stage. Cells were seeded onto sterilized acid-washed glass coverslips inside 35 mm Petri dishes at low densities to enable cells to adhere without touching neighbors. The next day, 9 μM of the CDK-1 inhibitor RO-3306 (Sigma Aldrich) (Vassilev, 2006) was added to the media for 18 h to synchronize cells in G2. After synchronization, the cells were incubated in serum-free media containing 5 μM CellTracker Green CMFDA Dye (Thermo Fisher Scientific) for at least 30 min to stain the cytoplasm. The serum-free medium was replaced with medium containing FBS for at least 30 min before fixation per the manufacturer's protocol. Cells were fixed in ice-cold methanol for 10 min at −20°C, washed three times (5 min each) with PBS, and counterstained with 300 nM DAPI (Invitrogen) for 5 min. Coverslips were mounted on microscope slides in an antifade solution and sealed with nail polish. Z-stack images spanning the entire height of single cells were acquired at 0.6 μm steps with a swept field confocal system (Prairie Technologies, WI, USA) on a Nikon Eclipse TE2000 inverted microscope equipped with a 60X/1.4 NA Plan-Apochromatic lens, motorized ProScan stage (Prior Scientific), an XCITE 120Q light source (Excelitas Technologies, Waltham, MA, USA; used to view cells before imaging), a CoolSNAP HQ2 CCD camera (Photometrics), an Agilent monolithic laser combiner (MLC400) controlled by a four channel acousto-optic tunable filter, and a multiband pass filter set (illumination at 405, 488, 561, and 640 nm). Cell and nuclear volume measurements were performed in FIJI [ImageJ, NIH (Rasband, 2011)] using a macro for three-dimensional reconstruction. In brief, image smoothing was done using a Gaussian function, a binary image of each Z-stack was created by auto thresholding (v1.17.2, “default” FIJI algorithm) with the stack histogram, any holes were filled, and the “3D Object Counter” was used to quantify 3D objects in each stack and determine cell and nuclear volumes (Bolte and Cordelières, 2006).

### Immunofluorescence, Image Acquisition, and Data Analysis

For spindle dimension measurements, prometaphase-metaphase defect analysis, and mitotic stage analysis, cells were fixed in freshly prepared 4% paraformaldehyde in PHEM buffer (60 mM Pipes, 25 mM HEPES, 10 mM EGTA, 2 mM MgSO_4_, pH 7.0) for 20 min at room temperature. Cells were then washed three times (5 min each) with PBS and permeabilized in PHEM buffer containing 0.5% Triton-X 100 for 10 min at room temperature. After three quick washes with PBS, cells were blocked with 20% boiled goat serum for 1 h at room temperature. For cyclin B immunostaining and α- and γ-tubulin fluorescence intensity measurements, cells were fixed using ice-cold methanol for 10 min at −20°C and washed three times with PBS. For γ-tubulin, cells were blocked for 1 h at room temperature with 10% BGS. For cyclin B and α-tubulin, 20% BGS was used for blocking. Next, the cells were incubated overnight at 4°C with primary antibodies in 5% BGS and PHEM buffer. Cells were then washed four times in PBS-T (PBS with 0.05% Tween 20) before 45 min incubation at room temperature with secondary antibodies in 5% BGS and PHEM buffer. The following primary antibodies and dilutions were used: mouse anti-centrin (Abnova, Zhongli, Taiwan), 1:200; rabbit anti-α-tubulin (Abcam, Cambridge, MA), 1:250; human anti-centromere protein (Antibodies Inc., Davis, CA), 1:100; mouse anti-α-tubulin (DM1A, Sigma Aldrich), 1:500; rabbit anti-γ-tubulin (Abcam), 1:200; mouse anti-cyclin B (BD Biosciences, San Jose, CA), 1:500. The following secondary antibodies and dilutions were used: Alexa 488 goat anti-rabbit (Molecular Probes, Life Technologies, CA, USA), 1:200; Rhodamine Red-X goat anti-human (Jackson ImmunoResearch Laboratories, Inc.,

$$\begin{array}{r} \text{SpindleBackground Intensity}=\text{average background intensity }\times \text{ spindle area}\\ \text{SpindleMicrotubule Intensity }=\text{ Total α}-\text{tubulin Intensity}-\text{ Spindle Background Intensity} \end{array}$$

West Grove, PA), 1:100; Cy5 goat anti-mouse (Abcam), 1:200; Alexa 488 goat anti-mouse (Molecular Probes), 1:200.

To analyze the mitotic spindle, Z-stack images were acquired at 0.6 μm steps using the Nikon Eclipse TE2000 microscope setup described in the previous section. For spindle width, a maximum projection image was used to determine the distance between the outermost kinetochores in the metaphase plate by drawing and measuring a straight line in FIJI. For spindle length, a straight line was drawn and measured from the most intense centrin signal at each spindle pole. For cells where the spindle poles were not in the same focal plane, spindle length was calculated using the Pythagorean theorem. Spindle height was determined as the distance from the first to the last plane of the Z planes containing kinetochores. Spindle volume was calculated as: π/12 × spindle length × spindle width × spindle height.

For quantification of cells with unaligned chromosomes, the analysis was limited to late prometaphase cells (i.e., cells with a well-defined metaphase plate and only 1-few unaligned chromosomes). For analysis of multipolarity, analysis was limited to cells in which a metaphase plate was discernible. For mitotic stage analysis, the number of mitotic cells in prophase, prometaphase, metaphase, anaphase, and telophase were counted (Baudoin and Cimini, 2018).

For fluorescence intensity analysis, images for all cell lines in each experiment were captured on the same day using the same microscope and imaging settings and were analyzed using FIJI software. For quantification of γ-tubulin intensity, Z planes containing a detectable γ-tubulin signal (as determined by the default auto thresholding function in FIJI) were converted to a single image using a sum intensity projection. 17-pixel (*I*_*a*_) and 24-pixel (*I*_*b*_) diameter circles centered around the γ-tubulin signal were used to determine background-subtracted spindle pole fluorescence intensity using the following formula:

$$\begin{array}{l} \text{Spindle Pole Fluorescence Intensity }=\;{\text{I}}_{\text{a}}\;-\;\left({\text{I}}_{\text{b}}\;-\;{\text{I}}_{\text{a}}\right). \end{array}$$

For spindle microtubule measurements, auto thresholding of a maximum projection of *Z* planes spanning the entire spindle was performed to create a binary image. Any holes were filled and the “analyze particles” function was used to define the spindle area, which was saved as region of interest (ROI). Next, this ROI was overlaid onto a sum intensity projection of *Z* planes spanning the entire spindle height to measure the integrated density of all pixels within the spindle. For background correction, two identical ROIs were drawn outside of the spindle area, but within the cell, to measure intracellular background fluorescence. These measurements were averaged (total background fluorescence/total background ROI area), then this value was extrapolated to match spindle area, and subtracted from the α-tubulin spindle intensity using the following formulas:

### Live-Cell Phase Contrast Microscopy

Cells were grown on glass bottom dishes with No. 1.5 glass (MatTek Corporation, Ashland, MA). Immediately prior to imaging, cell medium was replaced with L-15 media (Gibco) supplemented with 4.5 g/L glucose. All live-cell experiments were performed on the same Nikon Eclipse Ti inverted microscope (Nikon Instruments Inc.) described earlier (“Preparation of chromosome spreads and chromosome counting”) with a temperature and humidity-controlled incubator (Tokai Hit, Japan). For live-cell phase contrast videos, images were acquired every 3 min using a 20X/0.3 NA A Plan corrected phase contrast objective for at least 6 h. Videos were analyzed with NIS Elements to determine mitotic duration, or the time from cell rounding to anaphase onset. In some cells, especially the larger 4N groups, nuclear envelope breakdown (NEBD) could also be observed and typically coincided with the beginning of cell rounding. For analysis of prometaphase and metaphase duration, only cells with visible metaphase plates were used for analysis. Prometaphase-metaphase transition was recorded as the time a metaphase plate could be seen at the spindle equator.

### Statistical Analysis

Statistical analysis was performed using GraphPad Prism (version 8.3.1) software. For prometaphase-metaphase defect and mitotic stage analysis, statistical significance was determined using a two-sided Fisher's exact test. For all other experiments, a Student's *t*-test was used to test for statistical significance. For experiments in which individual data points are reported, results from individual experiments were compared to each other for reproducibility and confirm that no statistical differences were observed between experiments.

### Mathematical Model for Spatiotemporal Regulation of SAC Silencing

The model for SAC silencing consists of a core model for the spatial dynamics of SAC proteins, and coupled biochemical circuits for SAC signaling and silencing (Chen and Liu, 2014). The core model predicts the concentration of SAC proteins at the spindle pole as increasing number of kinetochores establish attachment to the spindle and was built upon the following key assumptions motivated by experimental observations:

- High phosphorylation level at unattached kinetochores vs. low phosphorylation level at attached ones (Liu et al., 2009; Maresca and Salmon, 2009; Welburn et al., 2010) dictates the distinct dynamics and fates of SAC proteins at the two types of kinetochores. Because of the known interdependency between kinetochore attachment state, phosphorylation level of kinetochore proteins, and dynein-mediated transport (Whyte et al., 2008; Matson and Stukenberg, 2014), the model assumes that SAC proteins released from the attached and unattached kinetochores adopt distinct states. The SAC proteins released from an attached kinetochore assume a transport-active state, forming complexes with active dynein and undergoing poleward streaming, while those released from an unattached kinetochore assume a transport-inactive state with diffusion only. Moreover, because kinetochore phosphorylation level promotes recruitment of SAC proteins (Ditchfield et al., 2003), the model assumes a significant decrease in the recruitment rate of SAC proteins as a kinetochore turns to the attached state.
- The transport-active SAC proteins move along microtubules toward the spindle pole and they may unbind before reaching the pole due to dynein's limited processivity (King and Schroer, 2000; Reck-Peterson et al., 2006). While unbound from microtubules, these proteins diffuse in the cytoplasm and have a chance to either rebind the microtubules and keep moving toward the spindle pole or bind a kinetochore. Once these proteins reach the spindle pole, they are partially sequestered by the spindle pole through binding/unbinding dynamics.

The core model was formulated as a system of compartmentalized diffusion-advection-reaction partial differential equations (PDE) given in the Supplementary Materials (Chen and Liu, 2014, 2015, 2016). The core model was further expanded to an extended model, by coupling the spatial dynamics with a biochemical circuit that describes the regulatory interactions between key molecules of the SAC and a generic toggle switch circuit (Tyson et al., 2003) that is triggered by a threshold SAC protein concentration at the spindle pole and induces SAC silencing. The PDEs for the extended model are also provided in the Supplementary Materials.

### Size and Microtubule Parameters Used in the Model

Because cell/spindle sizes and mitotic timing must be measured with several different experimental setups and could not all be obtained from the same cell, we could only use the model to make predictions based on the average sizes in each clone. To avoid large deviation caused by outlier data points, we used the median values of the measured sizes and fluorescence intensities. The measured values and derived model parameters are summarized in Table 1.

**Table 1:** Median values of measured quantities in cell clones and parameters used in the model.

	**Quantity**	**DLD-1**	**S1**	**S2**	**L1**	**L2**	**RPE-1 (2N)**	**RPE-1 (4N)**
Measured	Cell Volume (μm^3^)	1,847	2,227	2,383	3,153	3,081	2,399	5,595
	Spindle length (μm)	6.83	7.27	10.33	9.86	12.20	11.82	12.23
	Spindle width (μm)	8.85	9.97	10.74	10.94	11.44	8.69	11.17
	Spindle height (μm)	6.60	9.00	9.60	7.80	6.60	6.00	7.80
	γ-tubulin intensity (a.u.)	864.81	1,595.0	888.87	1,373.8	1,309.1	934.87	999.97
	α-tubulin intensity (a.u.)	893.27	1,841.0	1,030.6	2,201.1	1,080.6	959.92	971.45
Model	Cell diameter (μm): $D=\sqrt[{3}]{measured\;cell\;volume\times 6÷\pi }$	15.22	16.20	16.57	18.19	18.05	16.61	22.03
	Spindle length (μm): *L* = *measured spindle length*	6.83	7.27	10.33	9.86	12.20	11.82	12.23
	Spindle width/height (μm): *W*=$\sqrt{\frac{measured\;spindle\;width\times }{measured\;spindle\;height}}$							
	7.64	9.47	10.15	9.24	8.69	7.22	9.33	
	Spindle pole diameter (μm), *d* [see Supplementary Material, Eq. (S1)]	1.11	1.50	1.12	1.39	1.36	1.57	1.62
	Number of kinetochore microtubules: *N*_*MT*1_ (see Supplementary Material, Eq. (S2))	1,600	3,193	916	2,885	960	1,032	776

## Results

**Figure 1:**
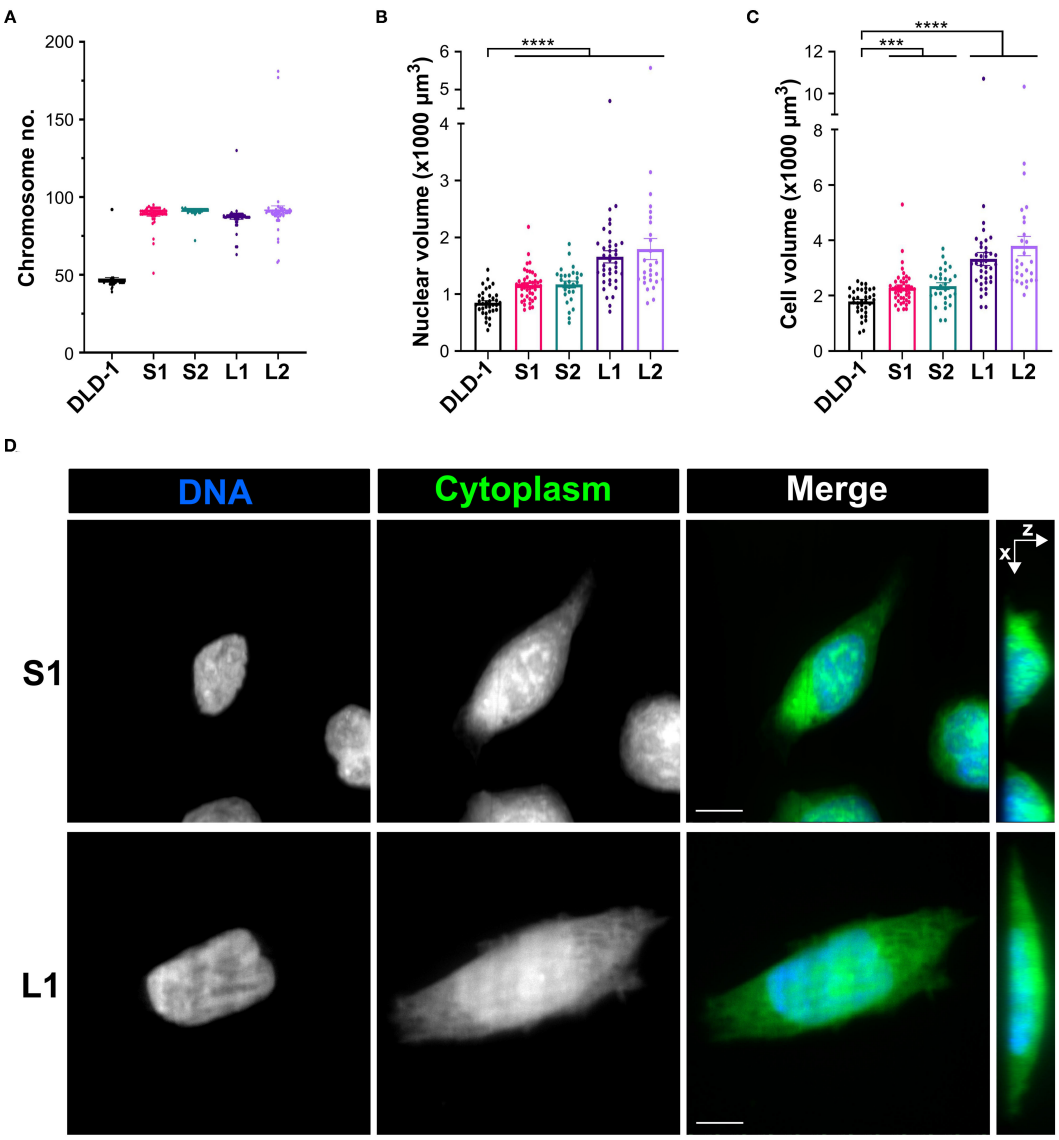
Tetraploidization has a variable effect on cell and nuclear volume in DLD-1 cells. **(A)** Analysis of chromosome number to confirm ploidy in DLD-1 cells and derived tetraploid clones (*n* = 50 for each). **(B)** Nuclear and **(C)** cell volume measurements in parental DLD-1 cells and 4N DLD-1 clones synchronized in G2. Data are reported as mean ± SEM with individual data points from three independent experiments in which a total of 27–44 cells (*n* = 35, 44, 28, 39, and 27, respectively) were analyzed. ***p < 0.001, ****p < 0.0001, when compared to the parental DLD-1 cells by Student's *t*-test. **(D)** Examples of S1 (top) and L1 (bottom) cells used for nuclear (blue) and cell (green) volume analysis. Images are maximum intensity projections from *Z*-stacks along the *X*–*Y* coordinates (left three columns) or along the *X*–*Z* coordinates (right column). All scale bars, 10 μm.

Since tetraploidy may have disparate effects on cell and spindle size that disrupt mitotic fidelity (Storchová et al., 2006), we reasoned it could be used to study the relationship between cell size, mitotic spindle geometry, and SAC function. Therefore, we induced cytokinesis failure with dihydrocytochalasin B in RPE-1 (diploid, non-transformed) and DLD-1 cells (pseudodiploid colorectal cancer cells) and expanded clonal cell lines from single tetraploid (4N) cells. We were able to isolate one 4N RPE-1 clone and several 4N DLD-1 clones. We next characterized these 4N clones to determine nuclear and cell volumes in G2-synchronized cells. In RPE-1 cells, cell and nuclear volume scaled with DNA content (Supplementary Figures 1A,B, Table 2). For DLD-1 cells, however, we found that, although chromosome number doubled in each DLD-1 4N clone (Figure 1A, Table 2), cell and nuclear volume did not scale uniformly (Figures 1B–D, Table 2). Two of the DLD-1 4N clones were characterized by small nuclear and cell size (S clones) and two were characterized by large sizes (L clones). Specifically, the nuclear volume of L1 and L2 increased by 96 and 95%, respectively, compared to parental DLD1 cells, consistent with the increase in DNA content; however, S1 and S2 only increased by 37 and 38%, respectively (Figures 1B,C, Table 2). Similarly, the cell volume of L1 and L2 increased by 86 and 98%, respectively, while S1 and S2 increased by 28 and 31%, respectively (Figures 1B,C, Table 2). Notably, cell and nuclear volume scaled proportionally with each other, such that the nucleus-to-cytoplasm (N/C) ratio of the respective parental cells was largely maintained in each 4N clone (Table 2). These findings show that tetraploidy has a variable effect on cell and nuclear volumes in DLD-1 cells and that doubling of the genome does not always result in doubling of nuclear size. Finally, we should note that in all 4N clones the majority of cells had normal centrosome numbers, consistent with a recent report that extra centrosomes are rapidly lost in 4N cells via asymmetric centrosome clustering during bipolar cell divisions and selective advantage of cells inheriting a single centrosome (Baudoin et al., 2020). During such bipolar divisions with asymmetric centrosome clustering, the chromosomes are partitioned in a bipolar manner and cytokinesis occurs at the midzone. Therefore, this process is unlikely to explain the size differences we observe in our clones, but it is consistent with the emergence of 4N cell populations in which the vast majority of cells assemble bipolar spindles with normal centrosome numbers (Ganem et al., 2009; Godinho et al., 2014; Kuznetsova et al., 2015; Potapova et al., 2016; Viganó et al., 2018; Baudoin et al., 2020).

**Table 2:** Characterization of parental DLD-1 and RPE-1 cells and derived tetraploid clones.

**Characteristic**	**DLD-1**	**S1**	**S2**	**L1**	**L2**	**RPE-1 (2N)**	**RPE-1 (4N)**
Modal Chromosome No.	46	91	92	88	91	46	88
Nuclear Volume^*^ (μm^3^)	847 ± 39	1,156 ± 41	1,170 ± 60	1,656 ± 107	1,648 ± 117	649 ± 27	1,465 ± 47
Cell Volume^*^ (μm^3^)	1,787 ± 80	2,288 ± 97	2,345 ± 123	3,326 ± 233	3,541 ± 244	2,403 ± 95	5,731 ± 270
N/C Ratio	0.48	0.51	0.50	0.51	0.47	0.27	0.27

* *Nuclear and cell volumes are reported as mean ± SEM*.

### Tetraploid Clones Display Distinct Mitotic Spindle and Cell Geometries

**Figure 2:**
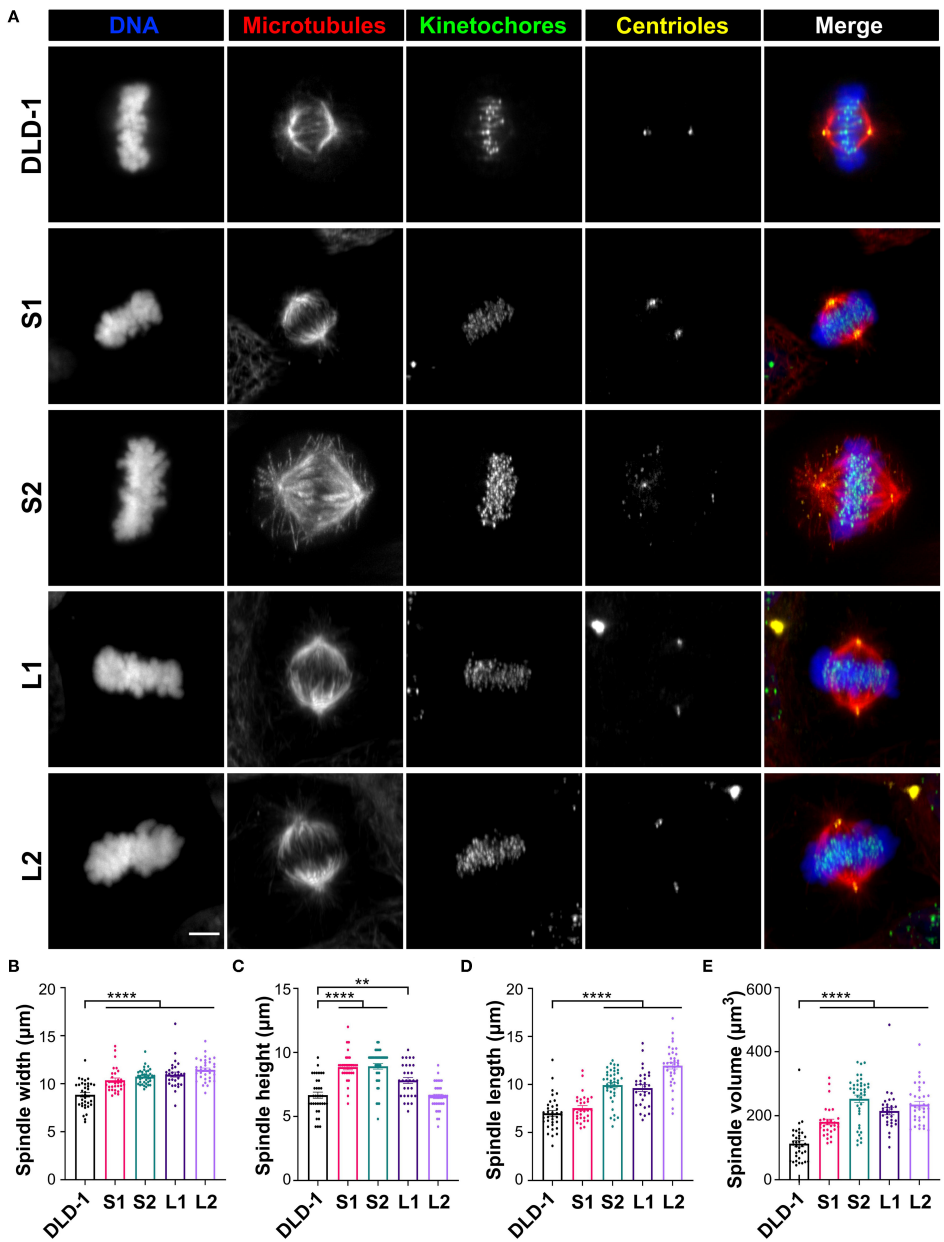
Tetraploid DLD-1 clones have distinct mitotic spindle geometries. **(A)** Examples of mitotic spindles from parental DLD-1 cells and cells from 4N DLD-1 clones S1, S2, L1, and L2 (top-to-bottom) in metaphase. Scale bar, 5 μm. Measurements of mitotic spindle width **(B)**, height **(C)**, length **(D)**, and volume **(E)** reported as mean ± SEM with individual data points from three independent experiments in which a total of 30–40 cells (*n* = 35, 30, 40, 30, and 35, respectively). **p < 0.01, ****p < 0.0001, when compared to the parental DLD-1 cells by Student's *t*-test.

Spindle length increases linearly with cell diameter in many metazoan species (Crowder et al., 2015), but in 4N budding yeast, spindle length did not increase compared to smaller diploid and haploid counterparts (Storchová et al., 2006). To determine if the mitotic spindle scales with cell size in our 4N clones, we measured spindle length, width, and height in metaphase cells with immunostained microtubules (α-tubulin), kinetochores (centromere antigen), and centrioles (centrin) (Figure 2A). Spindle width, measured as the distance between the outermost kinetochores at the metaphase plate, increased by roughly 20% in all the RPE-1 and DLD-1 4N clones compared to their respective parental cells (Figure 2B, Supplementary Figure 1C), suggesting that this width may be optimal for tetraploid cells regardless of their size and may be necessary to accommodate the extra chromosomes. Spindle height, which was also measured using kinetochores at the metaphase plate, increased by 25% in the RPE-1 4N clone compared to the RPE-1 diploid cells (Supplementary Figure 1D). In the 4N DLD-1 clones, however, spindle height did not significantly change in L2 and increased by only 17% in L1 compared to the parental cells (Figure 2C). Instead, in both small DLD-1 4N clones (S1 and S2), spindle height increased by 33–34% compared to the DLD-1 parental cells (Figure 2C). Finally, we measured spindle length as the distance between the spindle poles (defined by centriole staining) and found that it did not increase in the RPE-1 4N clone compared to its diploid ancestor (Supplementary Figure 1E), despite the considerable increase in cell volume (Supplementary Figure 1B). Compared to the diploid DLD-1 cells, spindle length increased by 37 and 71%, respectively, in the two large clones L1 and L2 (Figure 2D). In the two small clones, S1 and S2, spindle length increased by 7.7 and 42%, respectively (Figure 2D), indicating that spindle length can vary widely between tetraploid cells of similar sizes. These findings show that tetraploidy can have mixed effects on spindle length and height, producing distinct spindle geometries. The RPE-1 4N clone increased spindle width and height, but not spindle length, relative to the parental cells. Compared to diploid DLD-1, L1 and L2 primarily expanded spindle length, whereas S2 expanded both spindle height and spindle length, and S1 expanded spindle height, but not spindle length, resulting in a spindle with a “compressed” appearance (Supplementary Videos 1–10).

To determine if spindle volume correlates with cell volume, we calculated the volume of the entire mitotic spindle using the spindle dimensions of each 4N clone. In the RPE-1 4N clone, spindle volume did not scale with cell volume, increasing by only 60% relative to the RPE-1 cells (Supplementary Figure 1F). For the DLD-1 clones, spindle volume increased by 91 and 109% in L1 and L2, respectively (Figure 2E), compared to the parental DLD-1 cells, similar to the increases in cell volume (Figures 1B,C). In the small 4N DLD-1 clones, however, spindle volume scaling exceeded the changes in cell volume, with S1 and S2 increasing by 61 and 125%, respectively (Figure 2E), compared to parental DLD-1 cells. Moreover, despite being similar in size (Figures 1B–D), S1 and S2 had the smallest and largest spindle, respectively, of all the 4N DLD-1 clones (Figure 2E), suggesting that cell size is not the only determinant of spindle volume. Overall, these results show that in response to tetraploidy, spindle volume did not fully scale with cell size in the tetraploid RPE-1 cells, whereas in DLD-1 cells, although the 4N clones adjusted their spindle geometries differently, spindle volume mirrored or surpassed the increases in cell volume.

Since the large 4N DLD-1 clones mostly expanded spindle length and width rather than height, we asked whether there were also differences in the sphericity of metaphase cells in the 4N DLD-1 clones. Therefore, we measured the cell diameter (along the axis corresponding to spindle length) of metaphase cells. Cell diameter increased in L1 and L2 by about 25% compared to the parental DLD-1 cells; however, cell diameter did not change in S2 and decreased by 20% in S1 compared to the DLD-1 cells (Supplementary Figure 2A). To assess sphericity, we used the G2 cell volume measurements (from Figure 1C) to calculate the expected diameter assuming the cells formed perfect spheres during mitosis and compared this to our observed metaphase cell diameters (Supplementary Figure 2B). This showed that the observed and expected cell diameters were more similar in the small 4N DLD-1 clones compared to the large 4N DLD-1 clones or the DLD-1 cells (Supplementary Figure 2B), indicating that S1 and S2 cells are more spherical while L1 and L2 cells are more ellipsoid during mitosis. In particular, the observed and expected cell diameters in S1 were nearly identical (Supplementary Figure 2B), suggesting these cells form almost perfect spheres when dividing. Overall, these findings show a clear correlation between the size of tetraploid DLD-1 cells and the degree of mitotic rounding.

### Small Tetraploid Cells Spend More Time in Mitosis Due to a Delay in Metaphase

**Figure 3:**
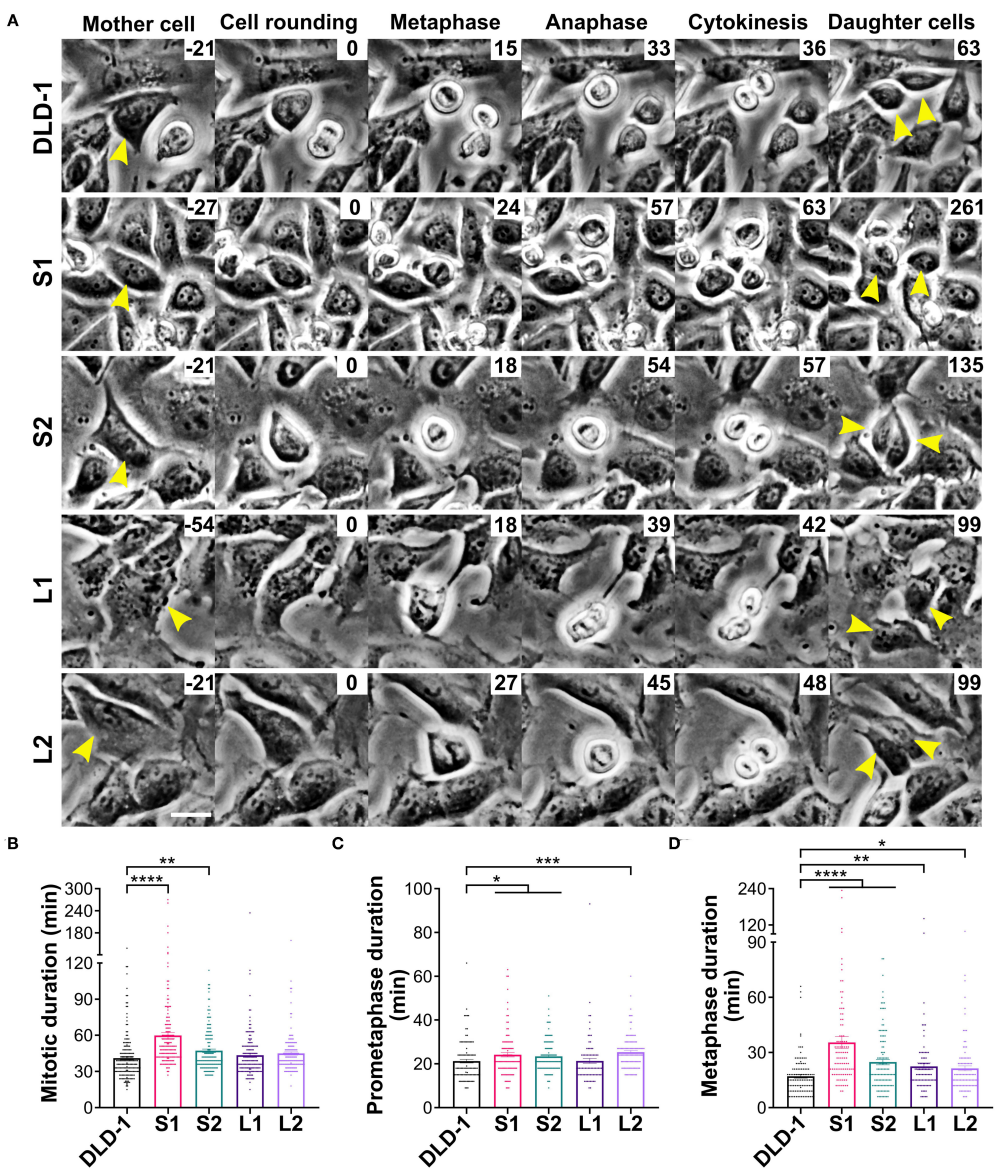
Small 4N DLD-1 cells spend more time in mitosis due to a delay in metaphase. **(A)** Examples of parental DLD-1 cells and cells from 4N DLD-1 clones S1, S2, L1, and L2 (top-to-bottom) progressing through mitosis. Elapsed time is shown in minutes (top-right corner), with 0 min corresponding to cell rounding. Yellow arrowheads point to the dividing cell (Mother cell column) and to the two daughter cells (right column). Scale bar, 25 μm. Quantification of **(B)** mitotic duration (*n* = 179, 167, 161, 161, and 150 cells, respectively), **(C)** prometaphase duration (*n* = 111, 101, 118, 90, and 110 cells, respectively), and **(D)** metaphase duration (*n* = 111, 101, 121, 91, and 101 cells, respectively). *p < 0.05, **p < 0.01, ***p < 0.001, and ****p < 0.0001, when compared to the parental DLD-1 cells by Student's *t*-test. Graphs in panels **(B–D)** represent data collected from three independent experiments reported as mean ± SEM with individual data points.

Previous studies have shown that, in some experimental systems, tetraploidy can lengthen mitosis, but it is unclear if cell and spindle scaling contribute to this delay (Kuznetsova et al., 2015; Viganó et al., 2018; Paim and FitzHarris, 2019; Cohen-Sharir et al., 2020; Quinton et al., 2020). To investigate whether the differences in cell size and spindle geometry among the 4N clones affect their progression through mitosis, we measured the timing from cell rounding to anaphase onset (referred to as mitotic duration henceforth) using live-cell phase contrast microscopy (Figure 3A and Supplementary Videos 11–15). Mitotic duration was similar in the 2N and 4N RPE-1 cells (Supplementary Figure 3A). Similarly, mitotic duration did not significantly change in L1 or L2 (43.5 and 45.0 min, respectively), but it was significantly longer in the small 4N clones S1 (59.8 min) and S2 (47.2 min) compared to DLD-1 cells (41.1 min; Figure 3B).

**Figure 4:**
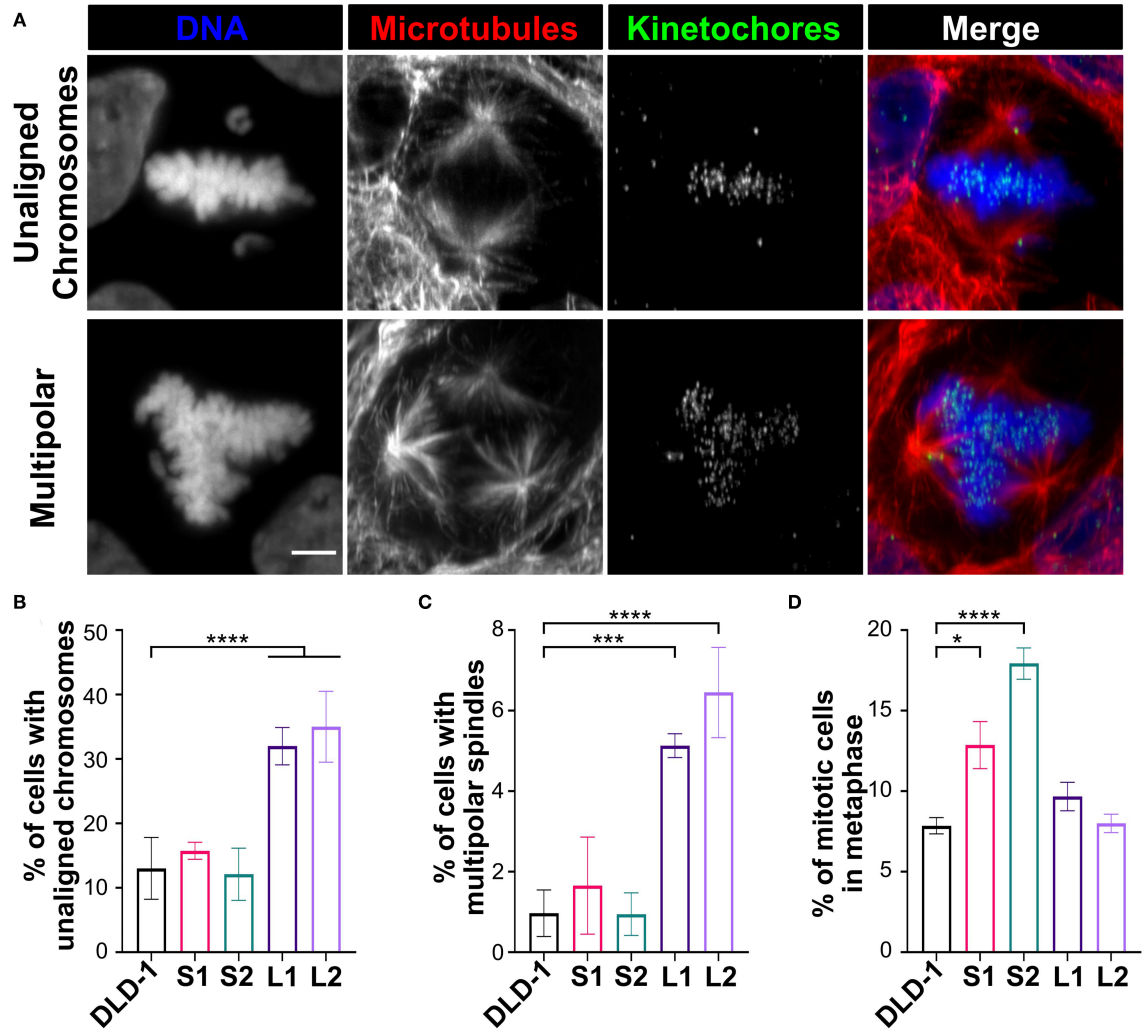
Defects in prometaphase do not cause the mitotic delay in small 4N DLD-1 clones. **(A)** Examples of a cell with unaligned chromosomes (top) and a multipolar spindle (bottom). Scale bar, 5 μm. **(B–D)** Quantification of the percentage of late prometaphase-metaphase cells with **(B)** unaligned chromosome(s) and **(C)** multipolar spindles. **(D)** Quantification of the percentage of mitotic cells in metaphase. Graphs for panels **(B–D)** represent the mean ± SEM of three independent experiments where at least 100 cells were scored in each experiment (*n* > 300 for each cell line). *p < 0.05, ***p < 0.001, ****p < 0.0001, when compared to parental DLD-1 cells using a two-sided Fisher's exact test.

Since the small 4N DLD-1 clones showed a delay in mitosis, we next sought to identify the underlying cause of the delay. Chromosome misalignment can delay mitosis (Potapova and Gorbsky, 2017), so we performed fixed-cell analysis (Figure 4A, top row) to quantify unaligned chromosome(s) in late prometaphase cells (i.e., cells with a well-defined metaphase plate and only 1 or a few unaligned chromosomes). Among all the 4N clones, L1, L2, and the 4N RPE-1, but not S1 and S2, displayed significantly higher frequencies of cells with unaligned chromosomes compared to the parental cells (Figure 4B and Supplementary Figure 3D), indicating that the mitotic delay in S1 and S2 is not caused by a delay in chromosome alignment. Transient spindle multipolarity (Figure 4A, bottom row) can also lengthen mitosis (Silkworth et al., 2009). However, L1, L2, and 4N RPE-1, but not S1 and S2, displayed an increase in multipolar prometaphase/metaphase cells compared to the parental cells (Figure 4C and Supplementary Figure 3E), indicating that transient multipolarity cannot explain the mitotic delay in the small 4N clones.

Overall, the above findings suggest the mitotic delay in small 4N DLD-1 clones may occur during metaphase. To explore this possibility, we used fixed-cell mitotic stage analysis to quantify the percentage of mitotic cells in metaphase (Figure 4D). We found similar percentages of metaphase cells in 4N RPE-1, L1, and L2 cells compared to their parental cells (Figure 4D and Supplementary Figure 3F). Conversely, the fraction of metaphase cells was significantly higher in S1 and S2 compared to the parental cells, suggesting that the delay in mitotic duration in the small 4N DLD-1 clones could be due to prolonged time in metaphase.

To further assess whether metaphase duration was prolonged in S1 and S2, we measured the time from cell rounding to metaphase (prometaphase) and from metaphase to anaphase onset (metaphase) by live-cell phase contrast microscopy. Consistent with the similar overall mitotic timing, 2N and 4N RPE-1 cells had comparable prometaphase durations of nearly 15 min (Supplementary Figure 3B). The parental DLD-1 cells had an average prometaphase duration of 21.2 min, which was similar to the prometaphase duration for L1 (21.4 min; Figure 3C). A modest increase in prometaphase duration was observed for all other 4N DLD-1 clones, where prometaphase duration was 23.4 (S2), 24.2 (S1), and 25.4 (L2) min (Figure 3C). However, these differences are not sufficient to explain the overall increase in mitotic duration in S1 and S2. When we examined metaphase duration, as expected, there was no significant difference between the 2N and 4N RPE-1 cells (Supplementary Figure 3C), whereas in each of the 4N DLD-1 clones, we observed an increase in metaphase duration compared to the parental cells. Consistent with our fixed-cell data, metaphase duration was the longest in S1 and S2 (Figure 3D). Specifically, whereas average metaphase duration was 17.1 min in the parental DLD-1 cells, it increased to 24.8 min in S2 and 35.4 min in S1 (Figure 3D). Altogether, these findings indicate that the mitotic delay in S1 and S2 results from a prolonged metaphase.

### Differences in Spindle Dimensions and Cell Size Are Not Sufficient to Explain the Observed Differences in Metaphase Duration in DLD-1 Clones

**Figure 5:**
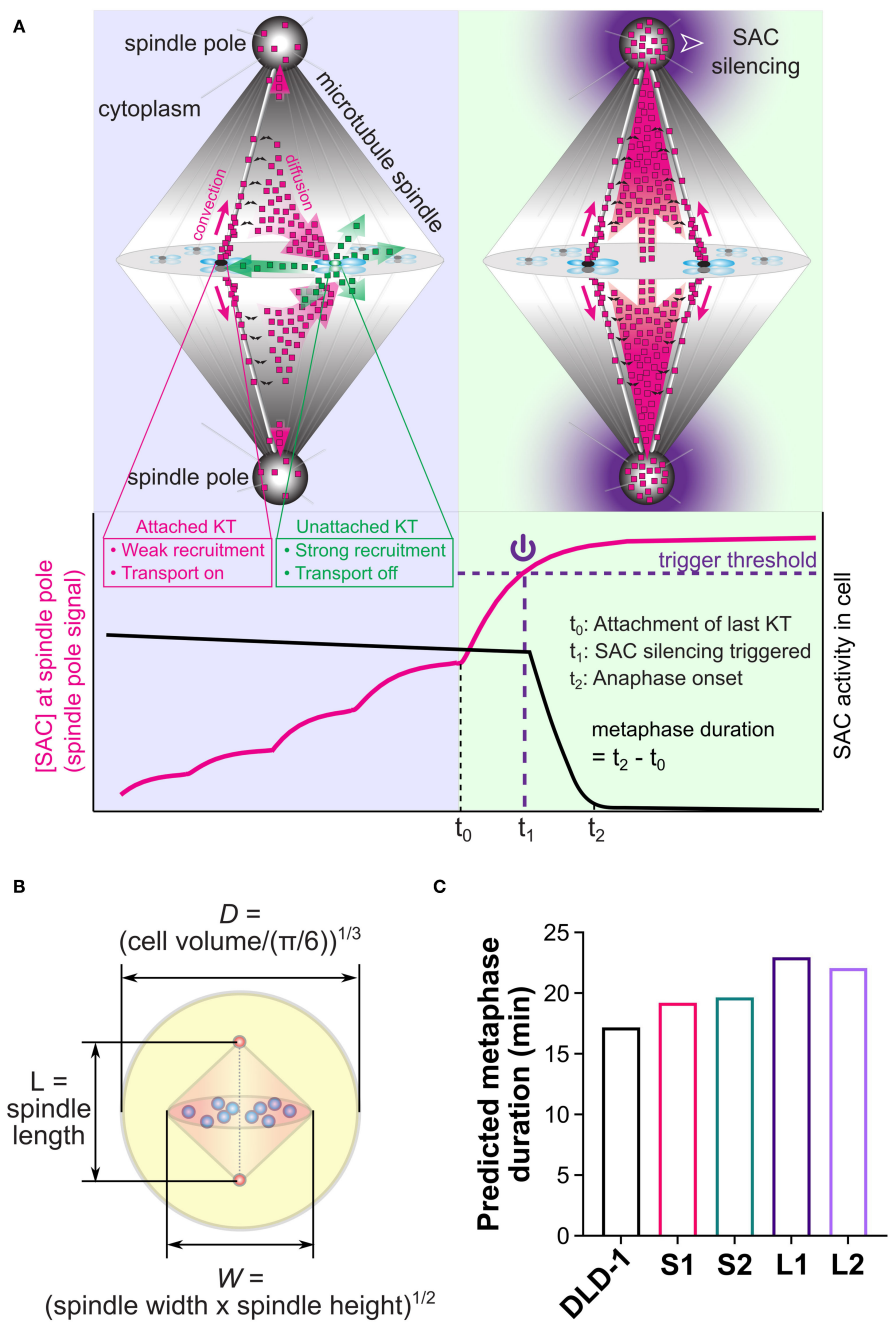
Mathematical model for spatiotemporal regulation of SAC silencing. **(A)** Cartoon summary of the model. Upper panels illustrate the key model assumptions: (a) High phosphorylation level at unattached kinetochores (e.g., right kinetochore in the upper left panel) promotes recruitment of SAC proteins and inhibits their transport activity (shedding transport-inactive SAC proteins illustrated by green dots); (b) Low phosphorylation level at attached kinetochores (e.g., left kinetochore in the upper left panel and all kinetochores in the upper right panel) causes much weaker recruitment of SAC proteins and turns on their transport activity (shedding transport-active SAC proteins illustrated by magenta dots); (c) Transport-active SAC proteins (magenta dots) partially accumulate at the spindle pole, and partially escape from the poleward transport by dissociation from the microtubules (black double arrows), getting the chance to return to the kinetochores. Lower panel illustrates the model predicted dynamics of the SAC protein concentration at the spindle pole (magenta line) and overall SAC activity in the cell (black line). Through the spatiotemporal dynamics illustrated in the cartoons, SAC proteins accumulate at the spindle pole in a nonlinear fashion that depends on successive kinetochore attachments. The last kinetochore attachment causes a substantial jump in this spindle pole signal, which is assumed to trigger SAC silencing when crossing a threshold level (purple dashed line). The metaphase duration is the sum of the “triggering time” required for the spindle pole signal to reach the trigger threshold (*t*_1_ − *t*_0_) and the “propagation time” required for SAC silencing to propagate from the spindle pole throughout the cell (*t*_2_ − *t*_1_). **(B)** Cell and spindle dimensions in the model are calculated from experimental measurements. **(C)** Model predicted metaphase durations for the parental DLD-1 cells and the 4N DLD-1 clones using the cell and spindle dimensions shown in panel **(B)**. Values of the dimensions are given in Table 1. Panels **(A,B)** adapted with permission from (Chen and Liu, 2016).

Metaphase duration depends on the time required to silence the SAC after the last chromosome aligns at the metaphase plate (Rieder et al., 1994, 1995; Howell et al., 2000), and spindle and cell sizes have been proposed to impact the dynamics of SAC silencing (Chen and Liu, 2014, 2015, 2016). Thus, it is possible that the longer metaphase duration in our small 4N DLD-1 clones may stem from a size-dependent change in the time required for SAC silencing. To assess this possibility in explaining the intriguing relationship between cell/spindle sizes and metaphase duration observed in the 4N DLD-1 clones, we took advantage of a previous mathematical model (Chen and Liu, 2014) that addresses the spindle-mediated spatiotemporal regulation of SAC signals and associates the dynamics of SAC silencing with cell and spindle sizes (Chen and Liu, 2014, 2015). The model was built upon the phenomenon that SAC proteins are concentrated at the unattached kinetochores, but undergo continuous dynein-mediated transport from the attached kinetochores to the spindle pole (Howell et al., 2000, 2001; Wojcik et al., 2001; Basto et al., 2004; Griffis et al., 2007; Silva et al., 2014) (Figure 5A) (see Materials and Methods for basic model assumptions and Supplementary Materials for details and equations). The model predicts that such spatiotemporal regulation can lead to a nonlinear increase in the concentration of SAC proteins at the spindle pole, as more kinetochores within the cell achieve stable attachment to spindle microtubules (Figure 5A). Particularly, attachment of the last kinetochore can induce a drastic increase in the SAC concentration at the spindle pole. Such a dramatic increase can generate a noise-robust signal to silence the SAC after and only after all kinetochores are attached, if silencing is triggered by a proper threshold concentration of SAC proteins at the spindle pole (Chen and Liu, 2014) (Figure 5A). Thus, the model suggests that dynein-mediated poleward transport of SAC proteins could function to ensure robust, timely SAC silencing (Chen and Liu, 2014). In the model, the trigger signal for SAC silencing at the spindle pole could be any specific SAC protein, or any additional mitotic signaling protein undergoing the same spatiotemporal regulation as the SAC proteins, including cyclin B and APC/C (Acquaviva et al., 2004; Torres et al., 2010; Famulski et al., 2011). In the rest of the paper, we will refer to this putative SAC silencing trigger as the “spindle pole signal.” Because the model treats the entire spindle, including kinetochores, microtubules and spindle poles, as an integrated mediator of the SAC silencing signal, it can predict how SAC silencing depends on spindle and cell sizes (Chen and Liu, 2016).

To assess the influence of size differences, we entered the median spindle and cell dimensions measured in the parental DLD-1 and 4N clones into the model (Table 1, Figure 5B), and calculated metaphase duration as the time delay between the last kinetochore attachment and SAC inactivation (Figure 5A, *t*_2_ − *t*_0_). We assumed the same values for the remaining model parameters across all cell clones, such as binding/unbinding constants and biochemical rate constants (Supplementary Tables 1, 2). We found that the spindle and cell dimensions alone cannot explain the observed differences in metaphase duration in the 4N DLD-1 clones (Figure 5C). Specifically, the model predicts the two small 4N DLD-1 clones, S1 and S2, to have shorter metaphase durations than the two large 4N DLD-1 clones, L1 and L2. This is inconsistent with our experimental observations that metaphase is longer in S1 and S2 compared to L1 and L2. Furthermore, it is inconsistent with our finding that cyclin B persists in a larger fraction of metaphase cells in the small 4N DLD-1 clones compared to L1, L2, and parental DLD-1 cells (Supplementary Figure 4). Because cyclin B is degraded prior to anaphase onset (Chang et al., 2003), its persistence is indicative of a delay in SAC silencing.

### Differences in Metaphase Duration Can Be Explained by Combined Differences in Cell Size, Spindle Size, Spindle Pole Size, and Spindle Microtubule Abundance

**Figure 6:**
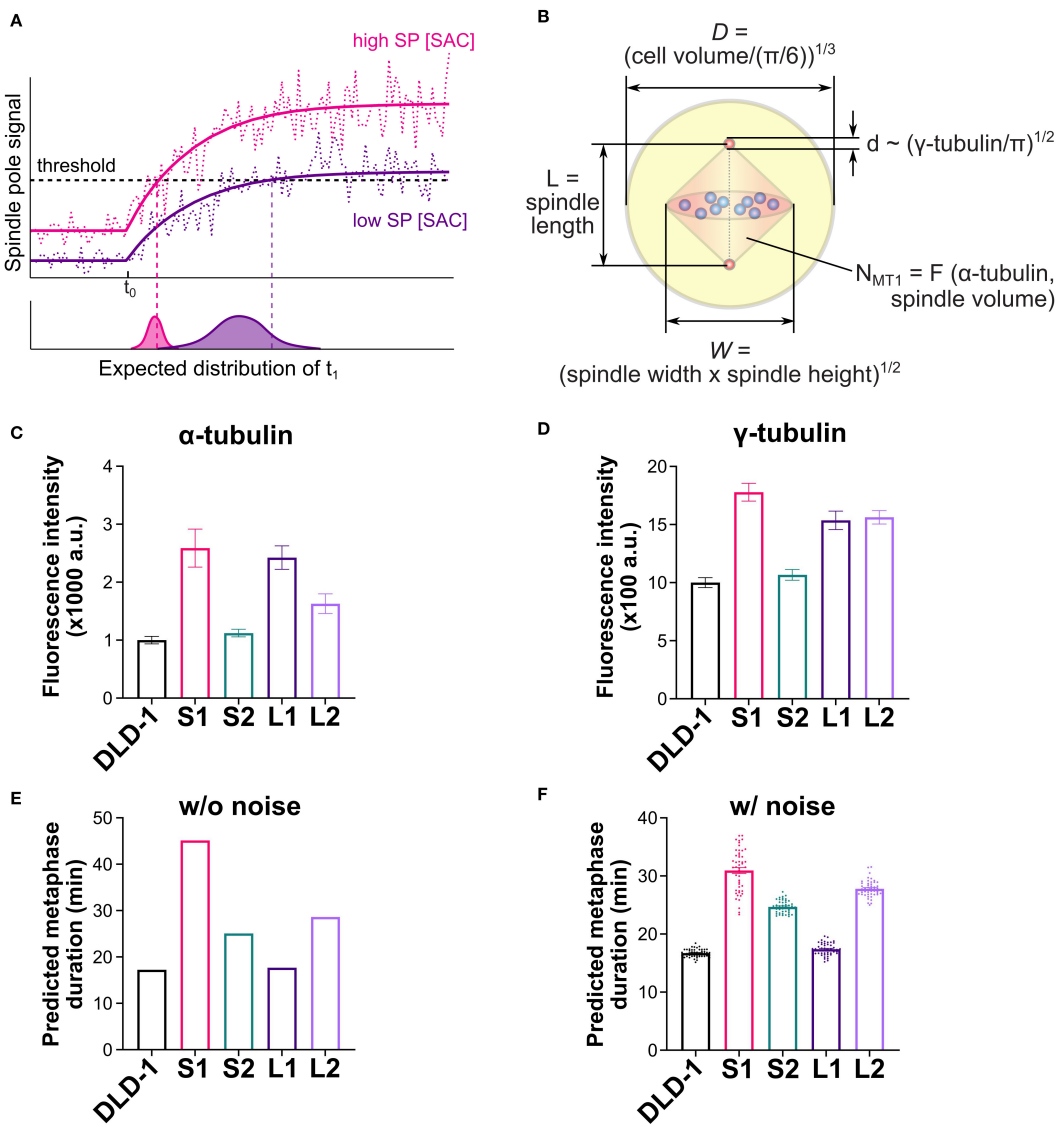
Mathematical model explains observed metaphase durations when combining cell/spindle dimensions with tubulin measurements. **(A)** Effects of spindle pole signal level on predicted metaphase duration. A higher overall level of the spindle pole signal (magenta line) is expected to trigger SAC silencing earlier than a lower level does (purple line). Noise (dashed lines) is expected to expedite triggering of SAC silencing and cause variation in the triggering time, and both effects are expected to be more significant with lower spindle pole signal level. **(B)** Dimensions of cell, spindle and spindle pole, and abundance of microtubules calculated from experimental measurements (refer to the Supplementary Materials for the derivation of microtubule number, *N*_*MT*1_). **(C)** Experimentally measured α-tubulin intensity in the parental DLD-1 cells and the 4N DLD-1 clones. **(D)** Experimentally measured γ-tubulin intensity in the parental DLD-1 cells and the 4N DLD-1 clones. **(E,F)** Metaphase durations predicted for the parental DLD-1 cells and the 4N DLD-1 clones using the dimensions shown in panel **(B)** without **(E)** and with **(F)** noise. Values of the dimensions are given in Table 1. Scatter dots in panel **(F)** show 50 individual simulation results and bars show the average.

We next considered additional factors that can influence the dynamics of the spindle pole signal and consequently the predicted time for SAC silencing. The predicted metaphase duration reflects the sum of the “triggering time” and the “propagation time.” The triggering time (Figure 5A, *t*_1_ − *t*_0_) is the time it takes the spindle pole signal to reach the trigger threshold after attachment of the last kinetochore. The triggering time also corresponds to the time period during which SAC silencing can be reversed by disrupting microtubule-kinetochore attachments (Dick and Gerlich, 2013). The propagation time (Figure 5A, *t*_2_ − *t*_1_) is the time it takes SAC silencing to propagate from the spindle pole throughout the cell. In the model, the propagation time largely depends on the biochemical pathway for SAC silencing, which does not differ significantly among clones derived from the same ancestor (Supplementary Figure 5), as they assume the same biochemical parameters. Instead, the difference in the predicted metaphase duration largely stems from the difference in the triggering time (Supplementary Figure 5), which heavily depends on the dynamics of the spindle pole signal, especially on how much the final spindle pole signal exceeds the threshold (Figure 6A). If the final spindle pole signal is very close to the threshold, it takes a considerably longer time for the signal to reach the threshold that is located near the plateau of the temporal curve (Figure 6A, purple solid line). Therefore, any factor that significantly impacts the spindle pole signal is expected to affect metaphase duration.

Aside from the spindle and cell dimensions, spindle microtubule abundance and spindle pole size can also strongly affect the spindle pole signal (Supplementary Figure 6) and therefore influence the triggering time. To obtain information on spindle microtubule abundance and spindle pole size, we quantified the fluorescence intensity of α- and γ-tubulin, respectively (Figures 6C,D and Supplementary Figures S7A,B). To obtain model parameters from these data, we converted the median α-tubulin signal intensity measured in each clone to the abundance of spindle microtubules in the model (Figure 6B, *N*_*MT*1_) [Table 1; Supplementary Materials, Eq. (S2)]. We also took the median γ-tubulin signal intensity as a metric for spindle pole size and used it to derive the spindle pole diameters relative to the parental cells (Figure 6B, *d*) [Table 1; Supplementary Materials, Eq. (S1)]. When spindle microtubule abundance and spindle pole size were included, the model largely captured the experimentally observed relative metaphase durations in different DLD-1 clones, especially the observation that metaphase duration is much longer in S1 than all other clones (Figure 6E). The metaphase durations predicted for parental and 4N RPE-1 clones also matched the experimental data (Supplementary Figure 7C).

We further examined the variations in the predicted metaphase duration due to noise. When random noise in the spindle pole signal is considered (Figure 6A, dotted lines), SAC silencing has a chance to be triggered before the average signal reaches the trigger threshold. For cases with a high overall level of spindle pole signal, the threshold is located in the fast-rising region of the spindle pole signal and hence the triggering time is dominated by the rising dynamics and relatively smaller variation is expected (Figure 6A, lower plot, magenta distribution). For cases with a low overall level of spindle pole signal, as the signal slowly approaches the threshold in the near-plateau region, noise plays a much more important role, resulting in shorter and more stochastic triggering time (Figure 6A, lower plot, purple distribution). Indeed, when 20% noise was applied to the spindle pole signal in the model (see Supplementary Materials), the predicted metaphase duration in S1 was significantly lowered from 45 min in the deterministic simulation to 31 min average in the stochastic simulations with a wide distribution (Figure 6F). In contrast, the predicted metaphase durations for the parental DLD-1 cells and other 4N DLD-1 clones displayed average values close to the corresponding deterministic results and narrower distributions (Figure 6F). This is consistent with the experimental observation that metaphase duration in S1 was more stochastic (broader distribution) than in the parental cells and other 4N DLD-1 clones (Figure 3D). The parental and 4N RPE-1 clones are also predicted to have similar average metaphase duration as their corresponding deterministic predictions (Supplementary Figures 7C,D). In sum, our model shows that the difference in metaphase duration among the 4N clones can stem from the combined variations in cell size, spindle size, spindle pole size, and spindle microtubule abundance.

### Changes in Spindle Shape Can Affect the Timing of SAC Silencing

**Figure 7:**
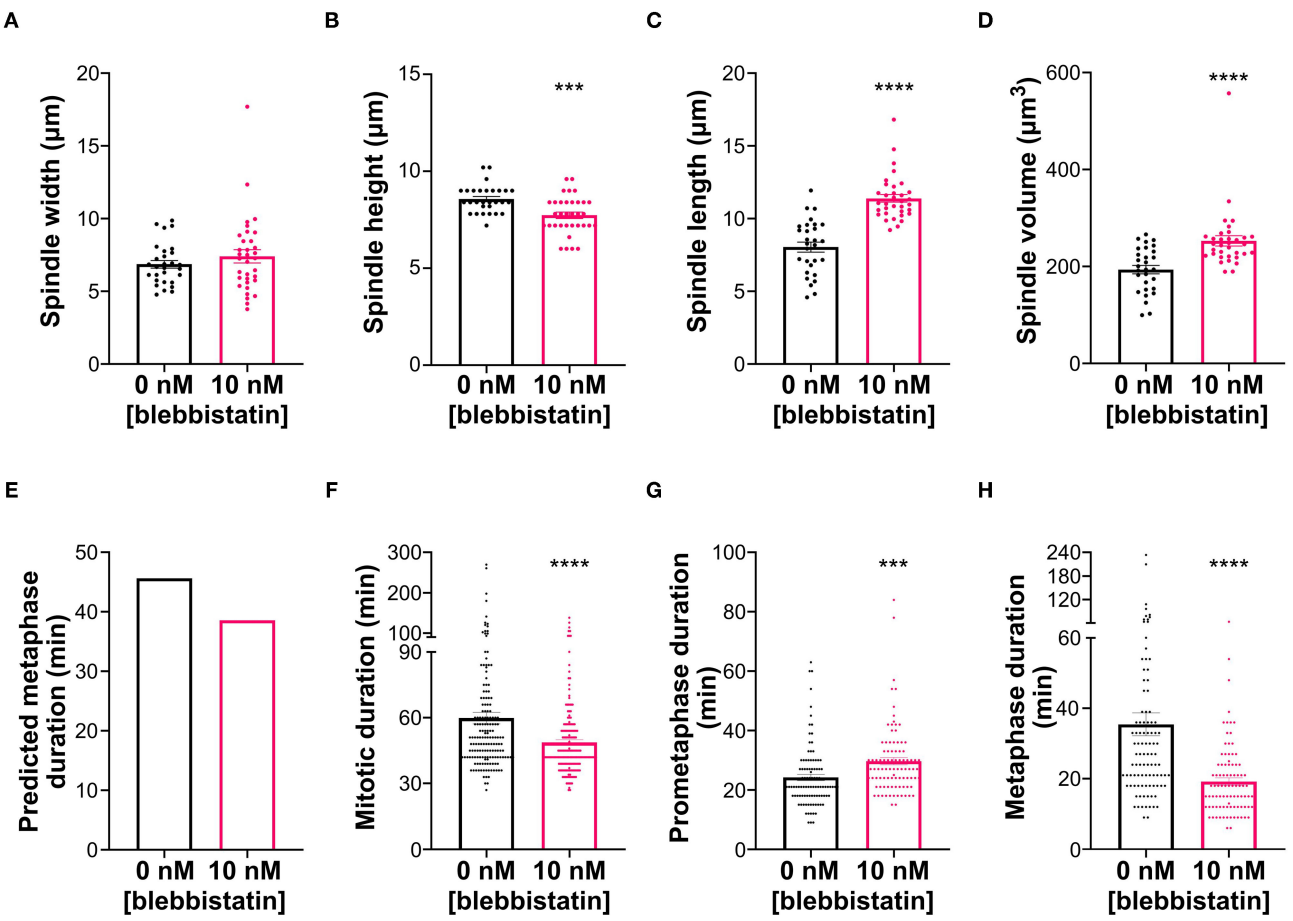
Blebbistatin treatment modifies spindle dimensions and reduces metaphase duration in S1 cells. **(A–D)** Measurements of mitotic spindle width **(A)**, height **(B)**, length **(C)**, and volume **(D)** in untreated and blebbistatin-treated S1 cells (*n* = 29 and 36, respectively), reported as mean ± SEM with individual data points from three independent experiments. ***p < 0.001, ****p < 0.0001, when compared to untreated cells by Student's *t*-test. **(E)** Model-predicted metaphase duration using the median spindle dimensions in panels **(A–C)**. Cell size, spindle pole size, and spindle microtubule abundance were assumed to be the same between the control and treated groups. **(F–H)** Quantification of **(F)** mitotic duration in untreated and blebbistatin-treated S1 cells (*n* = 167 and 193, respectively), **(G)** prometaphase duration in untreated and blebbistatin-treated S1 cells (*n* = 101 and 92, respectively), and **(H)** metaphase duration in untreated and blebbistatin-treated S1 cells (*n* = 101 and 94, respectively). ***p < 0.001, ****p < 0.0001, when compared to untreated cells by Student's *t*-test.

In addition to displaying the longest metaphase duration, the S1 clone also displayed a unique spindle morphology, which we described in an earlier section as “compressed,” given the substantial increase in spindle height, but not in spindle length, compared to the parental cells. We aimed to address whether this spindle morphology may contribute to the metaphase delay in these cells. Because S1 cells form nearly spherical mitotic cells, we reasoned that we may be able to alter spindle shape by limiting mitotic cell rounding. To this end, we treated S1 cells with the myosin inhibitor blebbistatin, since actomyosin contraction is important for mitotic cell rounding and spindle morphogenesis (Kunda et al., 2008; Kunda and Baum, 2009). Indeed, spindle size measurements (Figures 7A–D) showed that, at a low dose, blebbistatin treatment did not affect spindle width (Figure 7A) of S1 cells, but reduced spindle height by 10% and increased spindle length by 42% (Figures 7B,C) compared to untreated S1 cells. Using these size measurements in the model, we found that these changes in spindle dimensions decreased the predicted metaphase duration by 7 min (or 15%) (Figure 7E). To experimentally test this prediction, we quantified mitotic timing by time-lapse microscopy (Figures 7F–H) and found that mitotic duration was 11 min (or 18.6%) shorter in blebbistatin-treated S1 cells compared to untreated S1 cells (Figure 7F). Importantly, the decrease in mitotic duration could be explained by a significantly shorter metaphase duration (Figure 7H), in line with our model prediction. Altogether, these findings suggest that spindle shape plays an important role in timely SAC silencing and mitotic progression.

## Discussion

In this study, we generated tetraploid cell lines from RPE-1 and DLD-1 cells to examine the relationship between cell and spindle size scaling and mitotic progression. Our 4N DLD-1 clones could be separated into two groups (small and large) based on cell and nuclear volumes, supporting the notion that genome size does not necessarily set cell/nuclear size (Neumann and Nurse, 2007). This is also consistent with the observation that cells become progressively smaller during development, despite maintaining a constant genome size (Hara and Kimura, 2009). In contrast, we were not able to isolate a small 4N RPE-1 clone. The ability to suppress cell/nuclear size scaling in response to changes in DNA content may be specific to cancer cells and could have relevance for tumorigenesis. Indeed, DNA content and nuclear size do not always correlate in cancers (Wang et al., 1992). The mechanisms responsible for regulating and altering cell/nuclear size in normal and cancer cells warrant further study. Regardless of how much cell and nuclear sizes scaled, the N/C ratio was maintained in each cell type, which is consistent with many previous studies in which cell and nuclear sizes were examined in a variety of contexts (Conklin, 1912; Jorgensen et al., 2007; Neumann and Nurse, 2007).

### Spindle Size Does Not Always Scale With Cell Size

Across many metazoan species and within developing organisms, spindle size typically scales with cell size, such that larger cells have longer spindles and vice versa (Brown et al., 2007; Loughlin et al., 2010, 2011; Good et al., 2013; Hazel et al., 2013; Reber et al., 2013; Wilbur and Heald, 2013; Crowder et al., 2015; Lacroix et al., 2018). Moreover, *Xenopus* egg extract spindles assembled in microfluidic devices were shown to adjust their size based on the available space (Good et al., 2013; Hazel et al., 2013). Our results, however, show that spindle geometry or size do not show an obvious correlation with cell size and that the relation between cell and spindle size is more complex than traditionally believed. Similar to a study in yeast (Storchová et al., 2006), spindle length did not increase with cell volume in the RPE-1 4N clone. Spindle width was similar in all of the 4N clones, despite differences in cell and spindle volumes, suggesting that the increase in chromosome number following tetraploidization may require a specific adjustment of spindle width. Moreover, width may only increase within certain constraints, given that excessively wide spindles can result in pole splitting and multipolarity (Dinarina et al., 2009; Lancaster et al., 2013). The lack of correlation between cell and spindle size was particularly evident in the small 4N DLD-1 clones, S1 and S2. Despite having similar cell and nuclear volumes, S1 and S2 displayed the smallest and largest spindle volumes, respectively, out of all the 4N DLD-1 clones, indicating that factors other than cell volume can determine spindle volume.

Of all the spindle dimensions, spindle height appeared to be critical for adjusting spindle volume in response to cell size variations. Indeed, the small 4N DLD-1 clones were characterized by spindles that were taller than the spindles in the large 4N clones. We found that inhibiting myosin with blebbistatin reduced spindle height in S1, indicating that actomyosin contraction during mitotic cell rounding assists in vertical expansion of the spindle. During mitotic cell rounding, which facilitates proper spindle formation (Kunda et al., 2008; Kunda and Baum, 2009), actomyosin contraction helps to counterbalance the outward force generated by osmotic pressure (Stewart et al., 2011). Since osmosis depends on the movement of ions and water across the cell membrane (Lang, 2007), the increased surface area-to-volume ratio of the small 4N DLD-1 clones may enable these cells to more readily regulate the osmotic gradients needed for mitotic rounding compared to the large 4N DLD-1 clones. Indeed, we found that mitotic cell rounding was most pronounced in S1 and S2, indicating that the efficiency of mitotic cell rounding may explain the relationship between cell size and spindle height in tetraploid cells. Mitotic rounding and cytoskeletal contraction were also shown to contribute to efficient mitotic spindle assembly and chromosome alignment (Lénárt et al., 2005; Lancaster et al., 2013; Booth et al., 2019), which can explain our observation that the small 4N clones had fewer defects in chromosome alignment compared to the large 4N clones. Thus, our findings suggest that the size of tetraploid cells can influence several aspects of mitosis, including mitotic rounding, spindle morphogenesis, and chromosome alignment.

### Cell and Spindle Sizes Are Not the Only Determinants of SAC Silencing Timing

Previous studies have shown that SAC “strength” and mitotic duration increase as cell size decreases (Galli and Morgan, 2016; Gerhold et al., 2018). This is consistent with our observation that the small 4N clones displayed the longest mitotic durations among DLD-1 clones. Even with similar cell volumes, however, S1 and S2 had different mitotic and metaphase durations, indicating that cell size is not sufficient to explain the timing of SAC silencing. The spatiotemporal model for SAC silencing (Chen and Liu, 2014) we used here provides a theoretical framework to examine the combined effects of multiple parameters on SAC silencing time. For instance, among the cell and spindle dimensions, only spindle length differed significantly between S1 and S2, but the model predicts that the spindle pole signal is rather insensitive to spindle length (Chen and Liu, 2016). Hence, the difference in metaphase duration between S1 and S2 cannot be explained by differences in cell or spindle dimensions. However, the two small clones displayed particularly pronounced differences in their α- and γ-tubulin intensities, which translated to significantly higher spindle microtubule density and larger spindle poles in S1. According to the model, higher spindle microtubule density leads to stronger poleward flux of the SAC proteins, which increases the spindle pole signal and shortens metaphase duration. Meanwhile, larger spindle poles dilute the spindle pole signal and lengthen metaphase duration. Between S1 and S2, the impact of spindle pole size outcompeted the impact of spindle microtubule density, leading to a longer metaphase duration in S1. Unlike the comparison between S1 and S2, S1 and L1 displayed similar α- and γ-tubulin intensities. However, they showed different cell sizes and different spindle heights. Spindle height is particularly interesting because it has been shown that limiting cell height, and hence spindle height, through chemical or physical perturbations impairs mitotic progression (Dumont and Mitchison, 2009; Lancaster et al., 2013; Cattin et al., 2015). Our model suggested that the difference in metaphase duration between S1 and L1 stems from the larger cell size and smaller spindle height in L1, both of which increase the spindle pole signal and shorten metaphase duration. Consistent with this, when spindle height was experimentally reduced in S1 cells, metaphase duration was also reduced. Therefore, our data show that not only limiting spindle height, but also excessive spindle height (as that observed in S1 cells) can affect mitotic progression. Overall, our mathematical model showed that, although spindle scaling is important for robust SAC signaling (Chen and Liu, 2014, 2016), spindle size alone is not sufficient to predict the kinetics of SAC silencing. Instead, our data show that timing of SAC silencing can only be explained by the combined contributions of cell, spindle and pole sizes along with microtubule abundance.

Although the model explains most of the differences in metaphase duration among the parental cells and 4N DLD-1 clones, certain discrepancies remain between the model predictions and the experimental data. For example, the predicted metaphase duration in L2 is higher than all the other DLD-1 clones except for S1, which does not match the experimental data. Such a discrepancy could stem from additional factors that might differ among the clones, such as biochemical or transport activity of SAC proteins, which will require future investigations. The discrepancy could also be attributed to incompleteness of the model. The model has made a number of assumptions that yet await experimental verification. For example, SAC silencing is assumed to be triggered by a threshold concentration of a “spindle pole signal” that arrives by the dynein-mediated poleward transport. Although the dynein-mediated transport has been documented (Howell et al., 2000, 2001; Wojcik et al., 2001; Basto et al., 2004; Griffis et al., 2007; Silva et al., 2014), the identity of the signal, existence of the threshold, and the downstream pathway triggered by the threshold all need to be determined. Moreover, the biochemical part of the model is coarse-grained, resolving only a few key molecular players in the SAC mechanism. Nevertheless, using identical parameters for the biochemical circuits and protein transport across all the clones derived from the same ancestor, the model predictions provide insight on the relative differences in SAC silencing time among these clones, which is not attainable by statistical comparison of sizes alone.

### Revisiting the Role of Tetraploidy and Spindle Architecture in Tumorigenesis

Tetraploidization has been proposed as an intermediate step during tumor progression (Storchova and Pellman, 2004; Ganem et al., 2007). This idea is supported by a number of observations, including the fact that nearly 40% of all tumors have likely undergone whole genome duplication during their clonal evolution (Zack et al., 2013) and that tetraploid, but not diploid, mammary epithelial cells could induce subcutaneous tumors in nude mice (Fujiwara et al., 2005). Newly formed tetraploid cells inherit extra centrosomes that promote spindle multipolarity (Chen et al., 2016; Baudoin et al., 2020), which was proposed as the mechanism underlying the tumorigenic potential of tetraploid cells (Storchova and Pellman, 2004). In our 4N clones, however, a majority of cells formed bipolar spindles with a normal centrosome number, consistent with other reports (Ganem et al., 2009; Godinho et al., 2014; Kuznetsova et al., 2015; Potapova et al., 2016; Viganó et al., 2018) and recent evidence that extra centrosomes are quickly lost through asymmetric centrosome clustering during the evolution of tetraploid cells (Baudoin et al., 2020). Our findings suggest that rather than multipolarity resulting from extra centrosomes, relatively subtle variations in spindle geometry, spindle pole size, microtubule abundance, and cell volume can affect mitotic progression and SAC silencing in tetraploid cells. Thus, even though spindle bipolarity may be preserved, other changes in cell size and spindle architecture—its geometry and composition—may promote mitotic dysfunction and contribute to the genomic instability caused by tetraploidy (Storchova and Kuffer, 2008; Dewhurst et al., 2014; Cohen-Sharir et al., 2020; Quinton et al., 2020).

## Data Availability Statement

The raw data supporting the conclusions of this article will be made available by the authors, without undue reservation.

## Ethics Statement

Studies involving animal subjects: no animal studies are presented in this manuscript. Studies involving human subjects: no human studies are presented in this manuscript. Inclusion of identifiable human data: no potentially identifiable human images or data is presented in this study.

## Author Contributions

MB performed all the experiments, data acquisition, and data analysis. JC performed all the modeling work. DC provided resources and reagents. All authors contributed to conception of the study, data interpretation, figure preparation, and writing of the manuscript.

## Conflict of Interest

The authors declare that the research was conducted in the absence of any commercial or financial relationships that could be construed as a potential conflict of interest.

## References

[B1] Acquaviva C. Herzog F. Kraft C. Pines J. (2004). The anaphase promoting complex/cyclosome is recruited to centromeres by the spindle assembly checkpoint. Nat. Cell Biol. 6, 892–882. 10.1038/ncb1167 15322556

[B2] Arnaoutov A. Azuma Y. Ribbeck K. Joseph J. Boyarchuk Y. Karpova T.. (2005). Crm1 is a mitotic effector of Ran-GTP in somatic cells. Nat. Cell Biol. 7, 626–632. 10.1038/ncb1263 15908946

[B3] Basto R. Scaerou F. Mische S. Wojcik E. Lefebvre C. Gomes R.. (2004). In vivo dynamics of the rough deal checkpoint protein during Drosophila mitosis. Curr. Biol. 14, 56–61. 10.1016/j.cub.2003.12.025 14711415

[B4] Baudoin N. C. Cimini D. (2018). A guide to classifying mitotic stages and mitotic defects in fixed cells. Chromosoma 127, 215–227. 10.1007/s00412-018-0660-2 29411093

[B5] Baudoin N. C. Nicholson J. M. Soto K. Martin O. Chen J. Cimini D. (2020). Asymmetric clustering of centrosomes defines the early evolution of tetraploid cells. Elife 9:e54565. 10.7554/eLife.54565.sa2 32347795 PMC7250578

[B6] Bolte S. Cordelières F. P. (2006). A guided tour into subcellular colocalization analysis in light microscopy. J. Microsc. 224, 213–232. 10.1111/j.1365-2818.2006.01706.x 17210054

[B7] Booth A. J. Yue Z. Eykelenboom J. K. Stiff T. Luxton G. G. Hochegger H.. (2019). Contractile acto-myosin network on nuclear envelope remnants positions human chromosomes for mitosis. Elife 8:e46902. 10.7554/eLife.46902.040 31264963 PMC6634967

[B8] Brewer B. J. Chlebowicz-Sledziewska E. Fangman W. L. (1984). Cell cycle phases in the unequal mother/daughter cell cycles of Saccharomyces cerevisiae. Mol. Cell. Biol. 4, 2529–2531. 10.1128/MCB.4.11.2529 6392855 PMC369084

[B9] Brown K. S. Blower M. D. Maresca T. J. Grammer T. C. Harland R. M. Heald R. (2007). Xenopus tropicalis egg extracts provide insight into scaling of the mitotic spindle. J. Cell Biol. 176, 765–770. 10.1083/jcb.200610043 17339377 PMC2064050

[B10] Cattin C. J. Düggelin M. Martinez-Martin D. Gerber C. Müller D. J. Stewart M. P. (2015). Mechanical control of mitotic progression in single animal cells. Proc. Natl. Acad. Sci. U. S. A. 112, 11258–11263. 10.1073/pnas.1502029112 26305930 PMC4568679

[B11] Chang D. C. Xu N. Luo K. Q. (2003). Degradation of cyclin B is required for the onset of anaphase in Mammalian cells. J. Biol. Chem. 278, 37865–37873. 10.1074/jbc.M306376200 12865421

[B12] Chen J. Liu J. (2014). Spatial-temporal model for silencing of the mitotic spindle assembly checkpoint. Nat. Commun. 5:4795. 10.1038/ncomms5795 25216458 PMC4163959

[B13] Chen J. Liu J. (2015). Erroneous silencing of the mitotic checkpoint by aberrant spindle pole-kinetochore coordination. Biophys. J. 109, 2418–2435. 10.1016/j.bpj.2015.10.024 26636952 PMC4675864

[B14] Chen J. Liu J. (2016). Spindle size scaling contributes to robust silencing of mitotic spindle assembly checkpoint. Biophys. J. 111, 1064–1077. 10.1016/j.bpj.2016.07.039 27602734 PMC5018141

[B15] Chen S. Stout J. R. Dharmaiah S. Yde S. Calvi B. R. Walczak C. E. (2016). Transient endoreplication down-regulates the kinesin-14 HSET and contributes to genomic instability. Mol. Biol. Cell 27, 2911–2923. 10.1091/mbc.E16-03-0159 27489338 PMC5042578

[B16] Clute P. Pines J. (1999). Temporal and spatial control of cyclin B1 destruction in metaphase. Nat. Cell Biol. 1, 82–87. 10.1038/10049 10559878

[B17] Cohen-Sharir Y. McFarland J. M. Abdusamad M. Marquis C. Tang H. Ippolito M. R. (2020). Selective vulnerability of aneuploid human cancer cells to inhibition of the spindle assembly checkpoint. bioRxiv [Preprint]. 10.1101/2020.06.18.159038

[B18] Conklin E. G. (1912). Cell size and nuclear size. J. Exp. Zool. 12, 1–98. 10.1002/jez.1400120102

[B19] Crowder M. E. Strzelecka M. Wilbur J. D. Good M. C. von Dassow G. Heald R. (2015). A comparative analysis of spindle morphometrics across metazoans. Curr. Biol. 25, 1542–1550. 10.1016/j.cub.2015.04.036 26004761 PMC4464779

[B20] Dewhurst S. M. McGranahan N. Burrell R. A. Rowan A. J. Grönroos E. Endesfelder D.. (2014). Tolerance of whole-genome doubling propagates chromosomal instability and accelerates cancer genome evolution. Cancer Dis. 4, 175–185. 10.1158/2159-8290.CD-13-0285 24436049 PMC4293454

[B21] Dick A. E. Gerlich D. W. (2013). Kinetic framework of spindle assembly checkpoint signalling. Nat. Cell Biol. 15, 1370–1377. 10.1038/ncb2842 24096243 PMC4067996

[B22] Dinarina A. Pugieux C. Corral M. M. Loose M. Spatz J. Karsenti E.. (2009). Chromatin shapes the mitotic spindle. Cell 138, 502–513. 10.1016/j.cell.2009.05.027 19665972

[B23] Ditchfield C. Johnson V. L. Tighe A. Ellston R. Haworth C. Johnson T.. (2003). Aurora B couples chromosome alignment with anaphase by targeting BubR1, Mad2, and Cenp-E to kinetochores. J. Cell Biol. 161, 267–280. 10.1083/jcb.200208091 12719470 PMC2172902

[B24] Dumont S. Mitchison T. J. (2009). Compression regulates mitotic spindle length by a mechanochemical switch at the poles. Curr. Biol. 19, 1086–1095. 10.1016/j.cub.2009.05.056 19540117 PMC2722833

[B25] Edgar B. A. Orr-Weaver T. L. (2001). Endoreplication cell cycles: more for less. Cell 105, 297–306. 10.1016/S0092-8674(01)00334-8 11348589

[B26] Etemad B. Kops G. J. (2016). Attachment issues: kinetochore transformations and spindle checkpoint silencing. Curr. Opin. Cell Biol. 39, 101–108. 10.1016/j.ceb.2016.02.016 26947988

[B27] Famulski J. K. Vos L. J. Rattner J. B. Chan G. K. (2011). Dynein/Dynactin-mediated transport of kinetochore components off kinetochores and onto spindle poles induced by nordihydroguaiaretic acid. PLoS One 6:e16494. 10.1371/journal.pone.0016494 21305043 PMC3030593

[B28] Foley E. A. Kapoor T. M. (2013). Microtubule attachment and spindle assembly checkpoint signalling at the kinetochore. Nat. Rev. Mol. Cell Biol. 14, 25–37. 10.1038/nrm3494 23258294 PMC3762224

[B29] Fujiwara T. Bandi M. Nitta M. Ivanova E. V. Bronson R. T. Pellman D. (2005). Cytokinesis failure generating tetraploids promotes tumorigenesis in p53-null cells. Nature 437:1043. 10.1038/nature04217 16222300

[B30] Galli M. Morgan D. O. (2016). Cell size determines the strength of the spindle assembly checkpoint during embryonic development. Dev. Cell 36, 344–352. 10.1016/j.devcel.2016.01.003 26859356 PMC4748171

[B31] Ganem N. J. Godinho S. A. Pellman D. (2009). A mechanism linking extra centrosomes to chromosomal instability. Nature 460:278. 10.1038/nature08136 19506557 PMC2743290

[B32] Ganem N. J. Storchova Z. Pellman D. (2007). Tetraploidy, aneuploidy and cancer. Curr. Opin. Genet. Dev. 17, 157–162. 10.1016/j.gde.2007.02.011 17324569

[B33] Gassmann R. Holland A. J. Varma D. Wan X. Civril F. Cleveland D. W.. (2010). Removal of Spindly from microtubule-attached kinetochores controls spindle checkpoint silencing in human cells. Genes Dev. 24, 957–971. 10.1101/gad.1886810 20439434 PMC2861194

[B34] Gelens L. Qian J. Bollen M. Saurin A. T. (2018). The importance of kinase-phosphatase integration: lessons from mitosis. Trends cell Biol. 28, 6–21. 10.1016/j.tcb.2017.09.005 29089159

[B35] Gerhold A. R. Poupart V. Labbé J.-C. Maddox P. S. (2018). Spindle assembly checkpoint strength is linked to cell fate in the *Caenorhabditis elegans* embryo. Mol. Biol. Cell 29, 1435–1448. 10.1091/mbc.E18-04-0215 29688794 PMC6014101

[B36] Gerhold A. R. Ryan J. Vallée-Trudeau J.-N. Dorn J. F. Labbé J.-C. Maddox P. S. (2015). Investigating the regulation of stem and progenitor cell mitotic progression by *in situ* imaging. Curr. Biol. 25, 1123–1134. 10.1016/j.cub.2015.02.054 25819563

[B37] Gillooly J. F. Hein A. Damiani R. (2015). Nuclear DNA content varies with cell size across human cell types. Cold Spring Harbor Perspect. Biol. 7:a019091. 10.1101/cshperspect.a019091 26134319 PMC4484964

[B38] Godinho S. A. Picone R. Burute M. Dagher R. Su Y. Leung C. T.. (2014). Oncogene-like induction of cellular invasion from centrosome amplification. Nature 510, 167–171. 10.1038/nature13277 24739973 PMC4061398

[B39] Good M. C. Vahey M. D. Skandarajah A. Fletcher D. A. Heald R. (2013). Cytoplasmic volume modulates spindle size during embryogenesis. Science 342, 856–860. 10.1126/science.1243147 24233724 PMC4094345

[B40] Gorbsky G. J. Chen R.-H. Murray A. W. (1998). Microinjection of antibody to Mad2 protein into mammalian cells in mitosis induces premature anaphase. J. Cell Biol. 141, 1193–1205. 10.1083/jcb.141.5.1193 9606211 PMC2137176

[B41] Gregory T. R. (2001). Coincidence, coevolution, or causation? DNA content, cell size, and the C-value enigma. Biol. Rev. 76, 65–101. 10.1017/S1464793100005595 11325054

[B42] Griffis E. R. Stuurman N. Vale R. D. (2007). Spindly, a novel protein essential for silencing the spindle assembly checkpoint, recruits dynein to the kinetochore. J. Cell Biol. 177, 1005–1015. 10.1083/jcb.200702062 17576797 PMC2064361

[B43] Hagting A. den Elzen N. Vodermaier H. C. Waizenegger I. C. Peters J.-M. Pines J. (2002). Human securin proteolysis is controlled by the spindle checkpoint and reveals when the APC/C switches from activation by Cdc20 to Cdh1. J. Cell Biol. 157, 1125–1137. 10.1083/jcb.200111001 12070128 PMC2173548

[B44] Hara Y. Kimura A. (2009). Cell-size-dependent spindle elongation in the *Caenorhabditis elegans* early embryo. Curr. Biol. 19, 1549–1554. 10.1016/j.cub.2009.07.050 19682904

[B45] Hauf S. Cole R. W. LaTerra S. Zimmer C. Schnapp G. Walter R.. (2003). The small molecule Hesperadin reveals a role for Aurora B in correcting kinetochore-microtubule attachment and in maintaining the spindle assembly checkpoint. J. Cell Biol. 161, 281–294. 10.1083/jcb.200208092 12707311 PMC2172906

[B46] Hazel J. Krutkramelis K. Mooney P. Tomschik M. Gerow K. Oakey J.. (2013). Changes in cytoplasmic volume are sufficient to drive spindle scaling. Science 342, 853–856. 10.1126/science.1243110 24233723 PMC4004590

[B47] Heald R. Gibeaux R. (2018). Subcellular scaling: does size matter for cell division? Curr. Opin. Cell Biol. 52, 88–95. 10.1016/j.ceb.2018.02.009 29501026 PMC5988940

[B48] Heasley L. R. Markus S. M. DeLuca J. G. (2017). “Wait anaphase” signals are not confined to the mitotic spindle. Mol. Biol. Cell 28, 1186–1194. 10.1091/mbc.e17-01-0036 28298492 PMC5415015

[B49] Howell B. McEwen B. Canman J. Hoffman D. Farrar E. Rieder C.. (2001). Cytoplasmic dynein/dynactin drives kinetochore protein transport to the spindle poles and has a role in mitotic spindle checkpoint inactivation. J. Cell Biol. 155, 1159–1172. 10.1083/jcb.200105093 11756470 PMC2199338

[B50] Howell B. J. Hoffman D. Fang G. Murray A. Salmon E. (2000). Visualization of Mad2 dynamics at kinetochores, along spindle fibers, and at spindle poles in living cells. J. Cell Biol. 150, 1233–1250. 10.1083/jcb.150.6.1233 10995431 PMC2150717

[B51] Jorgensen P. Edgington N. P. Schneider B. L. Rupeš I. Tyers M. Futcher B. (2007). The size of the nucleus increases as yeast cells grow. Mol. Biol. Cell 18, 3523–3532. 10.1091/mbc.e06-10-0973 17596521 PMC1951755

[B52] Khodjakov A. Rieder C. L. (2009). The nature of cell-cycle checkpoints: facts and fallacies. J. Biol. 8:88. 10.1186/jbiol195 19930621 PMC2790835

[B53] King S. J. Schroer T. A. (2000). Dynactin increases the processivity of the cytoplasmic dynein motor. Nat. Cell Biol. 2, 20–24. 10.1038/71338 10620802

[B54] Krüger L. K. Sanchez J.-L. Paoletti A. Tran P. T. (2019). Kinesin-6 regulates cell-size-dependent spindle elongation velocity to keep mitosis duration constant in fission yeast. Elife 8:e42182. 10.7554/eLife.42182.037 30806623 PMC6391065

[B55] Kunda P. Baum B. (2009). The actin cytoskeleton in spindle assembly and positioning. Trends Cell Biol. 19, 174–179. 10.1016/j.tcb.2009.01.006 19285869

[B56] Kunda P. Pelling A. E. Liu T. Baum B. (2008). Moesin controls cortical rigidity, cell rounding, and spindle morphogenesis during mitosis. Curr. Biol. 18, 91–101. 10.1016/j.cub.2007.12.051 18207738

[B57] Kuznetsova A. Y. Seget K. Moeller G. K. de Pagter M. S. de Roos J. A. Dürrbaum M.. (2015). Chromosomal instability, tolerance of mitotic errors and multidrug resistance are promoted by tetraploidization in human cells. Cell Cycle 14, 2810–2820. 10.1080/15384101.2015.1068482 26151317 PMC4614355

[B58] Kyogoku H. Kitajima T. S. (2017). Large cytoplasm is linked to the error-prone nature of oocytes. Dev. Cell 41, 287–298.e284. 10.1016/j.devcel.2017.04.009 28486131

[B59] Lacroix B. Letort G. Pitayu L. Sallé J. Stefanutti M. Maton G.. (2018). Microtubule dynamics scale with cell size to set spindle length and assembly timing. Dev. Cell 45, 496–511.e496. 10.1016/j.devcel.2018.04.022 29787710 PMC6360954

[B60] Lancaster O. M. Le Berre M. Dimitracopoulos A. Bonazzi D. Zlotek-Zlotkiewicz E. Picone R.. (2013). Mitotic rounding alters cell geometry to ensure efficient bipolar spindle formation. Dev. Cell 25, 270–283. 10.1016/j.devcel.2013.03.014 23623611

[B61] Lang F. (2007). Mechanisms and significance of cell volume regulation. J. Am. Coll. Nutr. 26, 613S−623S. 10.1080/07315724.2007.10719667 17921474

[B62] Leitao R. M. Kellogg D. R. (2017). The duration of mitosis and daughter cell size are modulated by nutrients in budding yeast. J. Cell Biol. 216, 3463–3470. 10.1083/jcb.201609114 28939614 PMC5674877

[B63] Lénárt P. Bacher C. P. Daigle N. Hand A. R. Eils R. Terasaki M.. (2005). A contractile nuclear actin network drives chromosome congression in oocytes. Nature 436, 812–818. 10.1038/nature03810 16015286

[B64] Liu D. Vader G. Vromans M. J. Lampson M. A. Lens S. M. (2009). Sensing chromosome bi-orientation by spatial separation of aurora B kinase from kinetochore substrates. Science 323, 1350–1353. 10.1126/science.1167000 19150808 PMC2713345

[B65] Loughlin R. Heald R. Nédélec F. (2010). A computational model predicts Xenopus meiotic spindle organization. J. Cell Biol. 191, 1239–1249. 10.1083/jcb.201006076 21173114 PMC3010074

[B66] Loughlin R. Wilbur J. D. McNally F. J. Nédélec F. J. Heald R. (2011). Katanin contributes to interspecies spindle length scaling in Xenopus. Cell 147, 1397–1407. 10.1016/j.cell.2011.11.014 22153081 PMC3240848

[B67] Maresca T. J. Salmon E. D. (2009). Intrakinetochore stretch is associated with changes in kinetochore phosphorylation and spindle assembly checkpoint activity. J. Cell Biol. 184, 373–381. 10.1083/jcb.200808130 19193623 PMC2646557

[B68] Matson D. R. Stukenberg P. T. (2014). CENP-I and Aurora B act as a molecular switch that ties RZZ/Mad1 recruitment to kinetochore attachment status. J. Cell Biol. 205, 541–554. 10.1083/jcb.201307137 24862574 PMC4033774

[B69] Mayer V. W. Goin C. J. Arras C. A. Taylor-Mayer R. E. (1992). Comparison of chemically induced chromosome loss in a diploid, triploid, and tetraploid strain of *Saccharomyces cerevisiae*. Mutat. Res. Genet. Toxicol. 279, 41–48. 10.1016/0165-1218(92)90264-Z 1374531

[B70] McCleland M. L. Farrell J. A. O'Farrell P. H. (2009). Influence of cyclin type and dose on mitotic entry and progression in the early Drosophila embryo. J. Cell Biol. 184, 639–646. 10.1083/jcb.200810012 19273612 PMC2686416

[B71] Meraldi P. Draviam V. M. Sorger P. K. (2004). Timing and checkpoints in the regulation of mitotic progression. Dev. Cell 7, 45–60. 10.1016/j.devcel.2004.06.006 15239953

[B72] Mogilner A. Craig E. (2010). Towards a quantitative understanding of mitotic spindle assembly and mechanics. J. Cell Sci. 123, 3435–3445. 10.1242/jcs.062208 20930139 PMC2951465

[B73] Mortimer R. K. (1958). Radiobiological and genetic studies on a polyploid series (haploid to hexaploid) of *Saccharomyces cerevisiae*. Radiat. Res. 9, 312–326. 10.2307/3570795 13579200

[B74] Moura M. Osswald M. Leça N. Barbosa J. Pereira A. J. Maiato H.. (2017). Protein phosphatase 1 inactivates Mps1 to ensure efficient spindle assembly checkpoint silencing. Elife 6:e25366. 10.7554/eLife.25366.040 28463114 PMC5433843

[B75] Musacchio A. (2011). Spindle assembly checkpoint: the third decade. Philos. Trans. R. Soc. B Biol. Sci. 366, 3595–3604. 10.1098/rstb.2011.0072 22084386 PMC3203455

[B76] Musacchio A. (2015). The molecular biology of spindle assembly checkpoint signaling dynamics. Curr. Biol. 25, R1002–R1018. 10.1016/j.cub.2015.08.051 26485365

[B77] Neumann F. R. Nurse P. (2007). Nuclear size control in fission yeast. J. Cell Biol. 179, 593–600. 10.1083/jcb.200708054 17998401 PMC2080919

[B78] Nijenhuis W. Vallardi G. Teixeira A. Kops G. J. Saurin A. T. (2014). Negative feedback at kinetochores underlies a responsive spindle checkpoint signal. Nat. Cell Biol. 16, 1257–1264. 10.1038/ncb3065 25402682 PMC6485516

[B79] Paim L. M. G. FitzHarris G. (2019). Tetraploidy causes chromosomal instability in acentriolar mouse embryos. Nat. Commun. 10, 1–12. 10.1038/s41467-019-12772-8 31645568 PMC6811537

[B80] Pereira A. J. Maiato H. (2012). Maturation of the kinetochore-microtubule interface and the meaning of metaphase. Chromosome Res. 20, 563–577. 10.1007/s10577-012-9298-8 22801775

[B81] Potapova T. Gorbsky G. J. (2017). The consequences of chromosome segregation errors in mitosis and meiosis. Biology 6:12. 10.3390/biology6010012 28208750 PMC5372005

[B82] Potapova T. A. Seidel C. W. Box A. C. Rancati G. Li R. (2016). Transcriptome analysis of tetraploid cells identifies cyclin D2 as a facilitator of adaptation to genome doubling in the presence of p53. Mol. Biol. Cell 27, 3065–3084. 10.1091/mbc.e16-05-0268 27559130 PMC5063615

[B83] Quinton R. J. DiDomizio A. Vittoria M. A. Ticas C. J. Patel S. Koga Y. (2020). Whole genome doubling confers unique genetic vulnerabilities on tumor cells. bioRxiv [Preprint]. 10.1101/2020.06.18.159095 PMC7889737 33505027

[B84] Rasband W. S. (2011). National Institutes of Health. Bethesda, MD. Available online at: http://imagej.nih.gov/ij/.

[B85] Reber S. B. Baumgart J. Widlund P. O. Pozniakovsky A. Howard J. Hyman A. A.. (2013). XMAP215 activity sets spindle length by controlling the total mass of spindle microtubules. Nat. Cell Biol. 15, 1116–1122. 10.1038/ncb2834 23974040

[B86] Reck-Peterson S. L. Yildiz A. Carter A. P. Gennerich A. Zhang N. Vale R. D. (2006). Single-molecule analysis of dynein processivity and stepping behavior. Cell 126, 335–348. 10.1016/j.cell.2006.05.046 16873064 PMC2851639

[B87] Rieder C. L. Cole R. W. Khodjakov A. Sluder G. (1995). The checkpoint delaying anaphase in response to chromosome monoorientation is mediated by an inhibitory signal produced by unattached kinetochores. J. Cell Biol. 130, 941–948. 10.1083/jcb.130.4.941 7642709 PMC2199954

[B88] Rieder C. L. Maiato H. (2004). Stuck in division or passing through: what happens when cells cannot satisfy the spindle assembly checkpoint. Dev. Cell 7, 637–651. 10.1016/j.devcel.2004.09.002 15525526

[B89] Rieder C. L. Schultz A. Cole R. Sluder G. (1994). Anaphase onset in vertebrate somatic cells is controlled by a checkpoint that monitors sister kinetochore attachment to the spindle. J. Cell Biol. 127, 1301–1310. 10.1083/jcb.127.5.1301 7962091 PMC2120267

[B90] Sacristan C. Kops G. J. (2015). Joined at the hip: kinetochores, microtubules, and spindle assembly checkpoint signaling. Trends Cell Biol. 25, 21–28. 10.1016/j.tcb.2014.08.006 25220181

[B91] Saurin A. T. (2018). Kinase and phosphatase cross-talk at the kinetochore. Front. Cell Dev. Biol. 6:62. 10.3389/fcell.2018.00062 29971233 PMC6018199

[B92] Shah J. V. Botvinick E. Bonday Z. Furnari F. Berns M. Cleveland D. W. (2004). Dynamics of centromere and kinetochore proteins: implications for checkpoint signaling and silencing. Curr. Biol. 14, 942–952. 10.1016/S0960-9822(04)00381-1 15182667

[B93] Sikora-Polaczek M. Hupalowska A. Polanski Z. Kubiak J. Z. Ciemerych M. A. (2006). The first mitosis of the mouse embryo is prolonged by transitional metaphase arrest. Biol. Reprod. 74, 734–743. 10.1095/biolreprod.105.047092 16382027

[B94] Silkworth W. T. Nardi I. K. Scholl L. M. Cimini D. (2009). Multipolar spindle pole coalescence is a major source of kinetochore mis-attachment and chromosome mis-segregation in cancer cells. PLoS One 4:e6564. 10.1371/journal.pone.0006564 19668340 PMC2719800

[B95] Silva P. M. Reis R. M. Bolanos-Garcia V. M. Florindo C. Tavares A. A. Bousbaa H. (2014). Dynein-dependent transport of spindle assembly checkpoint proteins off kinetochores toward spindle poles. FEBS Lett. 588, 3265–3273. 10.1016/j.febslet.2014.07.011 25064841

[B96] Sivakumar S. Daum J. R. Tipton A. R. Rankin S. Gorbsky G. J. (2014). The spindle and kinetochore-associated (Ska) complex enhances binding of the anaphase-promoting complex/cyclosome (APC/C) to chromosomes and promotes mitotic exit. Mol. Biol. Cell 25, 594–605. 10.1091/mbc.e13-07-0421 24403607 PMC3937086

[B97] Smith R. J. Cordeiro M. H. Davey N. E. Vallardi G. Ciliberto A. Gross F.. (2019). PP1 and PP2A use opposite phospho-dependencies to control distinct processes at the kinetochore. Cell Rep. 28, 2206–2219.e2208. 10.1016/j.celrep.2019.07.067 31433993 PMC6715587

[B98] Stewart M. P. Helenius J. Toyoda Y. Ramanathan S. P. Muller D. J. Hyman A. A. (2011). Hydrostatic pressure and the actomyosin cortex drive mitotic cell rounding. Nature 469, 226–230. 10.1038/nature09642 21196934

[B99] Storchová Z. Breneman A. Cande J. Dunn J. Burbank K. O'Toole E.. (2006). Genome-wide genetic analysis of polyploidy in yeast. Nature 443:541. 10.1038/nature05178 17024086

[B100] Storchova Z. Kuffer C. (2008). The consequences of tetraploidy and aneuploidy. J. Cell Sci. 121, 3859–3866. 10.1242/jcs.039537 19020304

[B101] Storchova Z. Pellman D. (2004). From polyploidy to aneuploidy, genome instability and cancer. Nat. Rev. Mol. Cell Biol. 5, 45–54. 10.1038/nrm1276 14708009

[B102] Tauchman E. C. Boehm F. J. DeLuca J. G. (2015). Stable kinetochore-microtubule attachment is sufficient to silence the spindle assembly checkpoint in human cells. Nat. Commun. 6, 1–9. 10.1038/ncomms10036 26620470 PMC4686653

[B103] Taylor S. S. Scott M. I. Holland A. J. (2004). The spindle checkpoint: a quality control mechanism which ensures accurate chromosome segregation. Chromosome Res. 12, 599–616. 10.1023/B:CHRO.0000036610.78380.51 15289666

[B104] Torres J. Z. Ban K. H. Jackson P. K. (2010). A specific form of phospho protein phosphatase 2 regulates anaphase-promoting complex/cyclosome association with spindle poles. Mol. Biol. Cell 21, 897–904. 10.1091/mbc.e09-07-0598 20089842 PMC2836970

[B105] Tyson J. J. Chen K. C. Novak B. (2003). Sniffers, buzzers, toggles and blinkers: dynamics of regulatory and signaling pathways in the cell. Curr. Opin. Cell Biol. 15, 221–231. 10.1016/S0955-0674(03)00017-6 12648679

[B106] Vassilev L. T. (2006). Cell cycle synchronization at the G2/M phase border by reversible inhibition of CDK1. Cell Cycle 5, 2555–2556. 10.4161/cc.5.22.3463 17172841

[B107] Vázquez-Diez C. Paim L. M. G. FitzHarris G. (2019). Cell-size-independent spindle checkpoint failure underlies chromosome segregation error in mouse embryos. Curr. Biol. 29, 865–873.e863. 10.1016/j.cub.2018.12.042 30773364

[B108] Viganó C. von Schubert C. Ahrné E. Schmidt A. Lorber T. Bubendorf L.. (2018). Quantitative proteomic and phosphoproteomic comparison of human colon cancer DLD-1 cells differing in ploidy and chromosome stability. Mol. Biol. Cell 29, 1031–1047. 10.1091/mbc.E17-10-0577 29496963 PMC5921571

[B109] Wang N. Stenkvist B. Tribukait B. (1992). Morphometry of nuclei of the normal and malignant prostate in relation to DNA ploidy. Anal. Quant. Cytol. Histol. 14, 210–216. 1418270

[B110] Welburn J. P. Vleugel M. Liu D. Yates J. R. 3rd Lampson M. A. Fukagawa T.. (2010). Aurora B phosphorylates spatially distinct targets to differentially regulate the kinetochore-microtubule interface. Mol. Cell 38, 383–392. 10.1016/j.molcel.2010.02.034 20471944 PMC2873218

[B111] Whyte J. Bader J. R. Tauhata S. B. Raycroft M. Hornick J. Pfister K. K.. (2008). Phosphorylation regulates targeting of cytoplasmic dynein to kinetochores during mitosis. J. Cell Biol. 183, 819–834. 10.1083/jcb.200804114 19029334 PMC2592828

[B112] Wilbur J. D. Heald R. (2013). Mitotic spindle scaling during Xenopus development by kif2a and importin α. Elife 2:e00290. 10.7554/eLife.00290 23425906 PMC3576809

[B113] Wojcik E. Basto R. Serr M. Scaerou F. Karess R. Hays T. (2001). Kinetochore dynein: its dynamics and role in the transport of the Rough deal checkpoint protein. Nat. Cell Biol. 3, 1001–1007. 10.1038/ncb1101-1001 11715021

[B114] Zack T. I. Schumacher S. E. Carter S. L. Cherniack A. D. Saksena G. Tabak B.. (2013). Pan-cancer patterns of somatic copy number alteration. Nat. Genet. 45, 1134–1140. 10.1038/ng.2760 24071852 PMC3966983

