# Supplementary material for: Spindle Architectural Features Must Be Considered Along With Cell Size to Explain the Timing of Mitotic Checkpoint Silencing
Source: Front Physiol. 2021 Jan 28;11:596263. doi: 10.3389/fphys.2020.596263 (PMC7877541; doi:10.3389/fphys.2020.596263)
Supplement: Supplementary file 16 [file Data_Sheet_1.pdf]

Supplementary Materials

Estimating spindle pole size from γ-tubulin fluorescence intensity

The measured γ-tubulin intensity at the spindle pole reflects the abundance of γ-tubulin ring complexes (γ-TuRC). We assumed the abundance of γ-TuRCs to be proportional to the surface area of the spindle pole, i.e., γ-tubulin intensity $\sim d^2$, where d is the diameter of the spindle pole. Furthermore, smaller spindle pole size gives rise to higher spindle pole signal in the model (**Supplementary Figure 6D**), and hence SAC silencing is expected to be first initiated from the smaller spindle pole out of the two. In this light, we took the lower γ-tubulin intensity out of the two poles to derive the spindle pole size. Taken together, the diameter of the spindle pole was estimated by

$$d = C\sqrt{\frac{\min\left(\gamma\text{-Tub intensity at Pole 1, }\gamma\text{-Tub intensity at Pole 2}\right)}{\pi}} \quad \text{(S1)}$$

We chose $C = 1/11$ for 2N and 4N RPE-1 and $C = 1/15$ for 2N and 4N DLD-1 to make the predicted metaphase durations fall in the range of the observed data. Since the same value is used for all the clones deriving from the same ancestor, the predicted metaphase duration can be compared among these clones. The difference of C value between RPE-1 and DLD-1 cell lines could reflect their difference in gamma-tubulin density at the spindle pole (which results in different factors of conversion from fluorescence intensity to size of spindle pole).

Estimating microtubule numbers from α-tubulin fluorescence intensity

The measured total intensity of α-tubulin in the spindle reflects the total length of microtubules in the spindle. This total length depends on both spindle dimensions and spindle microtubule density, and the relationship is modeled as described below. To mathematically characterize the decrease of microtubule density with the increase of distance from the spindle pole, the model assumes two subpopulations of spindle microtubules (**Supplementary Figures 8A,B**): a subpopulation MT1 (**Supplementary Figures 8A,B, red**), with a density that scales with inverse square of the distance from the spindle pole, and a subpopulation MT2 (**Supplementary Figures 8A,B, blue**), with a constant density throughout the spindle. MT1 represents the microtubules emanating from the spindle poles, and microtubules that may be seeded by these pole-originated microtubules and follow similar inverse square scaling of density. MT2 represents the remaining microtubules that are dispersed in the spindle and presumably promoted

by other spindle-associated factors such as Ran GTP; the spatial distribution of MT2 is not known and assumed to be homogenous in the spindle for simplicity.

The abundance of MT1 is characterized by its number, N_{MT1}, and that of MT2 by its constant density, ρ_{MT2}. Let L, W and H be the length, width and height of the spindle, respectively. For the sake of computational efficiency (see "Method for stochastic simulation"), in the model we assume a simplified spindle geometry with equal width and height, but the same area of the spindle equator as measured, i.e., $H = W = \sqrt{\text{measured spindle width } \times \text{measured spindle height}}$ (**Table 1**; this geometric adjustment has negligible impact on the model results). The total length of microtubules in a spindle is then given by Eq. (S2) (see derivation in the next section, "Deriving total spindle microtubule length").

$$L_{\mathrm{MT}} = N_{\mathrm{MT1}}\left(\frac{L\ln\sqrt{\frac{L^2+W^2}{L^2}}}{2\left(1-\sqrt{\frac{L^2}{L^2+W^2}}\right)}-\frac{d}{2}\right)+\rho_{\mathrm{MT2}}\left(\frac{\pi}{12}LW^2-\frac{\pi d^3}{6}\left(1-\sqrt{\frac{L^2}{L^2+W^2}}\right)\right) \qquad \text{(S2)}$$

To make the total length of microtubules in a modeled spindle proportional to the measured α-tubulin intensity, we took the following steps to derive the corresponding number of microtubules:

1. We assumed that in the parental DLD-1 cell, $N_{\mathrm{MT1}} = 1600$ (800 in each half spindle), $\rho_{\mathrm{MT2}} = 10\ \mu\mathrm{m}^{-2}$. Plugging these parameters and the measured spindle dimensions for the parental DLD-1 cells into Eq. (S2) yields the total microtubule length of ~6800 μm.
2. For all the 4N DLD-1 clones as well as the 2N and 4N RPE-1 cells, we estimate the total spindle microtubule length by $L_{\mathrm{MT}} = 6800\ \mu\mathrm{m} \div \alpha\text{Tub intensity in parental cell} \times \alpha\text{Tub intensity in target cell}$.
3. For clones with significantly higher microtubule density, e.g., S1 and L1, the α-tubulin intensity is heavily enhanced around the spindle pole (**Figure 2A**). This indicates that the difference in α-tubulin intensity is mainly attributed to the difference in MT1 abundance, rather than MT2 abundance. Hence, we assume $\rho_{\mathrm{MT2}} = 10\ \mu\mathrm{m}^{-2}$ in all cell clones. N_{MT1} of the clone of interest was then calculated from the value of L_{MT} estimated in Step 2, using Eq. (S2) and the median spindle dimensions measured for the clone.

Deriving total spindle microtubule length

The total spindle microtubule length is the sum of the lengths of microtubules in the two subpopulations assumed in the model (see previous section). That is,

$$L_{\mathrm{MT}} = L_{\mathrm{MT1}} + L_{\mathrm{MT2}} \tag{S3}$$

To calculate the total length of MT1, we first converted N_1 into a microtubule density to allow an integral expression of the total MT1 length. We set ρ_{MT01} to be the density of MT1 crossing the spherical surface 1 μm away from the center of the spindle pole (**Supplementary Figure 8C**), i.e.,

$$\rho_{\mathrm{MT10}} = \frac{N_1/2}{A_0} \tag{S4}$$

The area of the spherical surface encompassed within the spindle, A_0, can be calculated by

$$A_0 = \int_0^{2\pi} d\theta \int_0^{\varphi_{\max}(\theta)} 1\,\mu\mathrm{m}^2 \sin\varphi d\varphi \tag{S5}$$

where φ and θ are the polar and azimuthal angles, respectively, of the spherical coordinate system. $\varphi_{\max}(\theta)$ is the maximum polar angle in the spindle for a given azimuthal angle, θ. $\varphi_{\max}(\theta)$ corresponds to the polar angle of the point on the periphery of the spindle equator with azimuthal angle, θ (**Supplementary Figure 8D**, Point A). The function, $\varphi_{\max}(\theta)$, is derived below.

For a spindle with length, L, width, W, and height, H, the peripheral of the spindle equator satisfies

$$\frac{y^2}{(W/2)^2} + \frac{z^2}{(H/2)^2} = 1 \tag{S6}$$

Point A also satisfies

$$z_A = y_A \tan\theta \tag{S7}$$

Combining Eqs. (S6) and (S7) yields

$$y_A = \frac{WH}{2\sqrt{H^2 + W^2 \tan^2\theta}} \tag{S8}$$

The distance between Point A and the center of the spindle equator (Point O) is $y_A \sec\theta$. The distance between Point O and the spindle pole is $L/2$. Therefore, the polar angle of Point A is

$$\varphi_{\max}(\theta) = \arctan\frac{y_A \sec\theta}{L/2} = \arctan\frac{1}{\sqrt{\left(\frac{L}{W}\cos\theta\right)^2 + \left(\frac{L}{H}\sin\theta\right)^2}} \tag{S9}$$

Hence,

$$\cos\varphi_{\max}(\theta) = \sqrt{\frac{Q(\theta)}{1+Q(\theta)}} \tag{S10}$$

where $$Q(\theta) = \left(\frac{L}{W}\cos\theta\right)^2 + \left(\frac{L}{H}\sin\theta\right)^2$$

Plugging Eq. (S10) into Eq. (S5) yields

$$A_0 = \int_0^{2\pi} 1 - \sqrt{\frac{Q(\theta)}{1+Q(\theta)}} d\theta \left[\mu\text{m}^2\right] \tag{S11}$$

Note that the number of MT1 passing through the infinitesimal surface element $d\varphi d\theta$ on a spherical surface is $\rho_{\text{MT10}}\left(1\ \mu\text{m}^2\right)\sin\varphi d\varphi d\theta$. Hence, the total MT1 length is

$$\begin{aligned} L_{\text{MT1}} &= 2\rho_{\text{MT10}}\left(1\ \mu\text{m}^2\right)\int_0^{2\pi} d\theta \int_0^{\varphi_{\max}(\theta)} \left(\frac{L}{2\cos\varphi} - \frac{d}{2}\right)\sin\varphi d\varphi \\ &= \rho_{\text{MT10}}\left(1\ \mu\text{m}^2\right)\int_0^{2\pi} -L\ln\cos\varphi_{\max}(\theta) - d\left(1-\cos\varphi_{\max}(\theta)\right)d\theta \\ &= \rho_{\text{MT10}}\left(1\ \mu\text{m}^2\right)\int_0^{2\pi} L\ln\sqrt{\frac{1+Q(\theta)}{Q(\theta)}} - d\left(1-\sqrt{\frac{Q(\theta)}{1+Q(\theta)}}\right)d\theta \end{aligned} \tag{S12}$$

Combining Eqs. (S4)-(S12) gives

$$L_{\text{MT1}} = N_{\text{MT1}} \frac{\int_0^{2\pi} L\ln\sqrt{\frac{1+Q(\theta)}{Q(\theta)}} - d\left(1-\sqrt{\frac{Q(\theta)}{1+Q(\theta)}}\right)d\theta}{2\int_0^{2\pi} 1-\sqrt{\frac{Q(\theta)}{1+Q(\theta)}}d\theta} \tag{S13}$$

The total length of MT2 is derived as:

$$L_{\mathrm{MT2}} = \rho_{\mathrm{MT2}} V_{\mathrm{S}} \tag{S14}$$

where V_{S} is the volume of the spindle,

$$V_{\mathrm{S}} = \frac{\pi}{12} LWH - V_{\mathrm{SP/S}} \tag{S15}$$

and $V_{\mathrm{SP/S}}$ is the volume of the two small, cone-shaped intersections between the spindle and two spindle poles.

Each of the SP/S intersection assumes the shape of an elliptical cone capped by a spherical bottom. Hence,

$$\begin{aligned} V_{\mathrm{SP/S}} &= 2\int_0^{2\pi} d\theta \int_0^{\varphi_{\max}(\theta)} \sin\varphi d\varphi \int_0^{d/2} r^2 dr \\ &= \frac{d^3}{12}\int_0^{2\pi} 1 - \cos\varphi_{\max}(\theta) d\theta \\ &= \frac{d^3}{12}\int_0^{2\pi} 1 - \sqrt{\frac{Q(\theta)}{1+Q(\theta)}} d\theta \end{aligned} \tag{S16}$$

where r is the distance to the spindle pole, and $\varphi_{\max}(\theta)$ is the same maximum polar angle for a given azimuthal angle, θ, as defined above (**Supplementary Figure 8D**).

Combining Eqs. (S14)-(S16),

$$L_{\mathrm{MT2}} = \rho_{\mathrm{MT2}} \left(\frac{\pi}{12} LWH - \frac{d^3}{12}\int_0^{2\pi} 1 - \sqrt{\frac{Q(\theta)}{1+Q(\theta)}} d\theta \right) \tag{S17}$$

In the end, the total length of spindle microtubules is

$$\begin{aligned} L_{\mathrm{MT}} &= L_{\mathrm{MT1}} + L_{\mathrm{MT2}} \\ &= N_{\mathrm{MT1}} \frac{\int_0^{2\pi} L \ln\sqrt{\frac{1+Q(\theta)}{Q(\theta)}} - d\left(1 - \sqrt{\frac{Q(\theta)}{1+Q(\theta)}}\right) d\theta}{2\int_0^{2\pi} 1 - \sqrt{\frac{Q(\theta)}{1+Q(\theta)}} d\theta} \\ &\quad + \rho_{\mathrm{MT2}} \left(\frac{\pi}{12} LWH - \frac{d^3}{12}\int_0^{2\pi} 1 - \sqrt{\frac{Q(\theta)}{1+Q(\theta)}} d\theta \right) \end{aligned} \tag{S18}$$

Note that in the SAC silencing model, we assumed a simplified geometry with equal spindle width and height, but the same area of the spindle equator as measured, i.e., $H = W = \sqrt{\text{measured spindle width} \times \text{measured spindle height}}$, which allows reduction of the geometry to a 2D axisymmetric setting in the computationally expensive stochastic simulation (see "Method for stochastic simulation" below). In this simplified geometry, Eq. (S18) can be further simplified as the following:

$$Q(\theta) = \frac{L^2}{W^2}, \text{ for any } \theta \tag{S19}$$

$$\cos\varphi_{\max}(\theta) = \sqrt{\frac{L^2}{L^2 + W^2}}, \text{ for any } \theta \tag{S20}$$

Plugging Eq. (S20) into Eq. (S18) yields Eq. (S2).

Core model for spindle-mediated spatial regulation of SAC proteins

The core model focuses on how dynein-mediated poleward flux along the spindle regulates concentration of the SAC proteins at the spindle pole as more kinetochores become attached. This core model is formulated as a system of compartmentalized diffusion-advection-reaction partial differential equations (PDE) [1-3]. The model cell consists of the following spatial compartments: a spherical cytoplasm, a bipolar spindle, 2 spherical spindle poles and N spherical kinetochores (**Figure 5B**). Let $Y_{00}, Y_0, Y_1, Y_\text{K}$, and Y_P stand for the SAC proteins that are transport inactive (00), transport active but unbound from the microtubule (0), transport active and traveling along the microtubule (1), sequestered at the kinetochore (K), and sequestered at the spindle pole (P), respectively. The PDEs describing the spatiotemporal dynamics of the SAC proteins as kinetochores get attached consecutively are given below.

Equations in the cytoplasm:

$$\frac{\partial Y_1}{\partial t} = \underbrace{D_\text{MT}\nabla^2 Y_1}_{\text{Diffusion}} \underbrace{- V\left(-\tilde{\mathbf{r}}^\text{SP} / \left|\tilde{\mathbf{r}}^\text{SP}\right|\right) \cdot \nabla Y_1}_{\text{Poleward streaming}} \underbrace{+ k_\text{onMT}\rho_\text{MT} Y_0 - k_\text{offMT} Y_1}_{\text{Binding/unbinding to microtubules}} \tag{S21}$$

$$\frac{\partial Y_0}{\partial t} = \underbrace{D_\text{Dyn}\nabla^2 Y_0}_{\text{Diffusion}} \underbrace{+ D_\text{Dyn}\frac{\nabla U_\text{ext}}{k_\text{B}T} \cdot \nabla Y_0}_{\text{Sequestration by spindle}} \underbrace{- k_\text{onMT}\rho_\text{MT} Y_0 + k_\text{offMT} Y_1}_{\text{Binding/unbinding to microtubules}} \tag{S22}$$

$$\frac{\partial Y_{00}}{\partial t} = \underbrace{D_Y \nabla^2 Y_{00}}_{\text{Diffusion}} \tag{S23}$$

Eqs. (S21)-(S23) all assume a reflective boundary condition at the cell boundary.

In Eq. (S21), $\left(-\tilde{\mathbf{r}}^{\text{SP}}/\left|\tilde{\mathbf{r}}^{\text{SP}}\right|\right)$ is the unit vector pointing towards the nearest spindle pole, which defines the direction of local velocity of dynein-transported SAC proteins. The binding rate of transport-active proteins to the microtubules is proportional to the local microtubule density, ρ_{MT}, which is the sum of MT1 and MT2 densities inside the spindle, and astral microtubule density outside the spindle (Eq. (S24) below). In Eq. (S22), a potential, U_{ext}, phenomenologically captures an enrichment of SAC proteins inside the spindle. Factors that retain SAC proteins within the spindle apparatus can be rather diverse, such as association with spindle matrix [4-6] or peri-spindle membranous networks [6, 7]. The model assumes a weak potential of $2k_BT$ (unit of thermal energy) across a 1-μm wide sheath surrounding the spindle boundary on the transport-active proteins (because dynein is shown to enrich in the spindle [8]).

$$\rho_{\text{MT}}(\mathbf{r}) = \begin{cases} \dfrac{N_{\text{MT1}}}{2\pi\left|\tilde{\mathbf{r}}^{\text{SP}}\right|^2(1-\cos\alpha)} + \rho_{\text{MT2}}, & \text{inside spindle (MT1 + MT2)} \\ \dfrac{N_{\text{aMT}}}{2\pi\left|\tilde{\mathbf{r}}^{\text{SP}}\right|^2(1+\cos\alpha)}, & \text{outside spindle (astral MT)} \end{cases} \tag{S24}$$

where $\left|\tilde{\mathbf{r}}^{\text{SP}}\right|$ is the distance towards the nearest spindle pole. α is half the vertex angle of the half spindle, i.e., $\alpha = \arctan(W/L)$. For simplicity, the number of astral microtubules is assumed to be the same as the number of MT1, i.e., $N_{\text{aMT}} = N_{\text{MT1}}$. Eq. (S24) represents the spatial distribution of the mean microtubule density, without resolving individual microtubules.

Equations at the spindle pole:

$$\frac{\partial Y_1}{\partial t} = D_{\text{MT}}\nabla^2 Y_1 - V\left(-\tilde{\mathbf{r}}^{\text{SP}}/\left|\tilde{\mathbf{r}}^{\text{SP}}\right|\right)\cdot\nabla Y_1 + k_{\text{onMT}}^{\text{SP}} Y_0 - k_{\text{offMT}}^{\text{SP}} Y_1 \underbrace{-k_{\text{onSP}} Y_1}_{\text{Sequestration by spindle pole}} \tag{S25}$$

$$\frac{\partial Y_0}{\partial t} = D_{\text{Dyn}}\nabla^2 Y_0 - k_{\text{onMT}}^{\text{SP}} Y_0 + k_{\text{offMT}}^{\text{SP}} Y_1 \underbrace{-k_{\text{onSP}} Y_0 + k_{\text{offSP}} Y_{\text{P}}}_{\text{Sequestration/release at spindle pole}} \tag{S26}$$

$$\frac{\partial Y_{00}}{\partial t} = D_{\text{Y}}\nabla^2 Y_{00} \tag{S27}$$

$$\frac{\partial Y_{\mathrm{P}}}{\partial t}=D_{\mathrm{P}}\nabla^{2}Y_{\mathrm{P}}\underbrace{+k_{\mathrm{onSP}}\left(Y_{0}+Y_{1}\right)}_{\text{Sequestration by spindle pole}}\underbrace{-k_{\mathrm{offSP}}Y_{\mathrm{P}}}_{\text{Release by spindle pole}} \tag{S28}$$

The spindle pole domain harbors an additional state for the spindle-pole bound proteins (Y_{P}). Y_{P} resides exclusively in the spindle pole domain, defined by a reflective boundary condition at the spindle pole boundary. Simple diffusion is assumed for Y_{P} to homogenize its concentration within the spindle pole, such that its spatial dynamics depend on the average concentration of SAC proteins at the spindle pole. Y_1, Y_0 and Y_{00} see the spindle pole as a virtual domain and assume continuity conditions at the spindle pole boundary.

Because microtubules cannot extend far into the spindle pole, upon entering the spindle pole the transport-active proteins immediately fall off the microtubules ($k_{\mathrm{offMT}}^{\mathrm{SP}}=40\ s^{-1}$, $k_{\mathrm{onMT}}^{\mathrm{SP}}=0$). Furthermore, they bind to the spindle pole with rate k_{onSP}, and dissociate from the spindle pole with rate k_{offSP}. Overall, Eqs. (S25)-(S28) characterize the fluxes of SAC proteins and their partial sequestration at the spindle pole.

Equation at the *n*-th kinetochore:

$$\frac{\partial Y_{\mathrm{K}n}}{\partial t}=D_{\mathrm{K}}\nabla^{2}Y_{\mathrm{K}n} \tag{S29}$$

Simple diffusion is assumed in Eq. (S29) to homogenize the concentration of kinetochore-bound proteins ($Y_{\mathrm{K}n}$) within the kinetochore, such that the boundary flux only depends on the average concentration of SAC proteins at the kinetochore. The binding/unbinding dynamics to the kinetochore is further characterized by the following flux boundary conditions at the kinetochore boundaries.

Boundary condition for unattached kinetochores:

$$-\mathbf{n}\cdot\Gamma_{\mathrm{YK}n}=\underbrace{k_{\mathrm{onKTu}}\left(1-Y_{\mathrm{K}n}/Y_{\mathrm{K}}^{\max}\right)\left(Y_{0}+Y_{00}\right)}_{\text{Recruitment onto unattached kinetochore}}\underbrace{-k_{\mathrm{offKT}}Y_{\mathrm{K}n}}_{\text{Turnover of diffusive SAC proteins into cytoplasm}} \tag{S30}$$

$$-\mathbf{n}\cdot\Gamma_{\mathrm{Y1}}=0 \tag{S31}$$

$$-\mathbf{n}\cdot\Gamma_{\mathrm{Y0}}=-k_{\mathrm{onKTu}}\left(1-Y_{\mathrm{K}n}/Y_{\mathrm{K}}^{\max}\right)Y_{0} \tag{S32}$$

$$-\mathbf{n}\cdot\Gamma_{\mathrm{Y00}}=-k_{\mathrm{onKTu}}\left(1-Y_{\mathrm{K}n}/Y_{\mathrm{K}}^{\max}\right)Y_{00}+k_{\mathrm{offKT}}Y_{\mathrm{K}n} \tag{S33}$$

Boundary condition for attached kinetochores:

$$-\mathbf{n}\cdot\Gamma_{\mathrm{YK}n} = \underbrace{k_{\mathrm{onKTt}}\left(1-Y_{\mathrm{K}n}/Y_{\mathrm{K}}^{\max}\right)\left(Y_0+Y_{00}\right)}_{\text{Recruitment onto attached kinetochore}} \underbrace{-\left(k_{\mathrm{offKT}}+k_{\mathrm{DoffKT}}\right)Y_{\mathrm{K}n}}_{\substack{\text{Turnover of diffusive SAC proteins}\\ \text{and release of streaming SAC proteins}}} \tag{S34}$$

$$-\mathbf{n}\cdot\Gamma_{\mathrm{Y1}} = k_{\mathrm{DoffKT}}Y_{\mathrm{K}n} \tag{S35}$$

$$-\mathbf{n}\cdot\Gamma_{\mathrm{Y0}} = -k_{\mathrm{onKTt}}\left(1-Y_{\mathrm{K}n}/Y_{\mathrm{K}}^{\max}\right)Y_0 \tag{S36}$$

$$-\mathbf{n}\cdot\Gamma_{\mathrm{Y00}} = -k_{\mathrm{onKTt}}\left(1-Y_{\mathrm{K}n}/Y_{\mathrm{K}}^{\max}\right)Y_{00} + k_{\mathrm{offKT}}Y_{\mathrm{K}n} \tag{S37}$$

In the above boundary conditions, **n** refers to the unit vector normal to the kinetochore boundary. Γ denotes the flux of proteins across the kinetochore boundary, with the protein species labeled by the subscript. The term $\left(1-Y_{\mathrm{K}n}/Y_{\mathrm{K}}^{\max}\right)$ sets a saturating limit of kinetochore-bound proteins. At the boundary of unattached kinetochores, the boundary condition (Eqs. (S30)-(S33)) characterizes the strong recruitment of SAC proteins (k_{onKTu}) and their turnover as transport-inactive species (k_{offKT}). At the boundary of attached kinetochores, the boundary condition (Eqs. (S34)-(S37)) characterizes the weak recruitment of SAC proteins ($k_{\mathrm{onKTt}} \gg k_{\mathrm{onKTu}}$) and issuance of transport-active proteins (k_{DoffKT}).

Eqs. (S21)-(S37) govern the spindle-mediated spatiotemporal dynamics of SAC proteins, and predict the nonlinear increase of the concentration of SAC proteins at the spindle pole as kinetochores get attached consecutively (**Figure 5A**). The predicted accumulation dynamics of SAC proteins at the spindle pole provides the foundation for the extended model below. The parameters for the core model are listed in **Supplementary Table 1**.

Extended model for coupled spatial and biochemical dynamics of SAC

To mimic the dynamics of SAC silencing, a biochemical circuit of the SAC components was added to the core model [1] (**Supplementary Figure 9**). The biochemical circuit here is rather simplified and captures just key interactions among several key molecular players. Most importantly, SAC silencing is assumed to be triggered from the spindle pole by a threshold concentration of SAC proteins. The actual trigger is not necessarily one of the SAC proteins, but can be any protein undergoing the same spatiotemporal dynamics. The model has assumed cyclin B to be the trigger since cyclin B undergoes the same kinetochore recruitment and poleward streaming [9, 10] and cyclin B was suggested to

promote its own degradation [11-13]. To implement the trigger event, the model assumes a generic toggle switch pathway localized at the spindle pole to be switched on by the threshold cyclin B concentration and initiate SAC silencing throughout the cell. Note that our current model captures the essential feature of SAC silencing being triggered by a threshold spindle pole signal. However, further details of the biochemical circuit, including parameter values, warrant future investigations, which will enhance the accuracy of the model.

Our biochemical circuit includes the following key SAC components: APC/C (A), SAC/MCC (M) and Cyclin B (C) (**Supplementary Figure 9**). In addition, the spindle pole cyclin B level triggers SAC silencing through a generic trigger factor, *X*, which is assumed to be concentrated at the spindle pole with a constant concentration and controlled by a self-activation-mediated toggle switch circuit. ***Metaphase duration*** is the time between when the final kinetochore gets attached and when the cyclin B level in the whole cell drops to below 50% of its initial level in 2N and 4N RPE-1 cells and below 10% of its initial level in DLD-1 2N/4N clones. The threshold cyclin B is chosen differently between clones deriving from the two cell lines to make the predicted metaphase durations fall in the range of the observed data. As all the clones deriving from the same ancestor assume the same value, the predicted metaphase duration can be compared among these clones. In fact, RPE-1 cells express Cyclin B at a significantly lower level than DLD-1 cells do (data not shown). This supports our assumption that compared to DLD-1 cells, RPE-1 cells require less relative reduction in Cyclin B to initiate anaphase.

Taken together, ***the equations for A, M and C repeat exactly the same spatiotemporal terms in Eqs. (S21)-(S37), and the same biochemical reaction terms for the same protein species in different compartments of the cell***. The entire set of PDEs is as reported in [1, 2] and are included below for readers' convenience. The superscripts a and i stand for the chemically active and inactive states, respectively. The total chemically active concentrations of each SAC pathway component in the cytoplasm and at the spindle pole read as $M_{\mathrm{t}}^{\mathrm{a}} = M_0^{\mathrm{a}} + M_1^{\mathrm{a}} + M_{00}^{\mathrm{a}} \left(+ M_{\mathrm{P}}^{\mathrm{a}} \right)$, $A_{\mathrm{t}}^{\mathrm{a}} = A_0^{\mathrm{a}} + A_1^{\mathrm{a}} + A_{00}^{\mathrm{a}} \left(+ A_{\mathrm{P}}^{\mathrm{a}} \right)$, $C_{\mathrm{t}}^{\mathrm{a}} = C_0^{\mathrm{a}} + C_1^{\mathrm{a}} + C_{00}^{\mathrm{a}} \left(+ C_{\mathrm{P}}^{\mathrm{a}} \right)$, $X_{\mathrm{t}}^{\mathrm{a}} = X_{00}^{\mathrm{a}} \left(+ X_{\mathrm{P}}^{\mathrm{a}} \right)$ (terms in brackets only exist in the spindle pole domain).

SAC/MCC in the cytoplasm:

$$\begin{aligned} \frac{\partial M_0^{\mathrm{a}}}{\partial t} &= D_{\mathrm{Dyn}} \nabla^2 M_0^{\mathrm{a}} + D_{\mathrm{Dyn}} \frac{\nabla U_{\mathrm{ext}}}{k_{\mathrm{B}} T} \cdot \nabla M_0^{\mathrm{a}} - k_{\mathrm{onMT}} \left(\rho_{\mathrm{MT}} \right) M_0^{\mathrm{a}} + k_{\mathrm{offMT}} M_1^{\mathrm{a}} \\ &\quad - k_{\mathrm{dMwA}} A_{\mathrm{t}}^{\mathrm{a}} M_0^{\mathrm{a}} + \left(k_{\mathrm{aMKT}} \left(t \right) + k_{\mathrm{aMCat}} \left(t; \tau_{\mathrm{DaM}} \right) C_{\mathrm{t}}^{\mathrm{a}} \right) M_0^{\mathrm{i}} \end{aligned} \tag{S38}$$

$$\frac{\partial M_0^{\mathrm{i}}}{\partial t} = D_{\mathrm{Dyn}} \nabla^2 M_0^{\mathrm{i}} + D_{\mathrm{Dyn}} \frac{\nabla U_{\mathrm{ext}}}{k_{\mathrm{B}} T} \cdot \nabla M_0^{\mathrm{i}} - k_{\mathrm{onMT}} \left(\rho_{\mathrm{MT}} \right) M_0^{\mathrm{i}} + k_{\mathrm{offMT}} M_1^{\mathrm{i}} + k_{\mathrm{dMwA}} A_{\mathrm{t}}^{\mathrm{a}} M_0^{\mathrm{a}} - \left(k_{\mathrm{aMKT}} \left(t \right) + k_{\mathrm{aMCat}} \left(t; \tau_{\mathrm{DaM}} \right) C_{\mathrm{t}}^{\mathrm{a}} \right) M_0^{\mathrm{i}} \tag{S39}$$

$$\frac{\partial M_1^{\mathrm{a}}}{\partial t} = D_{\mathrm{MT}} \nabla^2 M_1^{\mathrm{a}} - V \left(-\tilde{\mathbf{r}}^{\mathrm{SP}} \middle/ \left| \tilde{\mathbf{r}}^{\mathrm{SP}} \right| \right) \cdot \nabla M_1^{\mathrm{a}} + k_{\mathrm{onMT}} \left(\rho_{\mathrm{MT}} \right) M_0^{\mathrm{a}} - k_{\mathrm{offMT}} M_1^{\mathrm{a}} - k_{\mathrm{dMwA}} A_{\mathrm{t}}^{\mathrm{a}} M_1^{\mathrm{a}} + \left(k_{\mathrm{aMKT}} \left(t \right) + k_{\mathrm{aMCat}} \left(t; \tau_{\mathrm{DaM}} \right) C_{\mathrm{t}}^{\mathrm{a}} \right) M_1^{\mathrm{i}} \tag{S40}$$

$$\frac{\partial M_1^{\mathrm{i}}}{\partial t} = D_{\mathrm{MT}} \nabla^2 M_1^{\mathrm{i}} - V \left(-\tilde{\mathbf{r}}^{\mathrm{SP}} \middle/ \left| \tilde{\mathbf{r}}^{\mathrm{SP}} \right| \right) \cdot \nabla M_1^{\mathrm{i}} + k_{\mathrm{onMT}} \left(\rho_{\mathrm{MT}} \right) M_0^{\mathrm{i}} - k_{\mathrm{offMT}} M_1^{\mathrm{i}} + k_{\mathrm{dMwA}} A_{\mathrm{t}}^{\mathrm{a}} M_1^{\mathrm{a}} - \left(k_{\mathrm{aMKT}} \left(t \right) + k_{\mathrm{aMCat}} \left(t; \tau_{\mathrm{DaM}} \right) C_{\mathrm{t}}^{\mathrm{a}} \right) M_1^{\mathrm{i}} \tag{S41}$$

$$\frac{\partial M_{00}^{\mathrm{a}}}{\partial t} = D_{\mathrm{M}} \nabla^2 M_{00}^{\mathrm{a}} - k_{\mathrm{dMwA}} A_{\mathrm{t}}^{\mathrm{a}} M_{00}^{\mathrm{a}} + \left(k_{\mathrm{aMKT}} \left(t \right) + k_{\mathrm{aMCat}} \left(t; \tau_{\mathrm{DaM}} \right) C_{\mathrm{t}}^{\mathrm{a}} \right) M_{00}^{\mathrm{i}} \tag{S42}$$

$$\frac{\partial M_{00}^{\mathrm{i}}}{\partial t} = D_{\mathrm{M}} \nabla^2 M_{00}^{\mathrm{i}} + k_{\mathrm{dMwA}} A_{\mathrm{t}}^{\mathrm{a}} M_{00}^{\mathrm{a}} - \left(k_{\mathrm{aMKT}} \left(t \right) + k_{\mathrm{aMCat}} \left(t; \tau_{\mathrm{DaM}} \right) C_{\mathrm{t}}^{\mathrm{a}} \right) M_{00}^{\mathrm{i}} \tag{S43}$$

APC/C in the cytoplasm:

$$\frac{\partial A_0^{\mathrm{a}}}{\partial t} = D_{\mathrm{Dyn}} \nabla^2 A_0^{\mathrm{a}} + D_{\mathrm{Dyn}} \frac{\nabla U_{\mathrm{ext}}}{k_{\mathrm{B}} T} \cdot \nabla A_0^{\mathrm{a}} - k_{\mathrm{onMT}} \left(\rho_{\mathrm{MT}} \right) A_0^{\mathrm{a}} + k_{\mathrm{offMT}} A_1^{\mathrm{a}} - k_{\mathrm{dAwM}} M_{\mathrm{t}}^{\mathrm{a}} A_0^{\mathrm{a}} + k_{\mathrm{aAwC}} C_{\mathrm{t}}^{\mathrm{a}} A_0^{\mathrm{i}} + k_{\mathrm{aAwX}} X_{\mathrm{t}}^{\mathrm{a}} A_0^{\mathrm{i}} \tag{S44}$$

$$\frac{\partial A_0^{\mathrm{i}}}{\partial t} = D_{\mathrm{Dyn}} \nabla^2 A_0^{\mathrm{i}} + D_{\mathrm{Dyn}} \frac{\nabla U_{\mathrm{ext}}}{k_{\mathrm{B}} T} \cdot \nabla A_0^{\mathrm{i}} - k_{\mathrm{onMT}} \left(\rho_{\mathrm{MT}} \right) A_0^{\mathrm{i}} + k_{\mathrm{offMT}} A_1^{\mathrm{i}} + k_{\mathrm{dAwM}} M_{\mathrm{t}}^{\mathrm{a}} A_0^{\mathrm{a}} - k_{\mathrm{aAwC}} C_{\mathrm{t}}^{\mathrm{a}} A_0^{\mathrm{i}} - k_{\mathrm{aAwX}} X_{\mathrm{t}}^{\mathrm{a}} A_0^{\mathrm{i}} \tag{S45}$$

$$\frac{\partial A_1^{\mathrm{a}}}{\partial t} = D_{\mathrm{MT}} \nabla^2 A_1^{\mathrm{a}} - V \left(-\tilde{\mathbf{r}}^{\mathrm{SP}} \middle/ \left| \tilde{\mathbf{r}}^{\mathrm{SP}} \right| \right) \cdot \nabla A_1^{\mathrm{a}} + k_{\mathrm{onMT}} \left(\rho_{\mathrm{MT}} \right) A_0^{\mathrm{a}} - k_{\mathrm{offMT}} A_1^{\mathrm{a}} - k_{\mathrm{dAwM}} M_{\mathrm{t}}^{\mathrm{a}} A_1^{\mathrm{a}} + k_{\mathrm{aAwC}} C_{\mathrm{t}}^{\mathrm{a}} A_1^{\mathrm{i}} + k_{\mathrm{aAwX}} X_{\mathrm{t}}^{\mathrm{a}} A_1^{\mathrm{i}} \tag{S46}$$

$$\frac{\partial A_1^{\mathrm{i}}}{\partial t} = D_{\mathrm{MT}} \nabla^2 A_1^{\mathrm{i}} - V \left(-\tilde{\mathbf{r}}^{\mathrm{SP}} \middle/ \left| \tilde{\mathbf{r}}^{\mathrm{SP}} \right| \right) \cdot \nabla A_1^{\mathrm{i}} + k_{\mathrm{onMT}} \left(\rho_{\mathrm{MT}} \right) A_0^{\mathrm{i}} - k_{\mathrm{offMT}} A_1^{\mathrm{i}} + k_{\mathrm{dAwM}} M_{\mathrm{t}}^{\mathrm{a}} A_1^{\mathrm{a}} - k_{\mathrm{aAwC}} C_{\mathrm{t}}^{\mathrm{a}} A_1^{\mathrm{i}} - k_{\mathrm{aAwX}} X_{\mathrm{t}}^{\mathrm{a}} A_1^{\mathrm{i}} \tag{S47}$$

$$\frac{\partial A_{00}^{\mathrm{a}}}{\partial t} = D_{\mathrm{A}} \nabla^2 A_{00}^{\mathrm{a}} - k_{\mathrm{dAwM}} M_{\mathrm{t}}^{\mathrm{a}} A_{00}^{\mathrm{a}} + k_{\mathrm{aAwC}} C_{\mathrm{t}}^{\mathrm{a}} A_{00}^{\mathrm{i}} + k_{\mathrm{aAwX}} X_{\mathrm{t}}^{\mathrm{a}} A_{00}^{\mathrm{i}} \tag{S48}$$

$$\frac{\partial A_{00}^{\mathrm{i}}}{\partial t} = D_{\mathrm{A}}\nabla^2 A_{00}^{\mathrm{i}} + k_{\mathrm{dAwM}} M_{\mathrm{t}}^{\mathrm{a}} A_{00}^{\mathrm{a}} - k_{\mathrm{aAwC}} C_{\mathrm{t}}^{\mathrm{a}} A_{00}^{\mathrm{i}} - k_{\mathrm{aAwX}} X_{\mathrm{t}}^{\mathrm{a}} A_{00}^{\mathrm{i}} \tag{S49}$$

Cyclin B in the cytoplasm:

$$\begin{aligned}\frac{\partial C_0^{\mathrm{a}}}{\partial t} &= D_{\mathrm{Dyn}}\nabla^2 C_0^{\mathrm{a}} + D_{\mathrm{Dyn}}\frac{\nabla U_{\mathrm{ext}}}{k_{\mathrm{B}}T}\cdot\nabla C_0^{\mathrm{a}} - k_{\mathrm{onMT}}\left(\rho_{\mathrm{MT}}\right) C_0^{\mathrm{a}} + k_{\mathrm{offMT}} C_1^{\mathrm{a}} \\ &\quad - k_{\mathrm{dCwA}}\frac{\left(f_{\mathrm{A}}/K_{\mathrm{mdCwA}}\right)^H}{1+\left(f_{\mathrm{A}}/K_{\mathrm{mdCwA}}\right)^H} A_{\mathrm{t}}^{\mathrm{a}}\cdot C_0^{\mathrm{a}}\end{aligned} \tag{S50}$$

$$\begin{aligned}\frac{\partial C_1^{\mathrm{a}}}{\partial t} &= D_{\mathrm{MT}}\nabla^2 C_1^{\mathrm{a}} - V\left(-\tilde{\mathbf{r}}^{\mathrm{SP}}\big/\left|\tilde{\mathbf{r}}^{\mathrm{SP}}\right|\right)\cdot\nabla C_1^{\mathrm{a}} + k_{\mathrm{onMT}}\left(\rho_{\mathrm{MT}}\right) C_0^{\mathrm{a}} - k_{\mathrm{offMT}} C_1^{\mathrm{a}} \\ &\quad - k_{\mathrm{dCwA}}\frac{\left(f_{\mathrm{A}}/K_{\mathrm{mdCwA}}\right)^H}{1+\left(f_{\mathrm{A}}/K_{\mathrm{mdCwA}}\right)^H} A_{\mathrm{t}}^{\mathrm{a}}\cdot C_1^{\mathrm{a}}\end{aligned} \tag{S51}$$

$$\frac{\partial C_{00}^{\mathrm{a}}}{\partial t} = D_{\mathrm{C}}\nabla^2 C_{00}^{\mathrm{a}} - k_{\mathrm{dCwA}}\frac{\left(f_{\mathrm{A}}/K_{\mathrm{mdCwA}}\right)^H}{1+\left(f_{\mathrm{A}}/K_{\mathrm{mdCwA}}\right)^H} A_{\mathrm{t}}^{\mathrm{a}}\cdot C_{00}^{\mathrm{a}} + k_{\mathrm{sC}} \tag{S52}$$

where $f_{\mathrm{A}} = \left(A_0^{\mathrm{a}} + A_1^{\mathrm{a}} + A_{00}^{\mathrm{a}}\right)\big/\left(A_0^{\mathrm{a}} + A_1^{\mathrm{a}} + A_{00}^{\mathrm{a}} + A_0^{\mathrm{i}} + A_1^{\mathrm{i}} + A_{00}^{\mathrm{i}}\right)$.

Trigger factor in the cytoplasm (GK refers to the Goldbeter-Koshland function, a common function describing auto-activation):

$$\frac{\partial X_{00}^{\mathrm{a}}}{\partial t} = D_{\mathrm{X}}\nabla^2 X_{00}^{\mathrm{a}} - k_{\mathrm{dX}} X_{00}^{\mathrm{a}} + \left(k_{\mathrm{aXwC}} C_{\mathrm{t}}^{\mathrm{a}} + k_{\mathrm{aXwX}}\mathrm{GK}\left(k_1 X_{\mathrm{t}}^{\mathrm{a}}, k_2, J_1, J_2\right)\right) X_{00}^{\mathrm{i}} \tag{S53}$$

$$\frac{\partial X_{00}^{\mathrm{i}}}{\partial t} = D_{\mathrm{X}}\nabla^2 X_{00}^{\mathrm{i}} + k_{\mathrm{dX}} X_{00}^{\mathrm{a}} - \left(k_{\mathrm{aXwC}} C_{\mathrm{t}}^{\mathrm{a}} + k_{\mathrm{aXwX}}\mathrm{GK}\left(k_1 X_{\mathrm{t}}^{\mathrm{a}}, k_2, J_1, J_2\right)\right) X_{00}^{\mathrm{i}} \tag{S54}$$

SAC/MCC at the spindle pole:

$$\begin{aligned}\frac{\partial M_0^{\mathrm{a}}}{\partial t} &= D_{\mathrm{Dyn}}\nabla^2 M_0^{\mathrm{a}} - k_{\mathrm{onMT}}^{\mathrm{SP}} M_0^{\mathrm{a}} + k_{\mathrm{offMT}}^{\mathrm{SP}} M_1^{\mathrm{a}} - k_{\mathrm{onSP}} M_0^{\mathrm{a}} + k_{\mathrm{offSP}} M_{\mathrm{P}}^{\mathrm{a}} \\ &\quad - k_{\mathrm{dMwA}} A_{\mathrm{t}}^{\mathrm{a}} M_0^{\mathrm{a}} + \left(k_{\mathrm{aMKT}}\left(t\right) + k_{\mathrm{aMCat}}\left(t;\tau_{\mathrm{DaM}}\right) C_{\mathrm{t}}^{\mathrm{a}}\right) M_0^{\mathrm{i}}\end{aligned} \tag{S55}$$

$$\frac{\partial M_0^{\mathrm{i}}}{\partial t} = D_{\mathrm{Dyn}} \nabla^2 M_0^{\mathrm{i}} - k_{\mathrm{onMT}}^{\mathrm{SP}} M_0^{\mathrm{i}} + k_{\mathrm{offMT}}^{\mathrm{SP}} M_1^{\mathrm{i}} - k_{\mathrm{onSP}} M_0^{\mathrm{i}} + k_{\mathrm{offSP}} M_{\mathrm{P}}^{\mathrm{i}} + k_{\mathrm{dMwA}} A_{\mathrm{t}}^{\mathrm{a}} M_0^{\mathrm{a}} - \left(k_{\mathrm{aMKT}}(t) + k_{\mathrm{aMCat}}(t;\tau_{\mathrm{DaM}}) C_{\mathrm{t}}^{\mathrm{a}}\right) M_0^{\mathrm{i}} \tag{S56}$$

$$\frac{\partial M_1^{\mathrm{a}}}{\partial t} = D_{\mathrm{MT}} \nabla^2 M_1^{\mathrm{a}} - V\left(-\tilde{\mathbf{r}}^{\mathrm{SP}} / \left|\tilde{\mathbf{r}}^{\mathrm{SP}}\right|\right) \cdot \nabla M_1^{\mathrm{a}} + k_{\mathrm{onMT}}^{\mathrm{SP}} M_0^{\mathrm{a}} - k_{\mathrm{offMT}}^{\mathrm{SP}} M_1^{\mathrm{a}} - k_{\mathrm{onSP}} M_1^{\mathrm{a}} - k_{\mathrm{dMwA}} A_{\mathrm{t}}^{\mathrm{a}} M_1^{\mathrm{a}} + \left(k_{\mathrm{aMKT}}(t) + k_{\mathrm{aMCat}}(t;\tau_{\mathrm{DaM}}) C_{\mathrm{t}}^{\mathrm{a}}\right) M_1^{\mathrm{i}} \tag{S57}$$

$$\frac{\partial M_1^{\mathrm{i}}}{\partial t} = D_{\mathrm{MT}} \nabla^2 M_1^{\mathrm{i}} - V\left(-\tilde{\mathbf{r}}^{\mathrm{SP}} / \left|\tilde{\mathbf{r}}^{\mathrm{SP}}\right|\right) \cdot \nabla M_1^{\mathrm{i}} + k_{\mathrm{onMT}}^{\mathrm{SP}} M_0^{\mathrm{i}} - k_{\mathrm{offMT}}^{\mathrm{SP}} M_1^{\mathrm{i}} - k_{\mathrm{onSP}} M_1^{\mathrm{i}} + k_{\mathrm{dMwA}} A_{\mathrm{t}}^{\mathrm{a}} M_1^{\mathrm{a}} - \left(k_{\mathrm{aMKT}}(t) + k_{\mathrm{aMCat}}(t;\tau_{\mathrm{DaM}}) C_{\mathrm{t}}^{\mathrm{a}}\right) M_1^{\mathrm{i}} \tag{S58}$$

$$\frac{\partial M_{\mathrm{P}}^{\mathrm{a}}}{\partial t} = D_{\mathrm{P}} \nabla^2 M_{\mathrm{P}}^{\mathrm{a}} + k_{\mathrm{onSP}} \left(M_0^{\mathrm{a}} + M_1^{\mathrm{a}}\right) - k_{\mathrm{offSP}} M_{\mathrm{P}}^{\mathrm{a}} - k_{\mathrm{dMwA}} A_{\mathrm{t}}^{\mathrm{a}} M_{\mathrm{P}}^{\mathrm{a}} + \left(k_{\mathrm{aMKT}}(t) + k_{\mathrm{aMCat}}(t;\tau_{\mathrm{DaM}}) C_{\mathrm{t}}^{\mathrm{a}}\right) M_{\mathrm{P}}^{\mathrm{i}} \tag{S59}$$

$$\frac{\partial M_{\mathrm{P}}^{\mathrm{i}}}{\partial t} = D_{\mathrm{P}} \nabla^2 M_{\mathrm{P}}^{\mathrm{i}} + k_{\mathrm{onSP}} \left(M_0^{\mathrm{i}} + M_1^{\mathrm{i}}\right) - k_{\mathrm{offSP}} M_{\mathrm{P}}^{\mathrm{i}} + k_{\mathrm{dMwA}} A_{\mathrm{t}}^{\mathrm{a}} M_{\mathrm{P}}^{\mathrm{a}} - \left(k_{\mathrm{aMKT}}(t) + k_{\mathrm{aMCat}}(t;\tau_{\mathrm{DaM}}) C_{\mathrm{t}}^{\mathrm{a}}\right) M_{\mathrm{P}}^{\mathrm{i}} \tag{S60}$$

$$\frac{\partial M_{00}^{\mathrm{a}}}{\partial t} = D_{\mathrm{M}} \nabla^2 M_{00}^{\mathrm{a}} - k_{\mathrm{dMwA}} A_{\mathrm{t}}^{\mathrm{a}} M_{00}^{\mathrm{a}} + \left(k_{\mathrm{aMKT}}(t) + k_{\mathrm{aMCat}}(t;\tau_{\mathrm{DaM}}) C_{\mathrm{t}}^{\mathrm{a}}\right) M_{00}^{\mathrm{i}} \tag{S61}$$

$$\frac{\partial M_{00}^{\mathrm{i}}}{\partial t} = D_{\mathrm{M}} \nabla^2 M_{00}^{\mathrm{i}} + k_{\mathrm{dMwA}} A_{\mathrm{t}}^{\mathrm{a}} M_{00}^{\mathrm{a}} - \left(k_{\mathrm{aMKT}}(t) + k_{\mathrm{aMCat}}(t;\tau_{\mathrm{DaM}}) C_{\mathrm{t}}^{\mathrm{a}}\right) M_{00}^{\mathrm{i}} \tag{S62}$$

APC/C at the spindle pole:

$$\frac{\partial A_0^{\mathrm{a}}}{\partial t} = D_{\mathrm{Dyn}} \nabla^2 A_0^{\mathrm{a}} - k_{\mathrm{onMT}}^{\mathrm{SP}} A_0^{\mathrm{a}} + k_{\mathrm{offMT}}^{\mathrm{SP}} A_1^{\mathrm{a}} - k_{\mathrm{onSP}} A_0^{\mathrm{a}} + k_{\mathrm{offSP}} A_{\mathrm{P}}^{\mathrm{a}} - k_{\mathrm{dAwM}} M_{\mathrm{t}}^{\mathrm{a}} A_0^{\mathrm{a}} + k_{\mathrm{aAwC}} C_{\mathrm{t}}^{\mathrm{a}} A_0^{\mathrm{i}} + k_{\mathrm{aAwX}} X_{\mathrm{t}}^{\mathrm{a}} A_0^{\mathrm{i}} \tag{S63}$$

$$\frac{\partial A_0^{\mathrm{i}}}{\partial t} = D_{\mathrm{Dyn}} \nabla^2 A_0^{\mathrm{i}} - k_{\mathrm{onMT}}^{\mathrm{SP}} A_0^{\mathrm{i}} + k_{\mathrm{offMT}}^{\mathrm{SP}} A_1^{\mathrm{i}} - k_{\mathrm{onSP}} A_0^{\mathrm{i}} + k_{\mathrm{offSP}} A_{\mathrm{P}}^{\mathrm{i}} + k_{\mathrm{dAwM}} M_{\mathrm{t}}^{\mathrm{a}} A_0^{\mathrm{a}} - k_{\mathrm{aAwC}} C_{\mathrm{t}}^{\mathrm{a}} A_0^{\mathrm{i}} - k_{\mathrm{aAwX}} X_{\mathrm{t}}^{\mathrm{a}} A_0^{\mathrm{i}} \tag{S64}$$

$$\frac{\partial A_1^{\mathrm{a}}}{\partial t} = D_{\mathrm{MT}} \nabla^2 A_1^{\mathrm{a}} - V\left(-\tilde{\mathbf{r}}^{\mathrm{SP}} / \left|\tilde{\mathbf{r}}^{\mathrm{SP}}\right|\right) \cdot \nabla A_1^{\mathrm{a}} + k_{\mathrm{onMT}}^{\mathrm{SP}} A_0^{\mathrm{a}} - k_{\mathrm{offMT}}^{\mathrm{SP}} A_1^{\mathrm{a}} - k_{\mathrm{onSP}} A_1^{\mathrm{a}} - k_{\mathrm{dAwM}} M_{\mathrm{t}}^{\mathrm{a}} A_1^{\mathrm{a}} + k_{\mathrm{aAwC}} C_{\mathrm{t}}^{\mathrm{a}} A_1^{\mathrm{i}} + k_{\mathrm{aAwX}} X_{\mathrm{t}}^{\mathrm{a}} A_1^{\mathrm{i}} \tag{S65}$$

$$\frac{\partial A_1^{\mathrm{i}}}{\partial t} = D_{\mathrm{MT}}\nabla^2 A_1^{\mathrm{i}} - V\left(-\tilde{\mathbf{r}}^{\mathrm{SP}}\Big/\left|\tilde{\mathbf{r}}^{\mathrm{SP}}\right|\right)\cdot\nabla A_1^{\mathrm{i}} + k_{\mathrm{onMT}}^{\mathrm{SP}} A_0^{\mathrm{i}} - k_{\mathrm{offMT}}^{\mathrm{SP}} A_1^{\mathrm{i}} - k_{\mathrm{onSP}} A_1^{\mathrm{i}} + k_{\mathrm{dAwM}} M_{\mathrm{t}}^{\mathrm{a}} A_1^{\mathrm{a}} - k_{\mathrm{aAwC}} C_{\mathrm{t}}^{\mathrm{a}} A_1^{\mathrm{i}} - k_{\mathrm{aAwX}} X_{\mathrm{t}}^{\mathrm{a}} A_1^{\mathrm{i}} \quad \text{(S66)}$$

$$\frac{\partial A_{\mathrm{P}}^{\mathrm{a}}}{\partial t} = D_{\mathrm{P}}\nabla^2 A_{\mathrm{P}}^{\mathrm{a}} + k_{\mathrm{onSP}}\left(A_0^{\mathrm{a}} + A_1^{\mathrm{a}}\right) - k_{\mathrm{offSP}} A_{\mathrm{P}}^{\mathrm{a}} - k_{\mathrm{dAwM}} M_{\mathrm{t}}^{\mathrm{a}} A_{\mathrm{P}}^{\mathrm{a}} + k_{\mathrm{aAwC}} C_{\mathrm{t}}^{\mathrm{a}} A_{\mathrm{P}}^{\mathrm{i}} + k_{\mathrm{aAwX}} X_{\mathrm{t}}^{\mathrm{a}} A_{\mathrm{P}}^{\mathrm{i}} \quad \text{(S67)}$$

$$\frac{\partial A_{\mathrm{P}}^{\mathrm{i}}}{\partial t} = D_{\mathrm{P}}\nabla^2 A_{\mathrm{P}}^{\mathrm{i}} + k_{\mathrm{onSP}}\left(A_0^{\mathrm{i}} + A_1^{\mathrm{i}}\right) - k_{\mathrm{offSP}} A_{\mathrm{P}}^{\mathrm{i}} + k_{\mathrm{dAwM}} M_{\mathrm{t}}^{\mathrm{a}} A_{\mathrm{P}}^{\mathrm{a}} - k_{\mathrm{aAwC}} C_{\mathrm{t}}^{\mathrm{a}} A_{\mathrm{P}}^{\mathrm{i}} - k_{\mathrm{aAwX}} X_{\mathrm{t}}^{\mathrm{a}} A_{\mathrm{P}}^{\mathrm{i}} \quad \text{(S68)}$$

$$\frac{\partial A_{00}^{\mathrm{a}}}{\partial t} = D_{\mathrm{A}}\nabla^2 A_{00}^{\mathrm{a}} - k_{\mathrm{dAwM}} M_{\mathrm{t}}^{\mathrm{a}} A_{00}^{\mathrm{a}} + k_{\mathrm{aAwC}} C_{\mathrm{t}}^{\mathrm{a}} A_{00}^{\mathrm{i}} + k_{\mathrm{aAwX}} X_{\mathrm{t}}^{\mathrm{a}} A_{00}^{\mathrm{i}} \quad \text{(S69)}$$

$$\frac{\partial A_{00}^{\mathrm{i}}}{\partial t} = D_{\mathrm{A}}\nabla^2 A_{00}^{\mathrm{i}} + k_{\mathrm{dAwM}} M_{\mathrm{t}}^{\mathrm{a}} A_{00}^{\mathrm{a}} - k_{\mathrm{aAwC}} C_{\mathrm{t}}^{\mathrm{a}} A_{00}^{\mathrm{i}} - k_{\mathrm{aAwX}} X_{\mathrm{t}}^{\mathrm{a}} A_{00}^{\mathrm{i}} \quad \text{(S70)}$$

Cyclin B at the spindle pole:

$$\frac{\partial C_0^{\mathrm{a}}}{\partial t} = D_{\mathrm{Dyn}}\nabla^2 C_0^{\mathrm{a}} - k_{\mathrm{onMT}}^{\mathrm{SP}} C_0^{\mathrm{a}} + k_{\mathrm{offMT}}^{\mathrm{SP}} C_1^{\mathrm{a}} - k_{\mathrm{onSP}} C_0^{\mathrm{a}} + k_{\mathrm{offSP}} C_{\mathrm{P}}^{\mathrm{a}} - k_{\mathrm{dCwA}} \frac{\left(f_{\mathrm{A}}/K_{\mathrm{mdCwA}}\right)^H}{1+\left(f_{\mathrm{A}}/K_{\mathrm{mdCwA}}\right)^H} A_{\mathrm{t}}^{\mathrm{a}} \cdot C_0^{\mathrm{a}} \quad \text{(S71)}$$

$$\frac{\partial C_1^{\mathrm{a}}}{\partial t} = D_{\mathrm{MT}}\nabla^2 C_1^{\mathrm{a}} - V\left(-\tilde{\mathbf{r}}^{\mathrm{SP}}\Big/\left|\tilde{\mathbf{r}}^{\mathrm{SP}}\right|\right)\cdot\nabla C_1^{\mathrm{a}} + k_{\mathrm{onMT}}^{\mathrm{SP}} C_0^{\mathrm{a}} - k_{\mathrm{offMT}}^{\mathrm{SP}} C_1^{\mathrm{a}} - k_{\mathrm{onSP}} C_1^{\mathrm{a}} - k_{\mathrm{dCwA}} \frac{\left(f_{\mathrm{A}}/K_{\mathrm{mdCwA}}\right)^H}{1+\left(f_{\mathrm{A}}/K_{\mathrm{mdCwA}}\right)^H} A_{\mathrm{t}}^{\mathrm{a}} \cdot C_1^{\mathrm{a}} \quad \text{(S72)}$$

$$\frac{\partial C_{\mathrm{P}}^{\mathrm{a}}}{\partial t} = D_{\mathrm{C}}\nabla^2 C_{\mathrm{P}}^{\mathrm{a}} + k_{\mathrm{onSP}}\left(C_0^{\mathrm{a}} + C_1^{\mathrm{a}}\right) - k_{\mathrm{offSP}} C_{\mathrm{P}}^{\mathrm{a}} - k_{\mathrm{dCwA}} \frac{\left(f_{\mathrm{A}}/K_{\mathrm{mdCwA}}\right)^H}{1+\left(f_{\mathrm{A}}/K_{\mathrm{mdCwA}}\right)^H} A_{\mathrm{t}}^{\mathrm{a}} \cdot C_{\mathrm{P}}^{\mathrm{a}} \quad \text{(S73)}$$

$$\frac{\partial C_{00}^{\mathrm{a}}}{\partial t} = D_{\mathrm{C}}\nabla^2 C_{00}^{\mathrm{a}} - k_{\mathrm{dCwA}} \frac{\left(f_{\mathrm{A}}/K_{\mathrm{mdCwA}}\right)^H}{1+\left(f_{\mathrm{A}}/K_{\mathrm{mdCwA}}\right)^H} A_{\mathrm{t}}^{\mathrm{a}} \cdot C_{00}^{\mathrm{a}} \quad \text{(S74)}$$

where $f_{\mathrm{A}} = \left(A_0^{\mathrm{a}} + A_1^{\mathrm{a}} + A_{00}^{\mathrm{a}} + A_{\mathrm{P}}^{\mathrm{a}}\right)\Big/\left(A_0^{\mathrm{a}} + A_1^{\mathrm{a}} + A_{00}^{\mathrm{a}} + A_{\mathrm{P}}^{\mathrm{a}} + A_0^{\mathrm{i}} + A_1^{\mathrm{i}} + A_{00}^{\mathrm{i}} + A_{\mathrm{P}}^{\mathrm{i}}\right)$.

Trigger factor at the spindle pole:

$$\frac{\partial X_{00}^{\mathrm{a}}}{\partial t} = D_{\mathrm{X}} \nabla^2 X_{00}^{\mathrm{a}} - k_{\mathrm{onSPX}} X_{00}^{\mathrm{a}} + k_{\mathrm{offSPX}} X_{\mathrm{P}}^{\mathrm{a}} - k_{\mathrm{dX}} X_{00}^{\mathrm{a}} + \left(k_{\mathrm{aXwC}} C_{\mathrm{t}}^{\mathrm{a}} + k_{\mathrm{aXwX}} \mathrm{GK}\left(k_1 X_{\mathrm{t}}^{\mathrm{a}}, k_2, J_1, J_2 \right) \right) X_{00}^{\mathrm{i}} \tag{S75}$$

$$\frac{\partial X_{00}^{\mathrm{i}}}{\partial t} = D_{\mathrm{X}} \nabla^2 X_{00}^{\mathrm{i}} - k_{\mathrm{onSPX}} X_{00}^{\mathrm{i}} + k_{\mathrm{offSPX}} X_{\mathrm{P}}^{\mathrm{i}} + k_{\mathrm{dX}} X_{00}^{\mathrm{a}} - \left(k_{\mathrm{aXwC}} C_{\mathrm{t}}^{\mathrm{a}} + k_{\mathrm{aXwX}} \mathrm{GK}\left(k_1 X_{\mathrm{t}}^{\mathrm{a}}, k_2, J_1, J_2 \right) \right) X_{00}^{\mathrm{i}} \tag{S76}$$

$$\frac{\partial X_{\mathrm{P}}^{\mathrm{a}}}{\partial t} = D_{\mathrm{P}} \nabla^2 X_{\mathrm{P}}^{\mathrm{a}} + k_{\mathrm{onSPX}} X_{00}^{\mathrm{a}} - k_{\mathrm{offSPX}} X_{\mathrm{P}}^{\mathrm{a}} - k_{\mathrm{dX}} X_{\mathrm{P}}^{\mathrm{a}} + \left(k_{\mathrm{aXwC}} C_{\mathrm{t}}^{\mathrm{a}} + k_{\mathrm{aXwX}} \mathrm{GK}\left(k_1 X_{\mathrm{t}}^{\mathrm{a}}, k_2, J_1, J_2 \right) \right) X_{\mathrm{P}}^{\mathrm{i}} \tag{S77}$$

$$\frac{\partial X_{\mathrm{P}}^{\mathrm{i}}}{\partial t} = D_{\mathrm{P}} \nabla^2 X_{\mathrm{P}}^{\mathrm{i}} + k_{\mathrm{onSPX}} X_{00}^{\mathrm{i}} - k_{\mathrm{offSPX}} X_{\mathrm{P}}^{\mathrm{i}} + k_{\mathrm{dX}} X_{\mathrm{P}}^{\mathrm{a}} - \left(k_{\mathrm{aXwC}} C_{\mathrm{t}}^{\mathrm{a}} + k_{\mathrm{aXwX}} \mathrm{GK}\left(k_1 X_{\mathrm{t}}^{\mathrm{a}}, k_2, J_1, J_2 \right) \right) X_{\mathrm{P}}^{\mathrm{i}} \tag{S78}$$

SAC/MCC at the n-th kinetochore:

$$\frac{\partial M_{\mathrm{K}n}^{\mathrm{a}}}{\partial t} = D_{\mathrm{K}} \nabla^2 M_{\mathrm{K}n}^{\mathrm{a}} - k_{\mathrm{dMwA}} A_{\mathrm{K}n}^{\mathrm{a}} M_{\mathrm{K}n}^{\mathrm{a}} + \left(k_{\mathrm{aMKT}}(t) + k_{\mathrm{aMCat}}(t; \tau_{\mathrm{DaM}}) C_{\mathrm{K}n}^{\mathrm{a}} \right) M_{\mathrm{K}n}^{\mathrm{i}} \tag{S79}$$

$$\frac{\partial M_{\mathrm{K}n}^{\mathrm{i}}}{\partial t} = D_{\mathrm{K}} \nabla^2 M_{\mathrm{K}n}^{\mathrm{i}} + k_{\mathrm{dMwA}} A_{\mathrm{K}n}^{\mathrm{a}} M_{\mathrm{K}n}^{\mathrm{a}} - \left(k_{\mathrm{aMKT}}(t) + k_{\mathrm{aMCat}}(t; \tau_{\mathrm{DaM}}) C_{\mathrm{K}n}^{\mathrm{a}} \right) M_{\mathrm{K}n}^{\mathrm{i}} \tag{S80}$$

SAC/MCC flux across the boundary of unattached kinetochore:

$$-\mathbf{n} \cdot \mathbf{\Gamma}_{M_{\mathrm{K}n}^{\mathrm{a}}} = k_{\mathrm{onKTu}} \left(1 - \left(M_{\mathrm{K}n}^{\mathrm{a}} + M_{\mathrm{K}n}^{\mathrm{i}} \right) \Big/ M_{\mathrm{K}}^{\max} \right) \left(M_0^{\mathrm{a}} + M_1^{\mathrm{a}} + M_{00}^{\mathrm{a}} \right) - k_{\mathrm{offKT}} M_{\mathrm{K}n}^{\mathrm{a}} \tag{S81}$$

$$-\mathbf{n} \cdot \mathbf{\Gamma}_{M_{\mathrm{K}n}^{\mathrm{i}}} = k_{\mathrm{onKTu}} \left(1 - \left(M_{\mathrm{K}n}^{\mathrm{a}} + M_{\mathrm{K}n}^{\mathrm{i}} \right) \Big/ M_{\mathrm{K}}^{\max} \right) \left(M_0^{\mathrm{i}} + M_1^{\mathrm{i}} + M_{00}^{\mathrm{i}} \right) - k_{\mathrm{offKT}} M_{\mathrm{K}n}^{\mathrm{i}} \tag{S82}$$

$$-\mathbf{n} \cdot \mathbf{\Gamma}_{M_0^{\mathrm{a}}} = -k_{\mathrm{onKTu}} \left(1 - \left(M_{\mathrm{K}n}^{\mathrm{a}} + M_{\mathrm{K}n}^{\mathrm{i}} \right) \Big/ M_{\mathrm{K}}^{\max} \right) M_0^{\mathrm{a}} \tag{S83}$$

$$-\mathbf{n} \cdot \mathbf{\Gamma}_{M_0^{\mathrm{i}}} = -k_{\mathrm{onKTu}} \left(1 - \left(M_{\mathrm{K}n}^{\mathrm{a}} + M_{\mathrm{K}n}^{\mathrm{i}} \right) \Big/ M_{\mathrm{K}}^{\max} \right) M_0^{\mathrm{i}} \tag{S84}$$

$$-\mathbf{n}\cdot\mathbf{\Gamma}_{M_1^{\mathrm{a}}}=-k_{\mathrm{onKTu}}\left(1-\left(M_{\mathrm{K}n}^{\mathrm{a}}+M_{\mathrm{K}n}^{\mathrm{i}}\right)\Big/M_{\mathrm{K}}^{\max}\right)M_1^{\mathrm{a}} \tag{S85}$$

$$-\mathbf{n}\cdot\mathbf{\Gamma}_{M_1^{\mathrm{i}}}=-k_{\mathrm{onKTu}}\left(1-\left(M_{\mathrm{K}n}^{\mathrm{a}}+M_{\mathrm{K}n}^{\mathrm{i}}\right)\Big/M_{\mathrm{K}}^{\max}\right)M_1^{\mathrm{i}} \tag{S86}$$

$$-\mathbf{n}\cdot\mathbf{\Gamma}_{M_{00}^{\mathrm{a}}}=-k_{\mathrm{onKTu}}\left(1-\left(M_{\mathrm{K}n}^{\mathrm{a}}+M_{\mathrm{K}n}^{\mathrm{i}}\right)\Big/M_{\mathrm{K}}^{\max}\right)M_{00}^{\mathrm{a}}+k_{\mathrm{offKT}}M_{\mathrm{K}n}^{\mathrm{a}} \tag{S87}$$

$$-\mathbf{n}\cdot\mathbf{\Gamma}_{M_{00}^{\mathrm{i}}}=-k_{\mathrm{onKTu}}\left(1-\left(M_{\mathrm{K}n}^{\mathrm{a}}+M_{\mathrm{K}n}^{\mathrm{i}}\right)\Big/M_{\mathrm{K}}^{\max}\right)M_{00}^{\mathrm{i}}+k_{\mathrm{offKT}}M_{\mathrm{K}n}^{\mathrm{i}} \tag{S88}$$

SAC/MCC flux across the boundary of attached kinetochore:

$$-\mathbf{n}\cdot\mathbf{\Gamma}_{M_{\mathrm{K}n}^{\mathrm{a}}}=k_{\mathrm{onKTt}}\left(1-\left(M_{\mathrm{K}n}^{\mathrm{a}}+M_{\mathrm{K}n}^{\mathrm{i}}\right)\Big/M_{\mathrm{K}}^{\max}\right)\left(M_0^{\mathrm{a}}+M_1^{\mathrm{a}}+M_{00}^{\mathrm{a}}\right)-\left(k_{\mathrm{offKT}}+k_{\mathrm{DoffKT}}\right)M_{\mathrm{K}n}^{\mathrm{a}} \tag{S89}$$

$$-\mathbf{n}\cdot\mathbf{\Gamma}_{M_{\mathrm{K}n}^{\mathrm{i}}}=k_{\mathrm{onKTt}}\left(1-\left(M_{\mathrm{K}n}^{\mathrm{a}}+M_{\mathrm{K}n}^{\mathrm{i}}\right)\Big/M_{\mathrm{K}}^{\max}\right)\left(M_0^{\mathrm{i}}+M_1^{\mathrm{i}}+M_{00}^{\mathrm{i}}\right)-\left(k_{\mathrm{offKT}}+k_{\mathrm{DoffKT}}\right)M_{\mathrm{K}n}^{\mathrm{i}} \tag{S90}$$

$$-\mathbf{n}\cdot\mathbf{\Gamma}_{M_0^{\mathrm{a}}}=-k_{\mathrm{onKTt}}\left(1-\left(M_{\mathrm{K}n}^{\mathrm{a}}+M_{\mathrm{K}n}^{\mathrm{i}}\right)\Big/M_{\mathrm{K}}^{\max}\right)M_0^{\mathrm{a}} \tag{S91}$$

$$-\mathbf{n}\cdot\mathbf{\Gamma}_{M_0^{\mathrm{i}}}=-k_{\mathrm{onKTt}}\left(1-\left(M_{\mathrm{K}n}^{\mathrm{a}}+M_{\mathrm{K}n}^{\mathrm{i}}\right)\Big/M_{\mathrm{K}}^{\max}\right)M_0^{\mathrm{i}} \tag{S92}$$

$$-\mathbf{n}\cdot\mathbf{\Gamma}_{M_1^{\mathrm{a}}}=-k_{\mathrm{onKTt}}\left(1-\left(M_{\mathrm{K}n}^{\mathrm{a}}+M_{\mathrm{K}n}^{\mathrm{i}}\right)\Big/M_{\mathrm{K}}^{\max}\right)M_1^{\mathrm{a}}+k_{\mathrm{DoffKT}}M_{\mathrm{K}n}^{\mathrm{a}} \tag{S93}$$

$$-\mathbf{n}\cdot\mathbf{\Gamma}_{M_1^{\mathrm{i}}}=-k_{\mathrm{onKTt}}\left(1-\left(M_{\mathrm{K}n}^{\mathrm{a}}+M_{\mathrm{K}n}^{\mathrm{i}}\right)\Big/M_{\mathrm{K}}^{\max}\right)M_1^{\mathrm{i}}+k_{\mathrm{DoffKT}}M_{\mathrm{K}n}^{\mathrm{i}} \tag{S94}$$

$$-\mathbf{n}\cdot\mathbf{\Gamma}_{M_{00}^{\mathrm{a}}}=-k_{\mathrm{onKTt}}\left(1-\left(M_{\mathrm{K}n}^{\mathrm{a}}+M_{\mathrm{K}n}^{\mathrm{i}}\right)\Big/M_{\mathrm{K}}^{\max}\right)M_{00}^{\mathrm{a}}+k_{\mathrm{offKT}}M_{\mathrm{K}n}^{\mathrm{a}} \tag{S95}$$

$$-\mathbf{n}\cdot\mathbf{\Gamma}_{M_{00}^{\mathrm{i}}}=-k_{\mathrm{onKTt}}\left(1-\left(M_{\mathrm{K}n}^{\mathrm{a}}+M_{\mathrm{K}n}^{\mathrm{i}}\right)\Big/M_{\mathrm{K}}^{\max}\right)M_{00}^{\mathrm{i}}+k_{\mathrm{offKT}}M_{\mathrm{K}n}^{\mathrm{i}} \tag{S96}$$

APC/C at the *n*-th kinetochore:

$$\frac{\partial A_{\mathrm{K}n}^{\mathrm{a}}}{\partial t}=D_{\mathrm{K}}\nabla^2 A_{\mathrm{K}n}^{\mathrm{a}}-k_{\mathrm{dAwM}}M_{\mathrm{K}n}^{\mathrm{a}}A_{\mathrm{K}n}^{\mathrm{a}}+k_{\mathrm{aAwC}}C_{\mathrm{K}n}^{\mathrm{a}}A_{\mathrm{K}n}^{\mathrm{i}}+k_{\mathrm{aAwX}}X_{\mathrm{K}n}^{\mathrm{a}}A_{\mathrm{K}n}^{\mathrm{i}} \tag{S97}$$

$$\frac{\partial A_{\mathrm{K}n}^{\mathrm{i}}}{\partial t}=D_{\mathrm{K}}\nabla^2 A_{\mathrm{K}n}^{\mathrm{i}}+k_{\mathrm{dAwM}}M_{\mathrm{K}n}^{\mathrm{a}}A_{\mathrm{K}n}^{\mathrm{a}}-k_{\mathrm{aAwC}}C_{\mathrm{K}n}^{\mathrm{a}}A_{\mathrm{K}n}^{\mathrm{i}}-k_{\mathrm{aAwX}}X_{\mathrm{K}n}^{\mathrm{a}}A_{\mathrm{K}n}^{\mathrm{i}} \tag{S98}$$

APC/C flux across the boundary of unattached kinetochore:

$$-\mathbf{n}\cdot\mathbf{\Gamma}_{A_{\mathrm{K}n}^{\mathrm{a}}} = k_{\mathrm{onKTu}}\left(1-\left(A_{\mathrm{K}n}^{\mathrm{a}}+A_{\mathrm{K}n}^{\mathrm{i}}\right)\Big/A_{\mathrm{K}}^{\max}\right)\left(A_0^{\mathrm{a}}+A_1^{\mathrm{a}}+A_{00}^{\mathrm{a}}\right)-k_{\mathrm{offKT}}A_{\mathrm{K}n}^{\mathrm{a}} \tag{S99}$$

$$-\mathbf{n}\cdot\mathbf{\Gamma}_{A_{\mathrm{K}n}^{\mathrm{i}}} = k_{\mathrm{onKTu}}\left(1-\left(A_{\mathrm{K}n}^{\mathrm{a}}+A_{\mathrm{K}n}^{\mathrm{i}}\right)\Big/A_{\mathrm{K}}^{\max}\right)\left(A_0^{\mathrm{i}}+A_1^{\mathrm{i}}+A_{00}^{\mathrm{i}}\right)-k_{\mathrm{offKT}}A_{\mathrm{K}n}^{\mathrm{i}} \tag{S100}$$

$$-\mathbf{n}\cdot\mathbf{\Gamma}_{A_0^{\mathrm{a}}} = -k_{\mathrm{onKTu}}\left(1-\left(A_{\mathrm{K}n}^{\mathrm{a}}+A_{\mathrm{K}n}^{\mathrm{i}}\right)\Big/A_{\mathrm{K}}^{\max}\right)A_0^{\mathrm{a}} \tag{S101}$$

$$-\mathbf{n}\cdot\mathbf{\Gamma}_{A_0^{\mathrm{i}}} = -k_{\mathrm{onKTu}}\left(1-\left(A_{\mathrm{K}n}^{\mathrm{a}}+A_{\mathrm{K}n}^{\mathrm{i}}\right)\Big/A_{\mathrm{K}}^{\max}\right)A_0^{\mathrm{i}} \tag{S102}$$

$$-\mathbf{n}\cdot\mathbf{\Gamma}_{A_1^{\mathrm{a}}} = -k_{\mathrm{onKTu}}\left(1-\left(A_{\mathrm{K}n}^{\mathrm{a}}+A_{\mathrm{K}n}^{\mathrm{i}}\right)\Big/A_{\mathrm{K}}^{\max}\right)A_1^{\mathrm{a}} \tag{S103}$$

$$-\mathbf{n}\cdot\mathbf{\Gamma}_{A_1^{\mathrm{i}}} = -k_{\mathrm{onKTu}}\left(1-\left(A_{\mathrm{K}n}^{\mathrm{a}}+A_{\mathrm{K}n}^{\mathrm{i}}\right)\Big/A_{\mathrm{K}}^{\max}\right)A_1^{\mathrm{i}} \tag{S104}$$

$$-\mathbf{n}\cdot\mathbf{\Gamma}_{A_{00}^{\mathrm{a}}} = -k_{\mathrm{onKTu}}\left(1-\left(A_{\mathrm{K}n}^{\mathrm{a}}+A_{\mathrm{K}n}^{\mathrm{i}}\right)\Big/A_{\mathrm{K}}^{\max}\right)A_{00}^{\mathrm{a}}+k_{\mathrm{offKT}}A_{\mathrm{K}n}^{\mathrm{a}} \tag{S105}$$

$$-\mathbf{n}\cdot\mathbf{\Gamma}_{A_{00}^{\mathrm{i}}} = -k_{\mathrm{onKTu}}\left(1-\left(A_{\mathrm{K}n}^{\mathrm{a}}+A_{\mathrm{K}n}^{\mathrm{i}}\right)\Big/A_{\mathrm{K}}^{\max}\right)A_{00}^{\mathrm{i}}+k_{\mathrm{offKT}}A_{\mathrm{K}n}^{\mathrm{i}} \tag{S106}$$

APC/C flux across the boundary of attached kinetochore:

$$-\mathbf{n}\cdot\mathbf{\Gamma}_{A_{\mathrm{K}n}^{\mathrm{a}}} = k_{\mathrm{onKTt}}\left(1-\left(A_{\mathrm{K}n}^{\mathrm{a}}+A_{\mathrm{K}n}^{\mathrm{i}}\right)\Big/A_{\mathrm{K}}^{\max}\right)\left(A_0^{\mathrm{a}}+A_1^{\mathrm{a}}+A_{00}^{\mathrm{a}}\right)-\left(k_{\mathrm{offKT}}+k_{\mathrm{DoffKT}}\right)A_{\mathrm{K}n}^{\mathrm{a}} \tag{S107}$$

$$-\mathbf{n}\cdot\mathbf{\Gamma}_{A_{\mathrm{K}n}^{\mathrm{i}}} = k_{\mathrm{onKTt}}\left(1-\left(A_{\mathrm{K}n}^{\mathrm{a}}+A_{\mathrm{K}n}^{\mathrm{i}}\right)\Big/A_{\mathrm{K}}^{\max}\right)\left(A_0^{\mathrm{i}}+A_1^{\mathrm{i}}+A_{00}^{\mathrm{i}}\right)-\left(k_{\mathrm{offKT}}+k_{\mathrm{DoffKT}}\right)A_{\mathrm{K}n}^{\mathrm{i}} \tag{S108}$$

$$-\mathbf{n}\cdot\mathbf{\Gamma}_{A_0^{\mathrm{a}}} = -k_{\mathrm{onKTt}}\left(1-\left(A_{\mathrm{K}n}^{\mathrm{a}}+A_{\mathrm{K}n}^{\mathrm{i}}\right)\Big/A_{\mathrm{K}}^{\max}\right)A_0^{\mathrm{a}} \tag{S109}$$

$$-\mathbf{n}\cdot\mathbf{\Gamma}_{A_0^{\mathrm{i}}} = -k_{\mathrm{onKTt}}\left(1-\left(A_{\mathrm{K}n}^{\mathrm{a}}+A_{\mathrm{K}n}^{\mathrm{i}}\right)\Big/A_{\mathrm{K}}^{\max}\right)A_0^{\mathrm{i}} \tag{S110}$$

$$-\mathbf{n}\cdot\mathbf{\Gamma}_{A_1^{\mathrm{a}}} = -k_{\mathrm{onKTt}}\left(1-\left(A_{\mathrm{K}n}^{\mathrm{a}}+A_{\mathrm{K}n}^{\mathrm{i}}\right)\Big/A_{\mathrm{K}}^{\max}\right)A_1^{\mathrm{a}}+k_{\mathrm{DoffKT}}A_{\mathrm{K}n}^{\mathrm{a}} \tag{S111}$$

$$-\mathbf{n}\cdot\mathbf{\Gamma}_{A_1^{\mathrm{i}}} = -k_{\mathrm{onKTt}}\left(1-\left(A_{\mathrm{K}n}^{\mathrm{a}}+A_{\mathrm{K}n}^{\mathrm{i}}\right)\Big/A_{\mathrm{K}}^{\max}\right)A_1^{\mathrm{i}}+k_{\mathrm{DoffKT}}A_{\mathrm{K}n}^{\mathrm{i}} \tag{S112}$$

$$-\mathbf{n}\cdot\mathbf{\Gamma}_{A_{00}^{\mathrm{a}}} = -k_{\mathrm{onKTt}}\left(1-\left(A_{\mathrm{K}n}^{\mathrm{a}}+A_{\mathrm{K}n}^{\mathrm{i}}\right)\Big/A_{\mathrm{K}}^{\max}\right)A_{00}^{\mathrm{a}}+k_{\mathrm{offKT}}A_{\mathrm{K}n}^{\mathrm{a}} \tag{S113}$$

$$-\mathbf{n}\cdot\mathbf{\Gamma}_{\mathrm{A}_{00}^{\mathrm{i}}} = -k_{\mathrm{onKTt}}\left(1-\left(A_{\mathrm{K}n}^{\mathrm{a}}+A_{\mathrm{K}n}^{\mathrm{i}}\right)\Big/A_{\mathrm{K}}^{\max}\right)A_{00}^{\mathrm{i}}+k_{\mathrm{offKT}}A_{\mathrm{K}n}^{\mathrm{i}} \tag{S114}$$

Cyclin B at the n-th kinetochore:

$$\frac{\partial C_{\mathrm{K}n}^{\mathrm{a}}}{\partial t} = D_{\mathrm{C}} \nabla^2 C_{\mathrm{K}n}^{\mathrm{a}} - k_{\mathrm{dCwA}} \frac{\left(f_{\mathrm{A}} / K_{\mathrm{mdCwA}}\right)^H}{1 + \left(f_{\mathrm{A}} / K_{\mathrm{mdCwA}}\right)^H} A_{\mathrm{K}n}^{\mathrm{a}} \cdot C_{\mathrm{K}n}^{\mathrm{a}} \tag{S115}$$

where $f_{\mathrm{A}} = A_{\mathrm{K}n}^{\mathrm{a}} \Big/ \left(A_{\mathrm{K}n}^{\mathrm{a}} + A_{\mathrm{K}n}^{\mathrm{i}} \right)$.

Cyclin B flux across the boundary of unattached kinetochore:

$$-\mathbf{n} \cdot \mathbf{\Gamma}_{C_{\mathrm{K}n}^{\mathrm{a}}} = k_{\mathrm{onKTu}} \left(1 - C_{\mathrm{K}n}^{\mathrm{a}} / C_{\mathrm{K}}^{\max}\right)\left(C_0^{\mathrm{a}} + C_1^{\mathrm{a}} + C_{00}^{\mathrm{a}}\right) - k_{\mathrm{offKT}} C_{\mathrm{K}n}^{\mathrm{a}} \tag{S116}$$

$$-\mathbf{n} \cdot \mathbf{\Gamma}_{C_0^{\mathrm{a}}} = -k_{\mathrm{onKTu}} \left(1 - C_{\mathrm{K}n}^{\mathrm{a}} / C_{\mathrm{K}}^{\max}\right) C_0^{\mathrm{a}} \tag{S117}$$

$$-\mathbf{n} \cdot \mathbf{\Gamma}_{C_1^{\mathrm{a}}} = -k_{\mathrm{onKTu}} \left(1 - C_{\mathrm{K}n}^{\mathrm{a}} / C_{\mathrm{K}}^{\max}\right) C_1^{\mathrm{a}} \tag{S118}$$

$$-\mathbf{n} \cdot \mathbf{\Gamma}_{C_{00}^{\mathrm{a}}} = -k_{\mathrm{onKTu}} \left(1 - C_{\mathrm{K}n}^{\mathrm{a}} / C_{\mathrm{K}}^{\max}\right) C_{00}^{\mathrm{a}} + k_{\mathrm{offKT}} C_{\mathrm{K}n}^{\mathrm{a}} \tag{S119}$$

Cyclin B flux across the boundary of attached kinetochore:

$$-\mathbf{n} \cdot \mathbf{\Gamma}_{C_{\mathrm{K}n}^{\mathrm{a}}} = k_{\mathrm{onKTt}} \left(1 - C_{\mathrm{K}n}^{\mathrm{a}} / C_{\mathrm{K}}^{\max}\right)\left(C_0^{\mathrm{a}} + C_1^{\mathrm{a}} + C_{00}^{\mathrm{a}}\right) - \left(k_{\mathrm{offKT}} + k_{\mathrm{DoffKT}}\right) C_{\mathrm{K}n}^{\mathrm{a}} \tag{S120}$$

$$-\mathbf{n} \cdot \mathbf{\Gamma}_{C_0^{\mathrm{a}}} = -k_{\mathrm{onKTt}} \left(1 - C_{\mathrm{K}n}^{\mathrm{a}} / C_{\mathrm{K}}^{\max}\right) C_0^{\mathrm{a}} \tag{S121}$$

$$-\mathbf{n} \cdot \mathbf{\Gamma}_{C_1^{\mathrm{a}}} = -k_{\mathrm{onKTt}} \left(1 - C_{\mathrm{K}n}^{\mathrm{a}} / C_{\mathrm{K}}^{\max}\right) C_1^{\mathrm{a}} + k_{\mathrm{DoffKT}} C_{\mathrm{K}n}^{\mathrm{a}} \tag{S122}$$

$$-\mathbf{n} \cdot \mathbf{\Gamma}_{C_{00}^{\mathrm{a}}} = -k_{\mathrm{onKTt}} \left(1 - C_{\mathrm{K}n}^{\mathrm{a}} / C_{\mathrm{K}}^{\max}\right) C_{00}^{\mathrm{a}} + k_{\mathrm{offKT}} C_{\mathrm{K}n}^{\mathrm{a}} \tag{S123}$$

Trigger factor X at the n-th kinetochore:

$$\frac{\partial X_{\mathrm{K}n}^{\mathrm{a}}}{\partial t} = D_{\mathrm{K}} \nabla^2 X_{\mathrm{K}n}^{\mathrm{a}} - k_{\mathrm{dX}} X_{\mathrm{K}n}^{\mathrm{a}} + \left(k_{\mathrm{aXwC}} C_{\mathrm{K}n}^{\mathrm{a}} + k_{\mathrm{aXwX}} \mathrm{GK}\left(k_1 X_{\mathrm{K}n}^{\mathrm{a}}, k_2, J_1, J_2\right)\right) X_{\mathrm{K}n}^{\mathrm{i}} \tag{S124}$$

$$\frac{\partial X_{\mathrm{K}n}^{\mathrm{i}}}{\partial t} = D_{\mathrm{K}} \nabla^2 X_{\mathrm{K}n}^{\mathrm{i}} + k_{\mathrm{dX}} X_{\mathrm{K}n}^{\mathrm{a}} - \left(k_{\mathrm{aXwC}} C_{\mathrm{K}n}^{\mathrm{a}} + k_{\mathrm{aXwX}} \mathrm{GK}\left(k_1 X_{\mathrm{K}n}^{\mathrm{a}}, k_2, J_1, J_2\right)\right) X_{\mathrm{K}n}^{\mathrm{i}} \tag{S125}$$

Trigger factor X flux across the boundary of kinetochore:

$$-\mathbf{n}\cdot\boldsymbol{\Gamma}_{X_{\mathrm{K}n}^{\mathrm{a}}}=k_{\mathrm{onKTX}}X_{00}^{\mathrm{a}}-k_{\mathrm{offKTX}}X_{\mathrm{K}n}^{\mathrm{a}} \tag{S126}$$

$$-\mathbf{n}\cdot\boldsymbol{\Gamma}_{X_{\mathrm{K}n}^{\mathrm{i}}}=k_{\mathrm{onKTX}}X_{00}^{\mathrm{i}}-k_{\mathrm{offKTX}}X_{\mathrm{K}n}^{\mathrm{i}} \tag{S127}$$

$$-\mathbf{n}\cdot\boldsymbol{\Gamma}_{X_{00}^{\mathrm{a}}}=-k_{\mathrm{onKTX}}X_{00}^{\mathrm{a}}+k_{\mathrm{offKTX}}X_{\mathrm{K}n}^{\mathrm{a}} \tag{S128}$$

$$-\mathbf{n}\cdot\boldsymbol{\Gamma}_{X_{00}^{\mathrm{i}}}=-k_{\mathrm{onKTX}}X_{00}^{\mathrm{i}}+k_{\mathrm{offKTX}}X_{\mathrm{K}n}^{\mathrm{i}} \tag{S129}$$

Initially, *M, A, C* and *X* assume the diffusive state and distribute homogeneously in the cytoplasm with their bulk concentrations given in **Supplementary Table 2**. The initial chemical activities are set as $M^{\mathrm{a}}/\left(M^{\mathrm{a}}+M^{\mathrm{i}}\right)=0.9$, $A^{\mathrm{a}}/\left(A^{\mathrm{a}}+A^{\mathrm{i}}\right)=0.01$, $X^{\mathrm{a}}/\left(X^{\mathrm{a}}+X^{\mathrm{i}}\right)=0.01$. These initial conditions are flexible, because the system is allowed to relax to steady state before kinetochore attachments are implemented. The biochemical parameters are given in **Supplementary Table 2**. The details of the biochemical circuit are not important for our conclusion as long as SAC silencing is triggered by a threshold concentration of a dynein-transported protein at the spindle pole.

Method for stochastic simulation

Because the model focuses on the spindle-mediated spatiotemporal dynamics and its role in triggering SAC silencing at the spindle pole, our stochastic simulation also focuses on how the noise in the spatiotemporal dynamics affect the timing of SAC silencing, as was done in the previous work [1, 2]. Note, because the number of cyclin B proteins, or any of the key SAC proteins discussed in this work, at the spindle pole is at least 10^4, the number fluctuation is likely low. However, fluctuations in the biochemical activity of a protein could stem from fluctuations in microtubule density, protein conformation, protein complex assembly/disassembly, protein expression level, etc. Here, we simply lump all these stochastic factors into a relative noise in the biochemical reaction rates controlled by cyclin B. In addition, the noise comes with a characteristic memory time. Intracellular noises usually assume multiple time scales. Fast noises average out in the long term and have little effects on slow processes. But slow noises on time scales comparable to the process of interest could significantly affect the process. Particularly, the on and off processes of SAC proteins at the spindle pole happen on the order of sub minute. Therefore, we impose on the noise term a memory effect of 0.2 min. Specifically,

we implement a multiplier, $(1 + \xi(t))$, to the $C_{\mathrm{t}}^{\mathrm{a}}$ term in all the equations at the spindle pole (Eqs. (S55) -(S70), (S75)-(S78)). For example, the APC/C dynamics at the spindle pole was revised to Eq. (S130).

$$\frac{\partial A^{\mathrm{a}}}{\partial t} = \text{Spatial regulation} - k_{\mathrm{dAwM}} M^{\mathrm{a}} A^{\mathrm{a}} + k_{\mathrm{aAwC}} C^{\mathrm{a}} \left(1 + \xi(t)\right) A^{\mathrm{i}} + k_{\mathrm{aAwX}} X^{\mathrm{a}} A^{\mathrm{i}} \qquad \text{(S130)}$$

$\xi(t)$ represents a 20% relative noise level with a 0.2-min memory. For easy numeric implementation, $\xi(t)$ was defined as a piecewise function with time segments of 0.2 min; within each segment, a normally distributed random value with standard deviation 0.2 was drawn. Because stochastic simulation is computationally expensive, we also followed the method developed in [1, 2] and performed the stochastic simulation on a 2D-axisymmetric reduced geometry (**Supplementary Figure 10**), instead of the 3D geometry.

Supplementary Table 1. Parameters for spatiotemporal dynamics model.

Parameter	Meaning	Value	Source/Reason
R_{KT}	Radius of kinetochore	0.5 μm	[1]
D_{Dyn}	Cytoplasmic diffusion coefficient of microtubule-unbound transport-active proteins	2 $\mu m^2\ s^{-1}$	~ Diffusion coefficient of dynein due to huge size of dynein; inferred from diffusion coefficient of APC/C [14].
D_{MT}	Diffusion coefficient of microtubule-bound transport-active proteins along microtubule	0.05 $\mu m^2\ s^{-1}$	[15, 16]
D_{Y}	Diffusion coefficient of transport-inactive proteins	2 $\mu m^2\ s^{-1}$	~ Diffusion coefficient of APC/C [14]. No significant difference in model results between spindle pole accumulation of APC/C (D_{A} = 2 $\mu m^2\ s^{-1}$) and SAC protein (D_{M} = 20 $\mu m^2\ s^{-1}$) [1].<br>In the extended model, this number will be replaced by D_{A}, D_{M} and D_{C}, respectively (see **Table S2**).
D_{P}	Diffusion coefficient of spindle pole-bound proteins	2 $\mu m^2\ s^{-1}$	Sufficiently diffusive to homogenize concentration in spindle pole.
D_{K}	Diffusion coefficient of kinetochore-bound proteins	2 $\mu m^2\ s^{-1}$	Sufficiently diffusive to homogenize concentration in kinetochore.
V	Processive velocity of microtubule-bound transport-active proteins along microtubule	0.1 $\mu m\ s^{-1}$	0.06~0.3 $\mu m\ s^{-1}$ [9, 17].
k_{offMT}	Dissociation rate of transport-active proteins from microtubule	1 s^{-1}	*In vitro* unbinding rate 0.05 ~ 1 s^{-1} [18, 19].
k_{onMT}	Association rate of transport-active proteins to microtubule (per microtubule density)	0.17 $\mu m^2\ s^{-1}$	[1, 20].
$k_{\mathrm{offMT}}^{\mathrm{SP}}$	Dissociation rate of transport-active proteins from microtubule in spindle pole	40 s^{-1}	Immediate dissociation from the microtubule once entering the spindle pole.
$k_{\mathrm{onMT}}^{\mathrm{SP}}$	Association rate of transport-active proteins to microtubule in spindle pole	0	Immediate dissociation from the microtubule once entering the spindle pole.
k_{offKT}	Turnover rate from unattached kinetochore	0.2 s^{-1}	1~60 s turnover time of SAC proteins at the unattached kinetochore [9, 21-23].
$Y_{\mathrm{K}}^{\max}$	Saturating concentration on unattached kinetochore	100 (relative to bulk concentration)	Single unattached kinetochore sequesters ~0.05% of total cytoplasmic amount of SAC components [21]; ratio between kinetochore volume and cell volume ~ 10^{-5} → kinetochore concentration ~ 10^2 bulk average in cell.
k_{onKTu}	Recruitment rate onto unattached kinetochore	200 s^{-1}	Sufficiently large to saturate the unattached kinetochore.
k_{onKTt}	Recruitment rate onto attached kinetochore	0.4 s^{-1}	$\ll k_{\mathrm{onKTu}}$ due to attachment/tension induced change in kinase effect.
k_{DoffKT}	Release rate of poleward transport-active proteins from attached kinetochore	20 s^{-1}	$k_{\mathrm{DoffKT}} > k_{\mathrm{onKTt}}$ such that the attached kinetochores do not accumulate protein.
k_{offSP}	Unbinding rate of proteins from spindle pole	0.0333 s^{-1}	~ 30s turnover time at spindle pole [24].
k_{onSP}	Binding rate of proteins to spindle pole	1.8 s^{-1}	Combined with R_{SP} = 0.5 μm to make spindle pole accumulation ~ 5 times the accumulation on single unattached kinetochore [25].
U_{ext}	Sequestration potential around the spindle boundary	2 k_BT, 1 μm width	[1]

Supplementary Table 2. Biochemical parameters. Note that the parameters for trigger factor X (highlighted in blue) are highly generic, with the sole purpose of generating an irreversible toggle switch triggered by appropriate threshold signal.

Parameter	Meaning	Value	Source/Reason
D_A	Diffusion coefficient of APC/C (replacing D_Y in transport-only model)	2 μm^2 s^{-1}	[14]
D_M	Diffusion coefficient of SAC proteins (replacing D_Y in transport-only model)	25 μm^2 s^{-1}	cytoplasmic GFP ~ 25 μm^2 s^{-1} [26, 27] × (GFP weight ~27kD / Mad2 weight ~ 28 kD)$^{1/3}$.
D_C	Diffusion coefficient of cyclin B (replacing D_Y in transport-only model)	20 μm^2 s^{-1}	cytoplasmic GFP ~ 25 μm^2 s^{-1} [26, 27] × (GFP weight ~27kD / cyclin B-CDK1 weight ~ 85 kD)$^{1/3}$.
$[APC/C]_0$	Bulk concentration of APC/C	100 nM	APC2 80 nM [28]; Cdc20 80~280 nM [14, 28, 29].
$[SAC]_0$	Bulk concentration of SAC protein	100 nM	Mad2 100~230 nM, BubR1 90~127 nM [28-30]; Bub1 100 nM ([21], Supplementary Data).
$[\text{cyclin B}]_0$	Bulk concentration of cyclin B	1 μM	[31]
k_{dAwM}	Deactivation rate of APC/C by SAC	200 min^{-1} μM^{-1}	APC/C deactivation rate mediated by 100 nM intracellular SAC ranges from 0.05 min^{-1} [14] to 400 min^{-1} ([32], k_{asmcc} $[Cdc20]_T$).
k_{aAwC}	Activation rate of APC/C by cyclin B	0	[1]
k_{aAwX}	Activation rate of APC/C by X	30 min^{-1}	[1]; sufficiently large to support propagation of APC/C activity from spindle pole.
k_{aMKT}	Direct SAC activation rate at kinetochore	10^{-3} min^{-1}	< ~60 c-Mad2 per kinetochore per sec [33] ÷ 100 nM SAC = 0.2 min^{-1}
k^0_{aMCat}	Catalyzed activation rate of SAC when all kinetochores are unattached	2 min^{-1} μM^{-1}	SAC reactivation in ~5 min after laser-induced detachment of one kinetochore [34] ~ 0.2 min^{-1} μM^{-1} at the stage of one unattached kinetochore.
$k^{N_{KT}}_{aMCat}$	Catalyzed activation rate of SAC left when all kinetochores are attached	0.4 min^{-1} μM^{-1}	20% activation rate left to ensure robust SAC activity when one unattached kinetochore remains; cf. [1].
τ_{DaM}	Relaxation time for the decay of catalyzed activation rate of SAC upon each kinetochore attachment	20 min	2.5 min [35]; 30 min [36, 37].
k_{dMwA}	Deactivation rate of SAC proteins by APC/C	50 min^{-1} μM^{-1}	$k_{imad,c20}$ $[Cdc20]_T$ = 10 min^{-1} [32].
k_{dCwA}	Cyclin B degradation rate with active APC/C	10 min^{-1} μM^{-1}	0.1 ~ 1 min^{-1} [32, 38-40] ÷100 nM APC/C → 1~10 min^{-1} μM^{-1}.
H	Hill coefficient for cyclin B degradation	10	[1]
K_{mdCwA}	Transitional APC/C activity on the effect of cyclin B	0.6	[1]
k_{sC}	Cyclin B synthesis rate	0	[1]
k_{offKTX}	Turnover rate of X from kinetochore	0.2 s^{-1}	Same as the transported components (**Table S1**).
k_{onKTX}	Recruitment rate of X onto kinetochore	2 s^{-1}	Arbitrary weak binding affinity at the kinetochore. Weaker than that at the spindle poles. Resultant kinetochore concentration not sufficient to trigger self-activation.
k_{offSPX}	Turnover rate of X from spindle pole	0.0333 s^{-1}	Same as the transported components (**Table S1**).
k_{onSPX}	Binding rate of X to spindle pole	3.33 s^{-1}	Combined with k_{offSPX} to make the spindle pole concentration of X ~ 100 times the bulk concentration.
D_X	Diffusion coefficient of X	20 μm^{2}s^{-1}	Diffusion coefficient of proteins in cytoplasm 1~30 μm^2 s^{-1} [26, 41-43].
$[X]_0$	Bulk concentration of X	1 a.u.	[1]
k_{aXwC}	Activation rate of X by cyclin B	DLD-1: 2×10^{-3} min^{-1} μM^{-1}<br>RPE-1: 3.8×10^{-3} min^{-1} μM^{-1}	X activation triggered by a cyclin B level at spindle pole between the final and penultimate kinetochore attachments. Chosen to match average metaphase duration measured in the parental DLD-1 cells (**Fig. 3D**) and parental RPE-1 cells (**Fig. S3C**), respectively.
k_{aXwX}	Auto-activation rate of X	10 min^{-1}	Generic toggle switch motif [44].
k_{dX}	Deactivation rate of X	10 min^{-1}	
k_1	Coefficient of Goldbeter-Koshland function	0.01	
k_2	Coefficient of Goldbeter-Koshland function	0.1	
J_1	Coefficient of Goldbeter-Koshland function	0.05	
J_2	Coefficient of Goldbeter-Koshland function	0.05	

Supplementary References

1. Chen, J. and J. Liu, *Spatial-temporal model for silencing of the mitotic spindle assembly checkpoint.* Nat Commun, 2014. **5**: p. 4795.
2. Chen, J. and J. Liu, *Erroneous Silencing of the Mitotic Checkpoint by Aberrant Spindle Pole-Kinetochore Coordination.* Biophys J, 2015. **109**(11): p. 2418-35.
3. Chen, J. and J. Liu, *Spindle Size Scaling Contributes to Robust Silencing of Mitotic Spindle Assembly Checkpoint.* Biophys J, 2016. **111**(5): p. 1064-77.
4. Lince-Faria, M., et al., *Spatiotemporal control of mitosis by the conserved spindle matrix protein Megator.* J Cell Biol, 2009. **184**(5): p. 647-57.
5. Zheng, Y., *A membranous spindle matrix orchestrates cell division.* Nat Rev Mol Cell Biol, 2010. **11**(7): p. 529-35.
6. Johansen, K.M., et al., *Do nuclear envelope and intranuclear proteins reorganize during mitosis to form an elastic, hydrogel-like spindle matrix?* Chromosome Res, 2011. **19**(3): p. 345-65.
7. Wolf, K.W., *Mitotic and Meiotic Spindles from 2 Insect Orders, Lepidoptera and Diptera, Differ in Terms of Microtubule and Membrane Content.* Journal of Cell Science, 1990. **97**: p. 91-100.
8. Steuer, E.R., et al., *Localization of cytoplasmic dynein to mitotic spindles and kinetochores.* Nature, 1990. **345**(6272): p. 266-8.
9. Famulski, J.K., et al., *Dynein/Dynactin-mediated transport of kinetochore components off kinetochores and onto spindle poles induced by nordihydroguaiaretic acid.* PLoS One, 2011. **6**(1): p. e16494.
10. Chen, Q., et al., *Cyclin B1 is localized to unattached kinetochores and contributes to efficient microtubule attachment and proper chromosome alignment during mitosis.* Cell Research, 2008. **18**(2): p. 268-280.
11. Felix, M.A., et al., *Triggering of cyclin degradation in interphase extracts of amphibian eggs by cdc2 kinase.* Nature, 1990. **346**(6282): p. 379-82.
12. Luca, F.C., et al., *Both cyclin A delta 60 and B delta 97 are stable and arrest cells in M-phase, but only cyclin B delta 97 turns on cyclin destruction.* EMBO J, 1991. **10**(13): p. 4311-20.
13. Lorca, T., et al., *Cyclin A-cdc2 kinase does not trigger but delays cyclin degradation in interphase extracts of amphibian eggs.* J Cell Sci, 1992. **102 (Pt 1)**: p. 55-62.
14. Wang, Z.F., et al., *In vivo quantitative studies of dynamic intracellular processes using fluorescence correlation spectroscopy.* Biophysical Journal, 2006. **91**(1): p. 343-351.
15. Wang, Z. and M.P. Sheetz, *One-dimensional diffusion on microtubules of particles coated with cytoplasmic dynein and immunoglobulins.* Cell Struct Funct, 1999. **24**(5): p. 373-83.
16. Ross, J.L., et al., *Processive bidirectional motion of dynein-dynactin complexes in vitro.* Nat Cell Biol, 2006. **8**(6): p. 562-70.
17. Heald, R., et al., *Spindle assembly in Xenopus egg extracts: respective roles of centrosomes and microtubule self-organization.* J Cell Biol, 1997. **138**(3): p. 615-28.
18. King, S.J. and T.A. Schroer, *Dynactin increases the processivity of the cytoplasmic dynein motor.* Nat Cell Biol, 2000. **2**(1): p. 20-4.
19. Reck-Peterson, S.L., et al., *Single-molecule analysis of dynein processivity and stepping behavior.* Cell, 2006. **126**(2): p. 335-48.
20. Chen, J., J. Lippincott-Schwartz, and J. Liu, *Intracellular spatial localization regulated by the microtubule network.* PLoS One, 2012. **7**(4): p. e34919.
21. Howell, B.J., et al., *Spindle checkpoint protein dynamics at kinetochores in living cells.* Current Biology, 2004. **14**(11): p. 953-964.
22. Shah, J.V., et al., *Dynamics of centromere and kinetochore proteins; implications for checkpoint signaling and silencing.* Curr Biol, 2004. **14**(11): p. 942-52.

23. Basto, R., et al., *In vivo dynamics of the rough deal checkpoint protein during Drosophila mitosis.* Curr Biol, 2004. **14**(1): p. 56-61.
24. Famulski, J.K., et al., *Stable hZW10 kinetochore residency, mediated by hZwint-1 interaction, is essential for the mitotic checkpoint.* J Cell Biol, 2008. **180**(3): p. 507-20.
25. Howell, B.J., et al., *Cytoplasmic dynein/dynactin drives kinetochore protein transport to the spindle poles and has a role in mitotic spindle checkpoint inactivation.* J Cell Biol, 2001. **155**(7): p. 1159-72.
26. Lippincott-Schwartz, J., E. Snapp, and A. Kenworthy, *Studying protein dynamics in living cells.* Nat Rev Mol Cell Biol, 2001. **2**(6): p. 444-56.
27. Swaminathan, R., C.P. Hoang, and A.S. Verkman, *Photobleaching recovery and anisotropy decay of green fluorescent protein GFP-S65T in solution and cells: cytoplasmic viscosity probed by green fluorescent protein translational and rotational diffusion.* Biophys J, 1997. **72**(4): p. 1900-7.
28. Tang, Z., et al., *Mad2-Independent inhibition of APCCdc20 by the mitotic checkpoint protein BubR1.* Dev Cell, 2001. **1**(2): p. 227-37.
29. Fang, G., *Checkpoint protein BubR1 acts synergistically with Mad2 to inhibit anaphase-promoting complex.* Mol Biol Cell, 2002. **13**(3): p. 755-66.
30. Howell, B.J., et al., *Visualization of Mad2 dynamics at kinetochores, along spindle fibers, and at spindle poles in living cells.* Journal of Cell Biology, 2000. **150**(6): p. 1233-1249.
31. Deibler, R.W. and M.W. Kirschner, *Quantitative reconstitution of mitotic CDK1 activation in somatic cell extracts.* Mol Cell, 2010. **37**(6): p. 753-67.
32. He, E., et al., *System-level feedbacks make the anaphase switch irreversible.* Proc Natl Acad Sci U S A, 2011. **108**(24): p. 10016-21.
33. Ciliberto, A. and J.V. Shah, *A quantitative systems view of the spindle assembly checkpoint.* EMBO J, 2009. **28**(15): p. 2162-73.
34. Dick, A.E. and D.W. Gerlich, *Kinetic framework of spindle assembly checkpoint signalling.* Nat Cell Biol, 2013. **15**(11): p. 1370-7.
35. Doncic, A., E. Ben-Jacob, and N. Barkai, *Evaluating putative mechanisms of the mitotic spindle checkpoint.* Proc Natl Acad Sci U S A, 2005. **102**(18): p. 6332-7.
36. Sear, R.P. and M. Howard, *Modeling dual pathways for the metazoan spindle assembly checkpoint.* Proc Natl Acad Sci U S A, 2006. **103**(45): p. 16758-63.
37. Mistry, H.B., et al., *Modeling the temporal evolution of the spindle assembly checkpoint and role of Aurora B kinase.* Proc Natl Acad Sci U S A, 2008. **105**(51): p. 20215-20.
38. Brito, D.A. and C.L. Rieder, *Mitotic checkpoint slippage in humans occurs via cyclin B destruction in the presence of an active checkpoint.* Curr Biol, 2006. **16**(12): p. 1194-200.
39. Oliveira, R.A., et al., *Cohesin cleavage and Cdk inhibition trigger formation of daughter nuclei.* Nat Cell Biol, 2010. **12**(2): p. 185-92.
40. Luo, X., et al., *The Mad2 spindle checkpoint protein has two distinct natively folded states.* Nat Struct Mol Biol, 2004. **11**(4): p. 338-45.
41. Lang, I., M. Scholz, and R. Peters, *Molecular mobility and nucleocytoplasmic flux in hepatoma cells.* J Cell Biol, 1986. **102**(4): p. 1183-90.
42. Wojcieszyn, J.W., et al., *Diffusion of injected macromolecules within the cytoplasm of living cells.* Proc Natl Acad Sci U S A, 1981. **78**(7): p. 4407-10.
43. Seksek, O., J. Biwersi, and A.S. Verkman, *Translational diffusion of macromolecule-sized solutes in cytoplasm and nucleus.* J Cell Biol, 1997. **138**(1): p. 131-42.
44. Tyson, J.J., K.C. Chen, and B. Novak, *Sniffers, buzzers, toggles and blinkers: dynamics of regulatory and signaling pathways in the cell.* Curr Opin Cell Biol, 2003. **15**(2): p. 221-31.

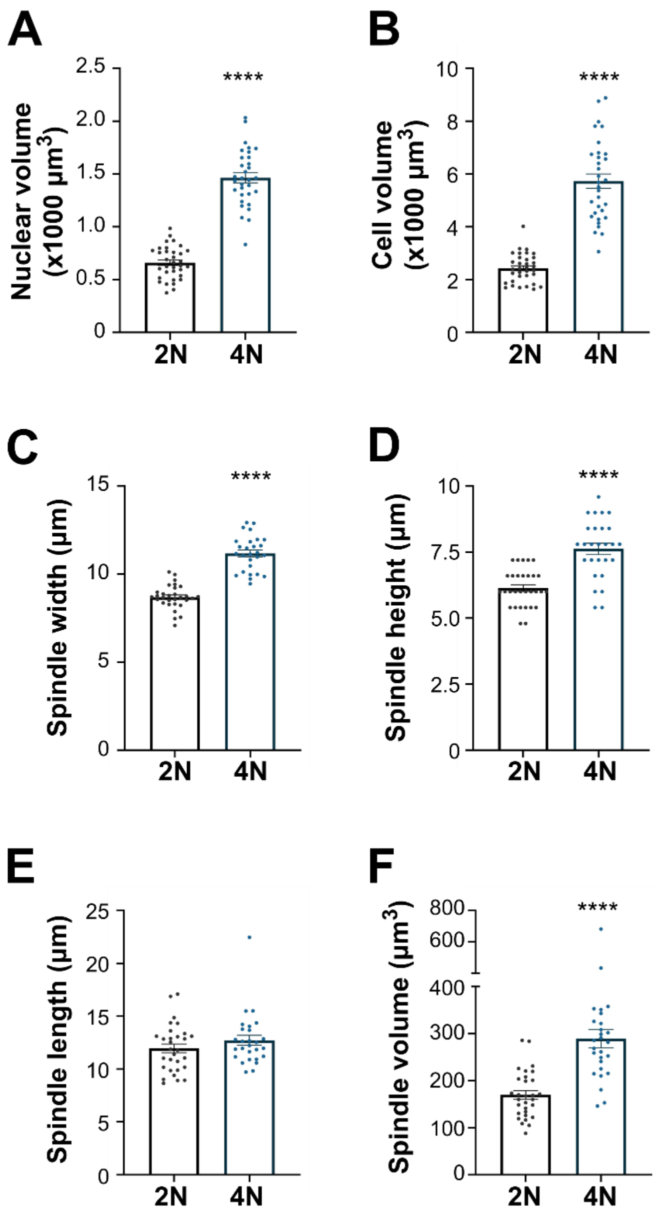

Supplementary Figure 1. Cell, nucleus, and spindle sizes in 2N and 4N RPE-1 cells. (**A-B**) Nuclear (**A**) and cell (**B**) volume in 2N and 4N RPE-1 cells synchronized in G2. Data are reported as mean ± SEM with individual data points from three independent experiments in which a total of 32-35 cells (n=35 and 32 cells, respectively) were analyzed. ****p<0.0001, when compared to the parental 2N RPE-1 cells by Student's t-test. Measurements of mitotic spindle width (**C**), height (**D**), length (**E**), and volume (**F**) reported as mean ± SEM with individual data points from three independent experiments in which a total of 27-30 cells (n=30 and 27 cells, respectively) were analyzed. ****p<0.0001, when compared to the parental 2N RPE-1 cells by Student's t-test.

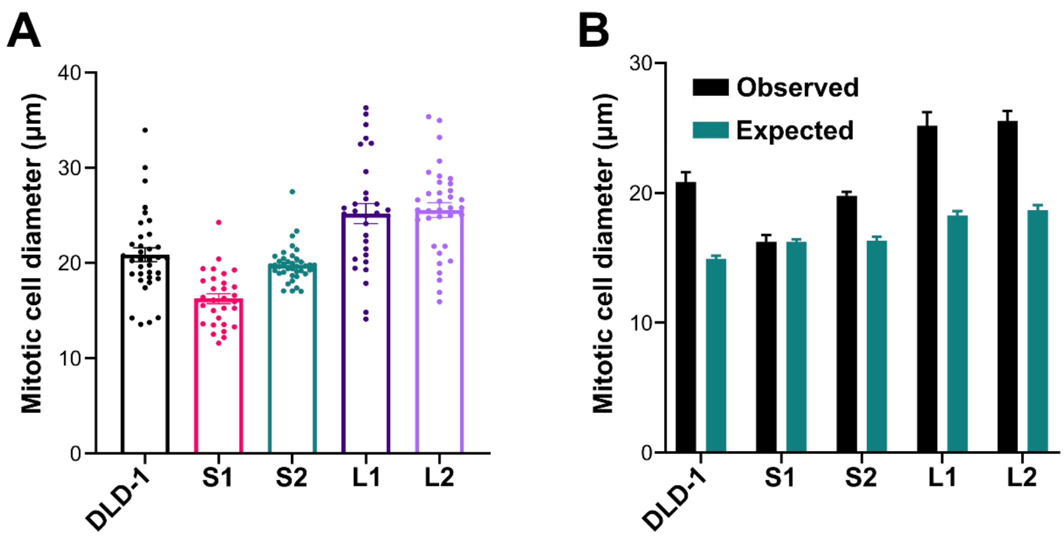

Supplementary Figure 2. Cells from small 4N DLD-1 clones round up more and become more spherical in mitosis. (**A**) Cell diameter measured in metaphase cells along the axis corresponding to spindle length in DLD-1 and the 4N clones and reported as mean ± SEM with individual data points from three independent experiments (n= 35, 30, 40, 30 and 35 cells, respectively). (**B**) The average mitotic cell diameter (from **A**) is compared to a "predicted" diameter that was calculated by using the G2 cell volume measurements (from **Fig. 1C**) and assuming that the cells formed perfect spheres during mitosis. Data are reported as reported as mean ± SEM.

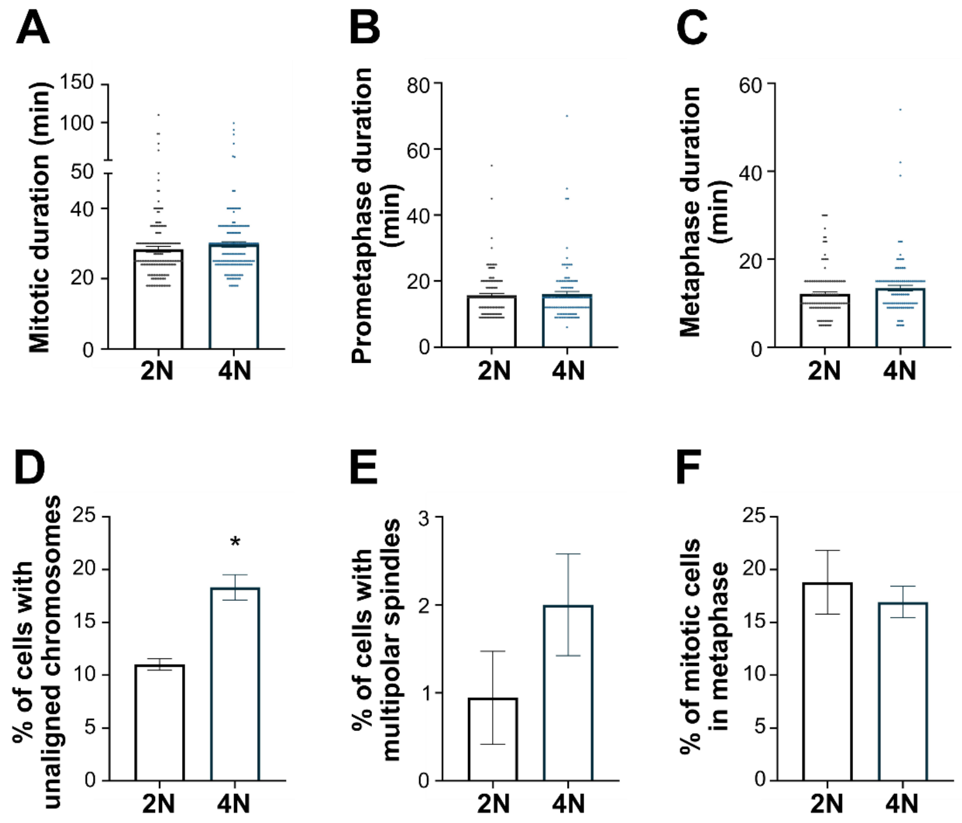

Supplementary Figure 3. 2N and 4N RPE-1 cells display similar mitotic timing and similar frequencies of multipolar spindles. (**A-C**) Quantification of (**A**) mitotic duration (n=187 and 174 cells, respectively), (**B**) prometaphase duration (n=142 and 114 cells, respectively), and (**C**) metaphase duration (n=142 and 114 cells, respectively) in 2N and 4N RPE-1 cells. Data are reported as mean ± SEM with individual data points from three independent experiments. (**D-E**) Quantification of the percentage of late prometaphase-metaphase cells with (**D**) unaligned chromosome(s) and (**E**) multipolar spindles. (**F**) Quantification of the percentage of mitotic cells in metaphase. Graphs in (**D-F**) represent the mean ± SEM of three independent experiments, in which at least 100 cells were scored in each experiment (n>300 for each cell line).

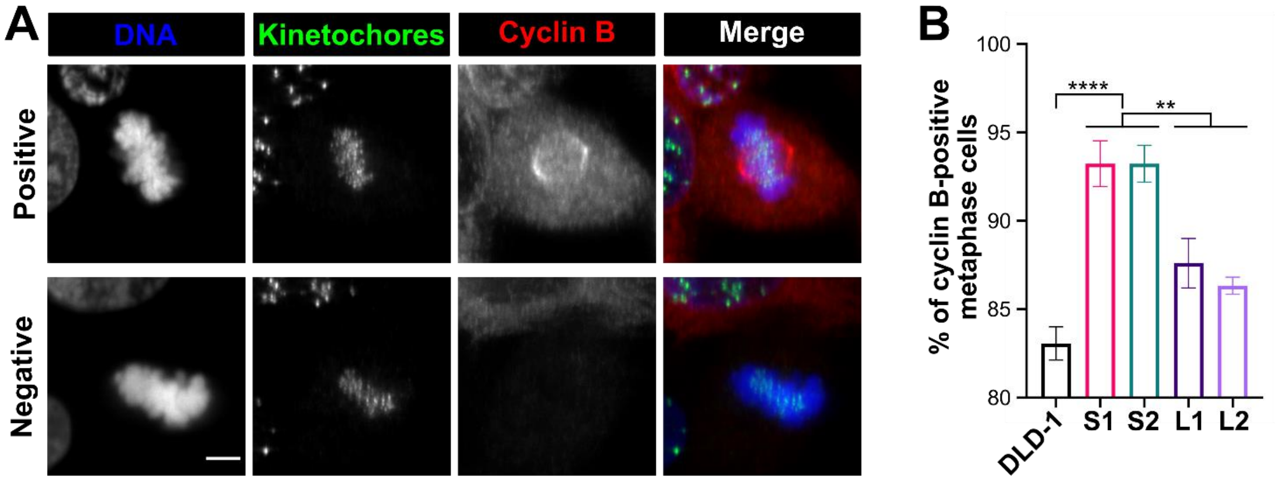

Supplementary Figure 4. A larger fraction of metaphases from the small 4N DLD-1 clones are positive for cyclin B compared to the large 4N clones. (**A**) Examples of cyclin B-positive (top row) and cyclin B-negative (bottom row) metaphase cells. (**B**) Quantification of cyclin B-positive metaphase cells in the parental DLD-1 cells and all 4N DLD-1 clones. The data are reported as mean ± SEM of three independent experiments, in which at least 100 cells were scored in each experiment (n>300 for each cell line).

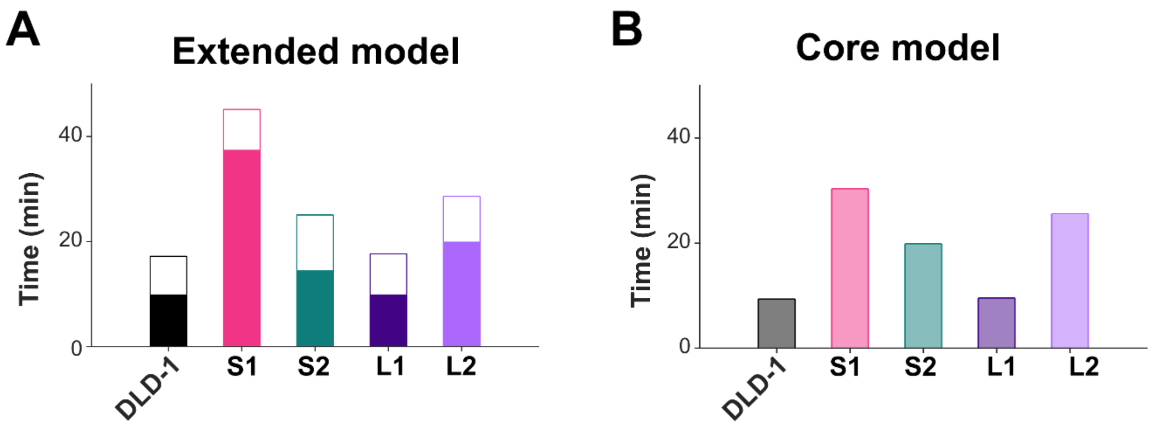

Supplementary Figure 5. Model-predicted metaphase duration critically depends on the triggering time and spindle pole signal level. (**A**) Metaphase duration predicted by the extended model (transport + biochemistry, Eqs. (S39)-(S130)). Solid bars: triggering time, i.e., $t_1 - t_0$ in Fig. 5A. Open bars: propagation time, i.e., $t_2 - t_1$ in Fig. 5A. (**B**) Triggering time predicted by the core model that only describes the spatial regulation of SAC proteins (Eqs. (S22)-(S38)). The triggering time was estimated by the time upon which the predicted spindle pole signal crosses 150. The estimated triggering time in (**B**) is consistent with the triggering time in (**A**).

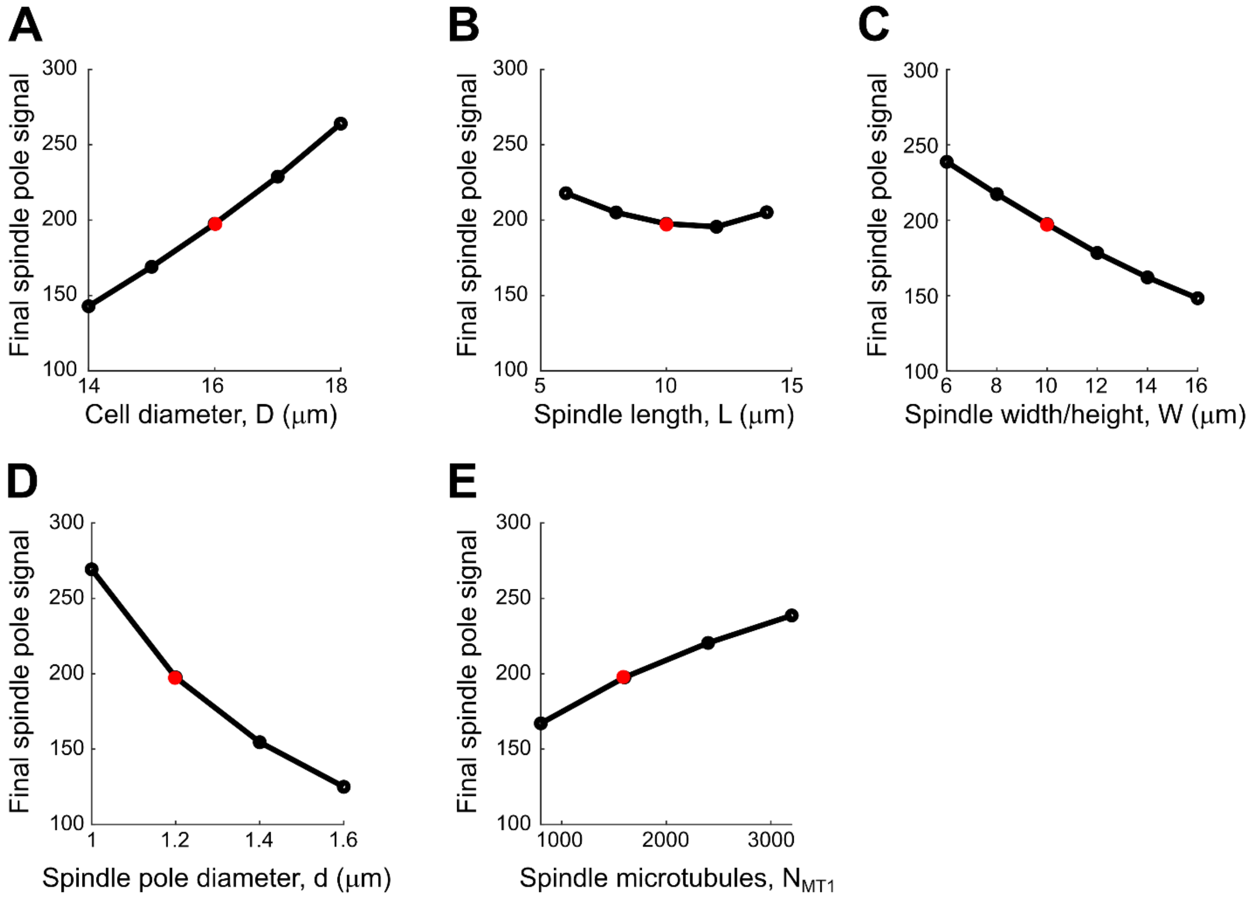

Supplementary Figure 6. Effects of model parameters on spindle pole signal level. Predicted final spindle pole signal as the cell size (**A**), spindle length (**B**), spindle width and/or height (**C**), spindle pole size (**D**) and spindle microtubule abundance (**E**). Results from the core model that only describes the spatial regulation of SAC proteins (Eqs. (S22)-(S38)). Red dots: default parameter values, i.e., $D = 16$ μm, $L = 10$ μm, $W = 10$ μm, $d = 1.2$ μm, $N_{\mathrm{MT1}} = 1600$. All other parameters follow Table S1.

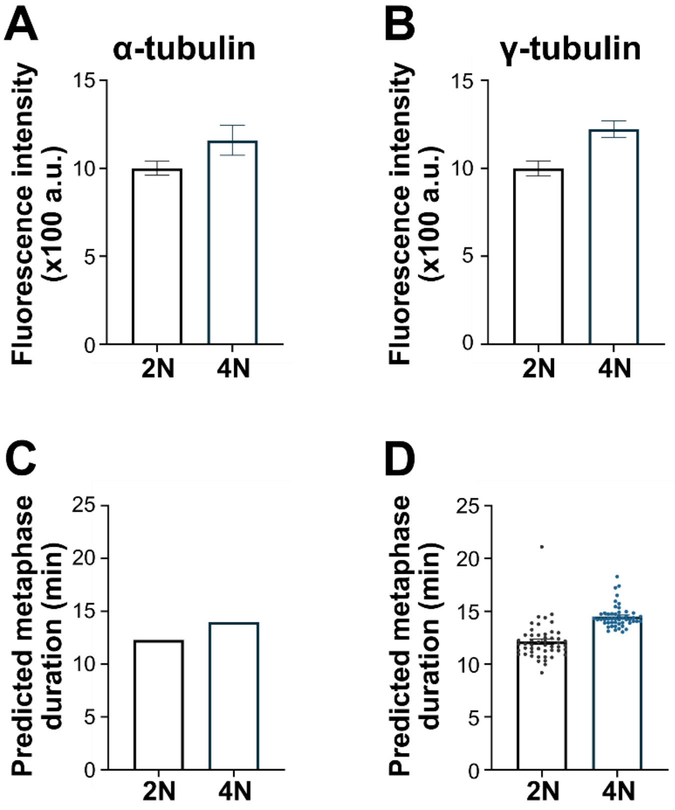

Supplementary Figure 7. Mathematical model predicts observed metaphase durations in 2N and 4N RPE-1 cells. (**A**) Experimentally measured α-tubulin intensity in 2N and 4N RPE-1 cells. (**B**) Experimentally measured γ-tubulin intensity in 2N and 4N RPE-1 cells. (**E, F**) Metaphase durations predicted for 2N and 4N RPE-1 cells without (**C**) and with (**D**) noise. Scatter dots in (**F**) show 50 individual simulation results and bars show the average.

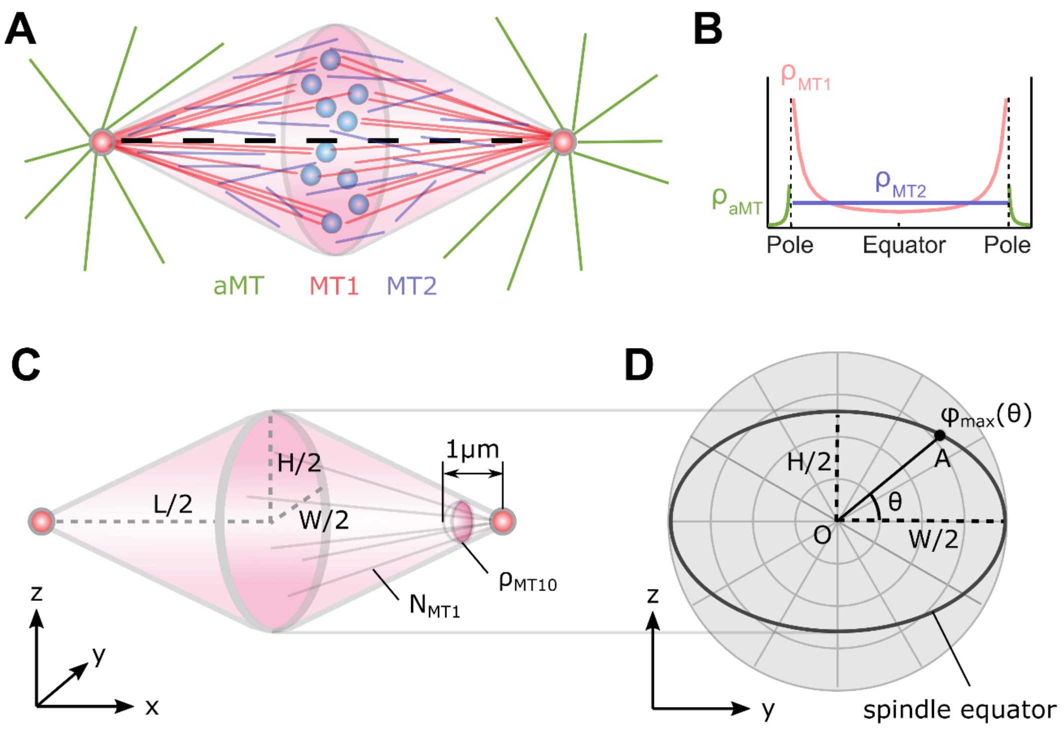

Supplementary Figure 8. Spindle microtubule density and spindle morphology. (**A**) Microtubule subpopulations in the cell. aMT (green): astral microtubules emanating from the spindle pole. MT1 (red): spindle microtubules emanating from the spindle pole. MT2 (blue): dispersed microtubules in the spindle. (**B**) Densities (per unit area) of microtubules in each subpopulation. The densities are shown along the spindle axis and its extension towards the cell boundary (black dashed line in (A)). The aMT and MT1 densities scale inversely with the squared distance from the spindle pole. The MT2 density stays constant within the spindle. (**C**) Spindle dimensions in side view. N_{MT1} is the total number of MT1 microtubules (in both half spindles). ρ_{MT10} is the density of MT1 microtubules crossing the spherical surface 1 μm away from the center of the spindle pole. (**D**) Top view of the spindle equator. φ and θ are the polar and azimuthal angles, respectively, of the spherical coordinate system. $\varphi_{\mathrm{max}}(\theta)$ is the polar angle of the spindle peripheral for a given azimuthal angle, θ.

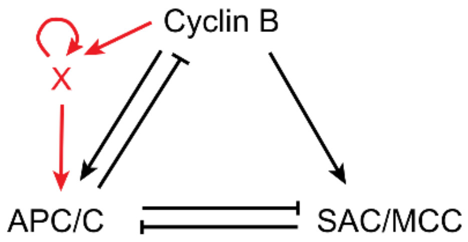

Supplementary Figure 9. Biochemical circuit for SAC. Cyclin B activates both APC/C and SAC/MCC activity. APC/C, in turn, mediates cyclin B degradation. APC/C is also in a mutually inhibitory relation with SAC/MCC. Cyclin B concentration over the threshold turns on the self-activating trigger factor X, which then activates APC/C.

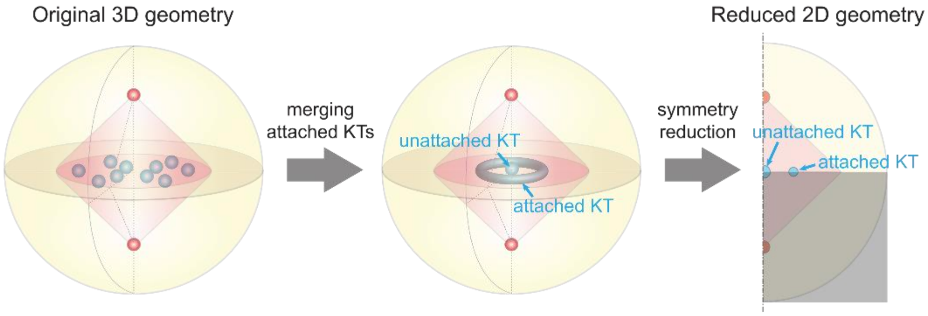

Supplementary Figure 10. 2D axisymmetric geometry for stochastic simulation. To conserve the total fluxes of SAC proteins through the unattached kinetochores, the volume of the central spherical domain must equal the total volume of unattached kinetochores. As shown in [1, 2], the radius of the central domain (unattached KT) should be $r_{\mathrm{uKT}} = \sqrt[3]{N_{\mathrm{uKT}}} R_{\mathrm{KT}}$, where N_{uKT} is the number of unattached kinetochores. Similarly, the volume of the toroid domain (attached KT) must equal the total volume of attached kinetochores. The tube radius of the torus is then $r_{\mathrm{torus}} = \sqrt{6N_{\mathrm{aKT}} R_{\mathrm{KT}}^3 / \pi R_{\mathrm{torus}}}$, where N_{aKT} is the number of attached kinetochores and R_{torus} is the circumferential radius of the torus. To focus on the dynamics before and after attachment of the last kinetochore, we choose $N_{\mathrm{uKT}} = 1$ and $N_{\mathrm{aKT}} = N_{\mathrm{KT}} - 1$.

Supplementary Videos.

Supplementary Video 1. X-axis rotation of three-dimensional DLD-1 metaphase spindle. Volume projection of representative DLD-1 metaphase spindle showing DNA (blue), kinetochores (green), microtubules (red), and centrioles (yellow). Video related to Figure 2A.

Supplementary Video 2. Y-axis rotation of three-dimensional DLD-1 metaphase spindle. Volume projection of representative DLD-1 metaphase spindle showing DNA (blue), kinetochores (green), microtubules (red), and centrioles (yellow). Video related to Figure 2A.

Supplementary Video 3. X-axis rotation of three-dimensional S1 metaphase spindle. Volume projection of representative S1 metaphase spindle showing DNA (blue), kinetochores (green), microtubules (red), and centrioles (yellow). Video related to Figure 2A.

Supplementary Video 4. Y-axis rotation of three-dimensional S1 metaphase spindle. Volume projection of representative S1 metaphase spindle showing DNA (blue), kinetochores (green), microtubules (red), and centrioles (yellow). Video related to Figure 2A.

Supplementary Video 5. X-axis rotation of three-dimensional S2 metaphase spindle. Volume projection of representative S2 metaphase spindle showing DNA (blue), kinetochores (green), microtubules (red), and centrioles (yellow). Video related to Figure 2A.

Supplementary Video 6. Y-axis rotation of three-dimensional S2 metaphase spindle. Volume projection of representative S2 metaphase spindle showing DNA (blue), kinetochores (green), microtubules (red), and centrioles (yellow). Video related to Figure 2A.

Supplementary Video 7. X-axis rotation of three-dimensional L1 metaphase spindle. Volume projection of representative L1 metaphase spindle showing DNA (blue), kinetochores (green), microtubules (red), and centrioles (yellow). Video related to Figure 2A.

Supplementary Video 8. Y-axis rotation of three-dimensional L1 metaphase spindle. Volume projection of representative L1 metaphase spindle showing DNA (blue), kinetochores (green), microtubules (red), and centrioles (yellow). Video related to Figure 2A.

Supplementary Video 9. X-axis rotation of three-dimensional L2 metaphase spindle. Volume projection of representative L2 metaphase spindle showing DNA (blue), kinetochores (green), microtubules (red), and centrioles (yellow). Video related to Figure 2A.

Supplementary Video 10. Y-axis rotation of three-dimensional L2 metaphase spindle. Volume projection of representative L2 metaphase spindle showing DNA (blue), kinetochores (green), microtubules (red), and centrioles (yellow). Video related to Figure 2A.

Supplementary Video 11. Time-lapse video of DLD-1 cell progressing through mitosis. DLD-1 cell imaged by time-lapse phase-contrast microscopy, with imaging starting prior to mitosis and ending after mitotic exit. Yellow arrow(s) point to the mother and daughter cells in the opening and closing frames, respectively. Cell rounding was designated as 0 min. Images were acquired at 3 minute intervals and are played back at 10 fps. Scale bar, 25 μm. Video related to Figure 3A.

Supplementary Video 12. Time-lapse video of S1 cell progressing through mitosis. S1 cell imaged by time-lapse phase-contrast microscopy, with imaging starting prior to mitosis and ending after mitotic exit. Yellow arrow(s) point to the mother and daughter cells in the opening and closing frames, respectively. Cell rounding was designated as 0 min. Images were acquired at 3 minute intervals and are played back at 10 fps. Scale bar, 25 μm. Video related to Figure 3A.

Supplementary Video 13. Time-lapse video of S2 cell progressing through mitosis. S2 cell imaged by time-lapse phase-contrast microscopy, with imaging starting prior to mitosis and ending after mitotic exit. Yellow arrow(s) point to the mother and daughter cells in the opening and closing frames, respectively. Cell rounding was designated as 0 min. Images were acquired at 3 minute intervals and are played back at 10 fps. Scale bar, 25 μm. Video related to Figure 3A.

Supplementary Video 14. Time-lapse video of L1 cell progressing through mitosis. L1 cell imaged by time-lapse phase-contrast microscopy, with imaging starting prior to mitosis and ending after mitotic exit. Yellow arrow(s) point to the mother and daughter cells in the opening and closing frames, respectively. Cell rounding was designated as 0 min. Images were acquired at 3 minute intervals and are played back at 10 fps. Scale bar, 25 μm. Video related to Figure 3A.

Supplementary Video 15. Time-lapse video of L2 cell progressing through mitosis. L2 cell imaged by time-lapse phase-contrast microscopy, with imaging starting prior to mitosis and ending after mitotic exit. Yellow arrow(s) point to the mother and daughter cells in the opening and closing frames, respectively. Cell rounding was designated as 0 min. Images were acquired at 3 minute intervals and are played back at 10 fps. Scale bar, 25 μm. Video related to Figure 3A.
